# Tunable synthesis of heteroleptic zirconium-based porous coordination cages†

**DOI:** 10.1039/d4sc06023g

**Published:** 2024-11-18

**Authors:** Merissa N. Morey, Christine M. Montone, Michael R. Dworzak, Glenn P. A. Yap, Eric D. Bloch

**Affiliations:** a Department of Chemistry, Indiana University Bloomington IN 47405 USA edbloch@iu.edu; b Department of Chemistry & Biochemistry, University of Delaware Newark DE 19716 USA

## Abstract

Zirconium-based porous coordination cages have been widely studied and have shown to be potentially useful for many applications as a result of their tunability and stability, likely as a result of their status as a molecular equivalent to the small 8 Å tetrahedral pores of UiO-66 (Zr_6_(μ_3_-O)_4_(μ_2_-OH)_4_(C_8_O_4_H_4_)_6_). Functional groups attached to these molecular materials endow them with a range of tunable properties. While so-called multivariate MOFs containing multiple types of functional groups on different bridging ligands within a structure are common, incorporating multiple functional moieties in permanently microporous molecular materials has proved challenging. By applying a mixed-ligand, or heteroleptic, synthesis strategy to cage formation, we have designed a straight-forward, one-pot synthesis of 10 Å zirconium-based molecular cages in a basket-shaped, or Zr_12_L_6_, geometry containing 3 : 3 ratios of combinations of two types of functional moieties from 11 different ligand options. Additionally, using more sterically hindered ligands, such as 5-benzyloxybenzene dicarboxylate, we show that ligand size governs the resulting cage geometry. This method allows for multiple functional groups to be incorporated in molecular cages and the ratio of moieties incorporated can be easily controlled. With this strategy in hand, we show that ligands for which zirconium cage syntheses have been elusive, such as 2,5-dihydroxybenzene dicarboxylate, have now been successfully incorporated into porous structures.

## Abstract 2

A mixed-ligand synthesis yields zirconium-based cages with tunable functional group ratios, enabling diverse ligand incorporation, including previously elusive ones, *via* a straightforward one-pot strategy.

## Graphical abstract

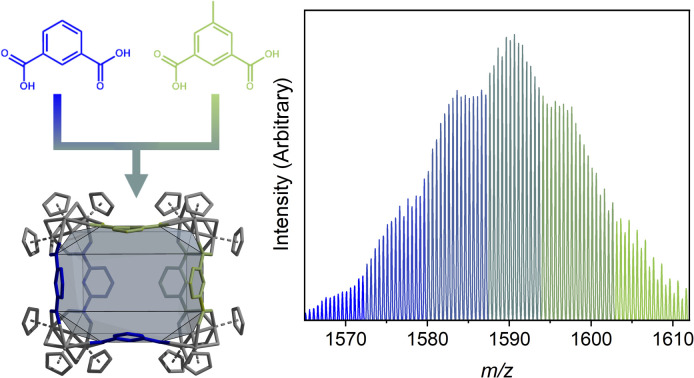

## Introduction

**Fig. 1:**
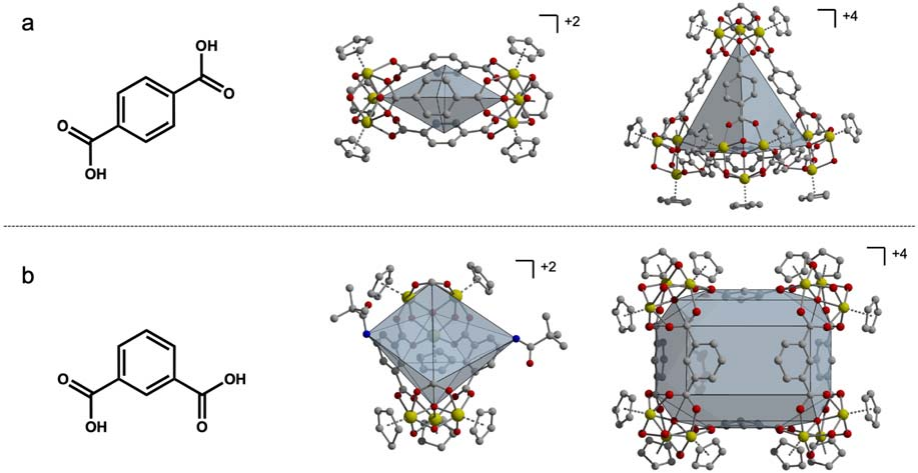
Linear terephthalic acid ligands produce lantern and tetrahedral-shaped cages (a) while bent isophthalic acid ligands produce boat and basket-shaped cages (b). All four geometries are 8–10 Å wide.

As molecular analogs of metal–organic frameworks (MOFs), porous coordination cages (PCCs) have several attractive features including solubility, molecularly precise tunability, and potentially significant inter-cage porosity, although they do typically have lower surface areas than their three-dimensional counterparts.^1–3^ Porous cages are highly versatile structures with a diverse array of properties stemming from their unique molecular architecture. Specifically, zirconium-based cages have displayed several different geometries, which affect their porous nature due to the change in window and pore shape. Additionally, ligands containing reactive functional groups within these systems contribute to gas separation and storage, such as amine-containing cages for the purpose of CO_2_ capture. As a result, porous cages are exceptionally adaptable for a myriad of applications. From their ability to selectively encapsulate guest molecules to their utility in catalysis and gas storage, these cages have become focal points as of recently.^4–7^ For example, functionalized porous cages have been employed in the realization of porous ionic liquids and porous salts where appropriately charged cages can be combined with oppositely charged species to form novel porous phases.^8–10^ Previous research has established that the functionalization of porous materials has a significant impact on their properties.^11–16^ For instance, when incorporating 2-amino-1,4-benzenedicarboxylate ligands into UiO-66, Walton *et al.* observed increased N_2_, CH_4_, and CO_2_ adsorption compared to UiO-66.^11^ Additionally, post synthetic modification (PSM) techniques have previously been utilized to expand the applications of PCCs and have been used to replace functional groups for different functions in molecular cages.^17–20^ Various studies have explored mixed-linker strategies in MOF synthesis, yielding materials with novel properties and diverse potential applications.^21–25^ PCCs are similar to MOFs in terms of tunability, where their properties can be modified by altering ligand functionality.^26–32^ For a subset of both MOFs and cages, varying the functional groups on the ligands connecting metal vertices can afford different phases, as specific pores may not be able to accommodate all functional groups. This effect is largely cage or MOF dependent because of the unique pore and window geometries in each material. We have shown that functional groups can be installed on cuboctahedral cages without impacting the formation of the cage because the functional group lies on the exterior surface of the cage, allowing for minimal ligand–ligand interactions.^1,33,34^ However, in cyclopentadienyl-capped zirconium-based cages, the ligand functional groups either lie at the edge of tetrahedral structures or at the faces of a basket-like structure, both of which can interfere with cage formation due to ligand–ligand interactions and steric hindrance (Fig. 1).^17,35,36^ This can be used to tune cage phase where longer ligands afford lantern-like cages while wider, bulkier ligands result in tetrahedral cages.^27,35,36^ We reasoned that in the synthesis of functionalized cages based on zirconium clusters, mixed ligand (heteroleptic) routes could be leveraged to produce cages with desired functional groups and otherwise inaccessible geometries.

Heteroleptic cage and MOF syntheses have been utilized to achieve materials with targeted properties or geometries.^24,37–43^ Bloch, Cook, and Luo show isomerization can influence what structure geometries are produced.^27,44,45^ Severin *et al.* reported a novel palladium-based coordination cage containing two dipyridyl ligand species.^46^ Importantly, Choe *et al.* shows a heteroleptic synthesis with linear terephthalic acid (1,4-bdc) ligands in zirconium cages produce a statistical distribution of heteroleptic cages utilizing mass spectrometry.^47^ They also found that mixed ligand speciation for linear ligands can have an effect on cage properties.^47^ Previous studies by Crowley and Craig have shown that mixed-ligand supramolecular cages can display a wide range of useful properties, such as increased kinetic stability.^48–50^ Supramolecular heteroleptic cages have been studied extensively, resulting in a variety of novel properties and applications.^51–55^ Additionally, the use of sterically hindered ligands to form heteroleptic supramolecular systems have been previously utilized.^40,55–58^ While mixed-ligand syntheses are not uncommon for metal–organic materials, no heteroleptic isophthalic acid (1,3-bdc) zirconium-based cages have been reported to our knowledge using this strategy. Herein, we report a novel zirconium structure and show how ligand ratio can be controlled within heteroleptic PCC systems using ligand bulk.

**Fig. 2:**
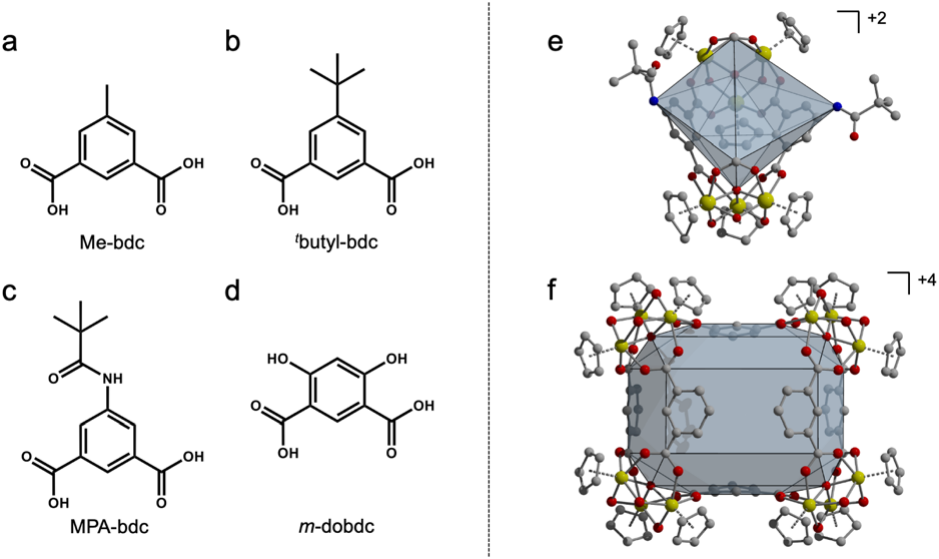
Ligands used in heteroleptic synthesis with 1,3-bdc (a–d). Homoleptic zirconium-based PCC geometry for bent 1,3-bdc ligands (e and f).

In this work, we focus on a series of mixed ligand zirconocene-capped cages of the general formula [Zr_12_(μ_3_-O)_4_(μ_2_-OH)_12_(Cp)_12_(L^1^)_*n*_(L^2^)_*m*_]Cl_4_ (*n* + *m* = 6; Cp = cyclopentadienyl; L = functionalized 5-R-isophthalic acid ligands). When bent dicarboxylate ligands are used, geometric isomers are formed that adopt open structures (Fig. 2e and f). These bent dicarboxylate ligands allow for greater access to active functional groups and increased ligand bulk. Here, we discuss heteroleptic syntheses including the depicted ligands (Fig. 2a–d). Structures of the cages reported here where larger functional groups in the 5-position of 1,3-bdc afford the Zr_6_L_2_ geometry (Fig. 2e) whereas smaller unfunctionalized ligands would typically form in the Zr_12_L_6_ geometry (Fig. 2f). We show that functional groups that do not otherwise lead to the formation of the desired cage structures can be used when they are mixed with ligands featuring smaller functional groups. Given the charge and solution stability of these cages, we further show that mass spectrometry can be used to quantify the extent of ligand mixing in product phases where, depending on the nature of the functional group, varying L^1^ : L^2^ proportions are seen. Due to the bulk and steric hindrance of the ligands used, the distribution of ligand ratios is shifted from the expected a Gaussian statistical distribution seen with 1,4-bdc ligands.^47^

## Results and discussion

**Fig. 3:**
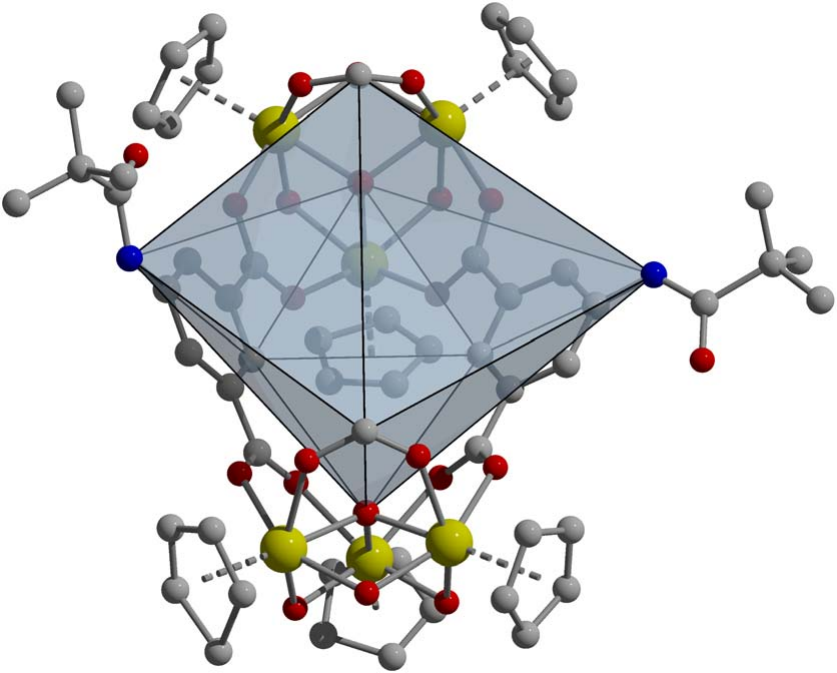
Novel boat-shaped Zr_6_L_2_ cage structure ([Zr_6_(μ_3_-O)_2_(μ_2_-OH)_6_(Cp)_6_(5-MPA)_2_(COOH)_2_]Cl_2_) containing two formate groups and two 5-MPA ligands. While the coordination geometry is not novel, the presence of two formate groups in place of where a third ligand would typically go, as with the lantern Zr_6_L_3_ geometry, is novel. This is likely due to the bulk of the ligand used, which illustrates how ligand geometry affects the resulting cage geometry.

In targeting functionalized zirconium-based cages, we chose 1,3-bdc ligands since structures based on these were expected to be more amenable to bulkier functionalization than the tetrahedral structure based on linear ligands, as the 5-positions of these are relatively unhindered in the basket-like structures. As we have shown that amide functionalization is a facile method for producing targeted ligands, we began with 5-(2,2-dimethylpropanoamido)-1,3-bdc (5-MPA).^1,15,59^ We have previously shown that the *tert*-butyl groups on the periphery of this ligand can endow copper-based cages with high thermal stability as a result of strong, directional ligand–ligand interactions.^1,59^ However, rather than the expected basket-like structure shown in Fig. 1, the reaction of zirconocene dichloride with 5-MPA in amide solvent affords a novel zirconium cage geometry with a formula of Zr_6_L_2_ (Fig. 3). In this system, the vertex : ligand ratio departs from the typical 2 : 3 ratio typically seen for these cages and is instead 1 : 1. In this particular structure, two formate groups that are formed during the reaction from the solvothermal decomposition of DMF cap each zirconium cluster where a third ligand would typically occupy. This cage features an open face that is approximately square with a length of ∼10 Å between the formate groups and width between the amide nitrogen atoms of ∼9 Å. Regardless of synthetic conditions, we were unable to produce the Zr_12_L_6_ structure that was expected for 1,3-bdc-based ligands.

As it would appear the 5-MPA ligand is too bulky to be successfully incorporated into a basket-shaped homoleptic cage, we turned to mixed-ligand cages. Synthesis of the unfunctionalized, homoleptic zirconium-based cage, [Zr_12_(μ_3_-O)_4_(μ_2_-OH)_12_(Cp)_12_(1,3-bdc)_6_]Cl_4_, is relatively straightforward. It is produced in high yield from a solvothermal, low-temperature reaction of ZrCp_2_Cl_2_ and 1,3-bdc in *N*,*N*-dimethylformamide (DMF) with a small amount of added water. As the structure consists of four metal caps and six organic ligands (Zr_12_L_6_), the reaction is run in a slight excess of ligand equivalents (∼1 : 0.6 L : M) where the equivalents of ligand are split between two species in different ratios. It was expected that partial substitution of a functionalized ligand for 1,3-bdc in the reaction mixture would lead to formation of a mixed-ligand cage.

**Fig. 4:**
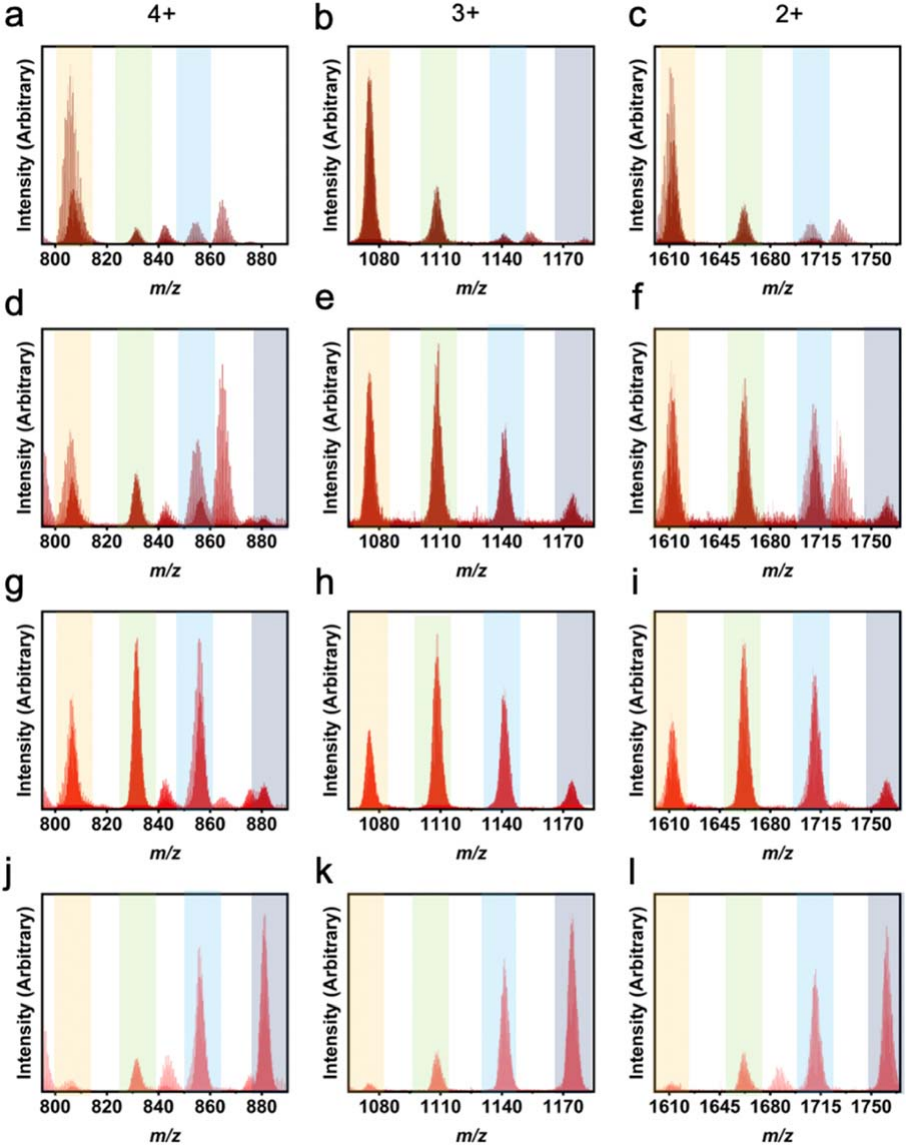
LC-MS results for heteroleptic 1,3-bdc : 5-MPA cages showing L^1^ : L^2^ ratios of 5 : 1 (a–c), 4 : 2 (d–f), 3 : 3 (g–i), and 2 : 4 (j–l). Column 1 depicts the 4+ *m*/*z* region, column 2 depicts the 3+ *m*/*z* region, and column 3 depicts the 2+ *m*/*z* region. While the highest peak in each plot indicates the true ligand ratio for the molecular cages, the other ratios can also be seen. Orange columns show peaks for 5 : 1, green for 4 : 2, blue for 3 : 3, and grey for 2 : 4.

Utilization of 5-MPA that otherwise affords the Zr_6_L_2_ structure, in a mixed-ligand synthesis showcases the versatility of the heteroleptic approach. Reaction of 1,3-bdc with 5-MPA in various ratios in amide solvent produces mixed ligand PCCs with compositions ranging from 5 : 1 to 2 : 4 (Fig. 4). Reactions containing higher ratios of 5-MPA did not afford cages with higher than 2 : 4 1,3-bdc : 5-MPA as the amide functional group on this ligand is not compatible with the basket-like structure. Additionally, there is an uneven distribution of ratios showing there is some structural inability for these bulky ligands to be included in these cages in higher ratios.^47^ For example, as shown in Fig. 4e, the most prevalent product is the 4 : 2 1,3-bdc : 5-MPA ratio. However, instead of an even presence of the 5 : 1 and 3 : 3 ratios, we instead see a higher 5 : 1 presence than 3 : 3. This shifted distribution can be attributed to the bulk of the ligand. While both NMR and liquid chromatography-mass spectrometry (LC-MS) clearly show this material to be cage, isotherms were also taken to confirm porosity. While nonporous to N_2_ at 77 K, the CO_2_ 195 K BET surface area of the 3 : 3 cage is 214 m^2^ g^−1^ (Fig. S5 and S6†), illustrating this material's permanent porosity. The difference in porosity between the two gases is likely due to the small size of CO_2_ as compared to N_2_.

Using solvothermal synthesis conditions, LC-MS and NMR indicate heteroleptic cages containing ratios of 1,3-bdc and the targeted functionalized ligands (5-MPA, 5-methyl, 5-NH_2_, 5-OH, 5-CN, *m*-dobdc, 5-NO_2_, 5-*tert*-butyl, 5-Br, and 5-benzyloxy) are formed in high yield. Depending on the ratio of ligands in the reaction mixture, varying levels of ligand incorporation are observed in product phase. These are generally found in a distribution where the propensity of ligand to incorporate into the structure at a given molar ratio varies between functional groups. Instead of a linear input : output ratio, there instead is statistical shifting that occurs in relation to the bulk of the ligand. ^1^H NMR is particularly useful to determine the ratio of L^1^ : L^2^ in the product phases as these cages are diamagnetic and spectra can be collected on crude samples. Alternatively, they can be digested for more detailed analysis as the size of undigested cage typically results in broad NMR resonances (Fig. S9–S46†). As shown in Fig. S47,† the ratio of ligands in the product phase typically follows those in the starting conditions for 1,3-bdc : functionalized ligand ratios of 4 : 2 or 3 : 3. For both 5-methyl and 5-*tert*-butyl, there is a preference for the incorporation of unfunctionalized ligand at higher ratios. Where a 1,3-bdc : 5-methyl or 1,3-bdc : 5-*tert*-butyl ratio of 2 : 4 affords bulk products with the composition of 2.47 : 3.53 and 3.22 : 2.78, respectively. This analysis, however, is representative of bulk solid and cannot discern between a mixture of phase-pure cages, phase-pure cage of a specific composition, or a combination of the two. In order to confirm the phase purity and amorphicity of these materials, powder X-ray diffraction (PXRD) patterns were obtained for homoleptic and heteroleptic cages (Fig. S48†).^60^ To more specifically interrogate the distribution of products in isolated solids, we turned to LC-MS. This technique is a valuable method in the characterization of zirconium cages as they are solution stable, tolerate the ionization methods, and have variable charge depending on composition.

In using LC-MS, integrating a single peak in each mass spectrometry chromatogram allows for observation of the expected *m*/*z* ratios that correspond to the 4+, 3+, and 2+ Zr_12_L_6_ cage ions. In this analysis, the 4+ ion is consistent with the loss of all four chloride counter ions while the 3+ and 2+ ions result from a loss of all four chloride counterions as well as one and two protons from the bridging μ-OH groups, respectively. It is important to distinguish between a mixture of homoleptic cages and the presence of a heteroleptic cage. Previously, homoleptic cage synthesis has resulted in mixed phase products, where the chromatogram produces two distinct peaks, which integrate individually to produce *m*/*z* peaks corresponding to two different geometries.^27^ In Zr_6_L_2_ and Zr_6_L_3_ geometries, there is a *m*/*z* peak corresponding to both 2+ and 1+ within the spectra, which would be found in the same region as the 4+ and 2+ of the Zr_12_L_6_ geometries. The difference between these geometries can be summarized by a missing 3+ peak for Zr_6_L_2_ and Zr_6_L_3_ systems. While the chromatograms for the heteroleptic molecular cages discussed here consistently produced one peak, these results indicate the presence of a mixture of heteroleptic cages where the most abundant species was controlled by changing the amount of ligand added in the one-pot synthesis.

**Fig. 5:**
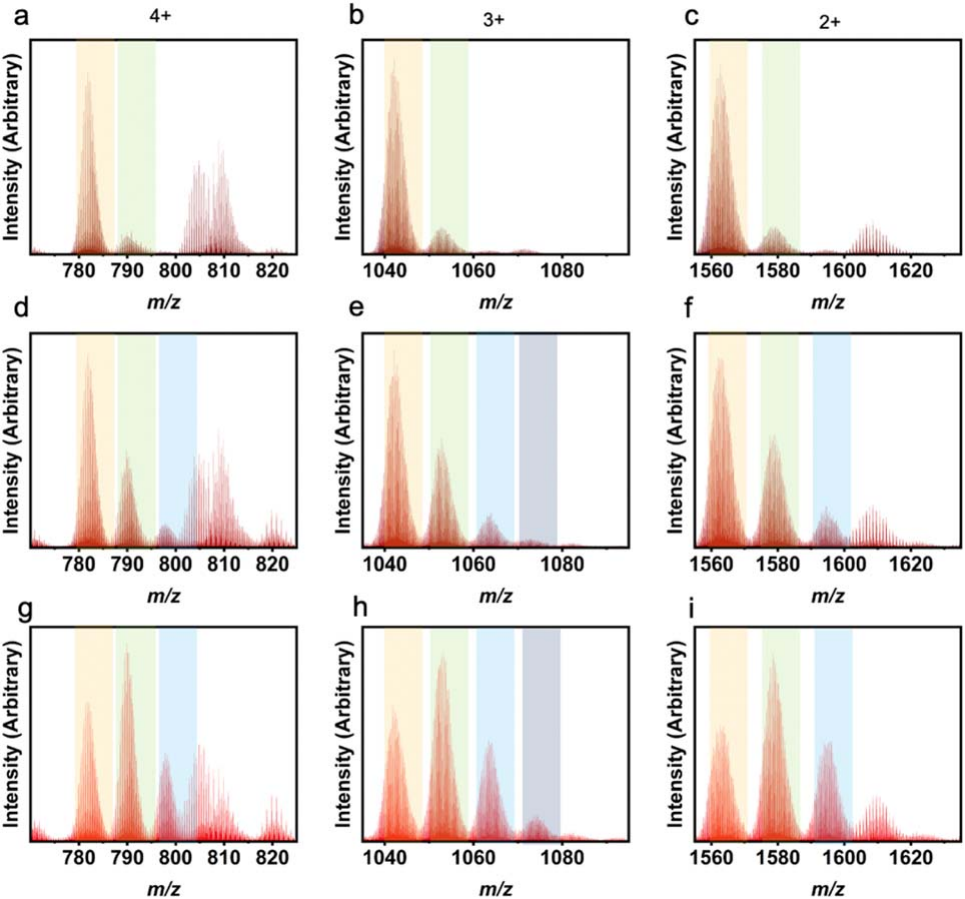
LC-MS results for heteroleptic 1,3-bdc : *m*-dobdc cages showing L^1^ : L^2^ ratios of 6 : 0 (a–f) and 5 : 1 (g–i) experimentally. Column 1 depicts the 4+ *m*/*z* region, column 2 the 3+ *m*/*z* region, and column 3 the 2+ *m*/*z* region. While the highest peak in each plot indicates the actual ligand ratio for the molecular cages, the theoretical ratios were 5 : 1 (a–c), 4 : 2 (d–f), and 3 : 3 (g–i) and can still be seen in these spectra. Orange columns show peaks for 6 : 0, green for 5 : 1, blue for 4 : 2, and grey for 3 : 3.

The LC-MS peaks have unique features associated with these heteroleptic cages, particularly for ligand pairs with significantly different masses. Each individual peak or shoulder corresponds to a different L^1^ : L^2^ ratio within a given experiment. Previously, *m*-dobdc has not been successfully implemented into a zirconium-based PCC. By utilizing a heteroleptic synthesis, *m*-dobdc is now able to be incorporated into these systems. Through the use of LC-MS, we see a distribution of cages containing 1,3-bdc : *m*-dobdc ratios from 6 : 0 to 3 : 3 (Fig. 5). While in other work, there is an even distribution of heteroleptic cage ligand ratios, here we see a shifted distribution.^47^ Illustrative of the difficulty in isolating cages containing *m*-dobdc, 1,3-bdc preferentially incorporates even at low 1,3-bdc : *m*-dobdc ratios. As seen in Table S5† and Fig. 5, with a 4 : 2 ratio, a homoleptic 1,3-bdc cage is still the predominate product. However, with a 3 : 3 input, we observe the presence of the 5 : 1 cage most clearly. Therefore, *m*-dobdc must be in large excess of 1,3-bdc to be incorporated into zirconium-based molecular cages. While *m*-dobdc has evaded inclusion in zirconium PCCs previously, it can now be incorporated into these materials with the utilization of a heteroleptic cage synthesis.

Given the bulk of the functional group on 5-*tert*-butyl, it is expected that a homoleptic cage would adopt the Zr_6_L_2_ structure. Instead, by utilizing a mixed ligand synthesis to give a heteroleptic structure, it can be incorporated into a larger Zr_12_L_6_ structure. By opting for a ratio including both this bulky ligand and 1,3-bdc, a Zr_12_L_6_ structure containing 5-*tert*-butyl can isolated. Similar to the results seen for the methyl-functionalized ligand, integration of the singular chromatogram peak gives spectra consistent with a molecular cage for L^1^ : L^2^ ratios of 4 : 2 through 2 : 4, Table S3.† As the mass differences between the two incorporated ligands are more significant for this pair, the peak patterns in the chromatograms (Fig. S49†) are striking. For 4 : 2, 3 : 3, and 2 : 4 1,3-bdc : 5-*tert*-butyl ratios, the most intense peak corresponds to cage with L : L composition of 4 : 2, 3 : 3, and 2 : 4, respectively, regardless of the mass ion that is analyzed. For this system, additional *m*/*z* peaks are present that have thus far evaded identification, although they appear in the 4+ and 2+ regions of all experimental ratios and absent in the 3+ region, which indicates the product phase is likely a two-vertex product.

**Fig. 6:**
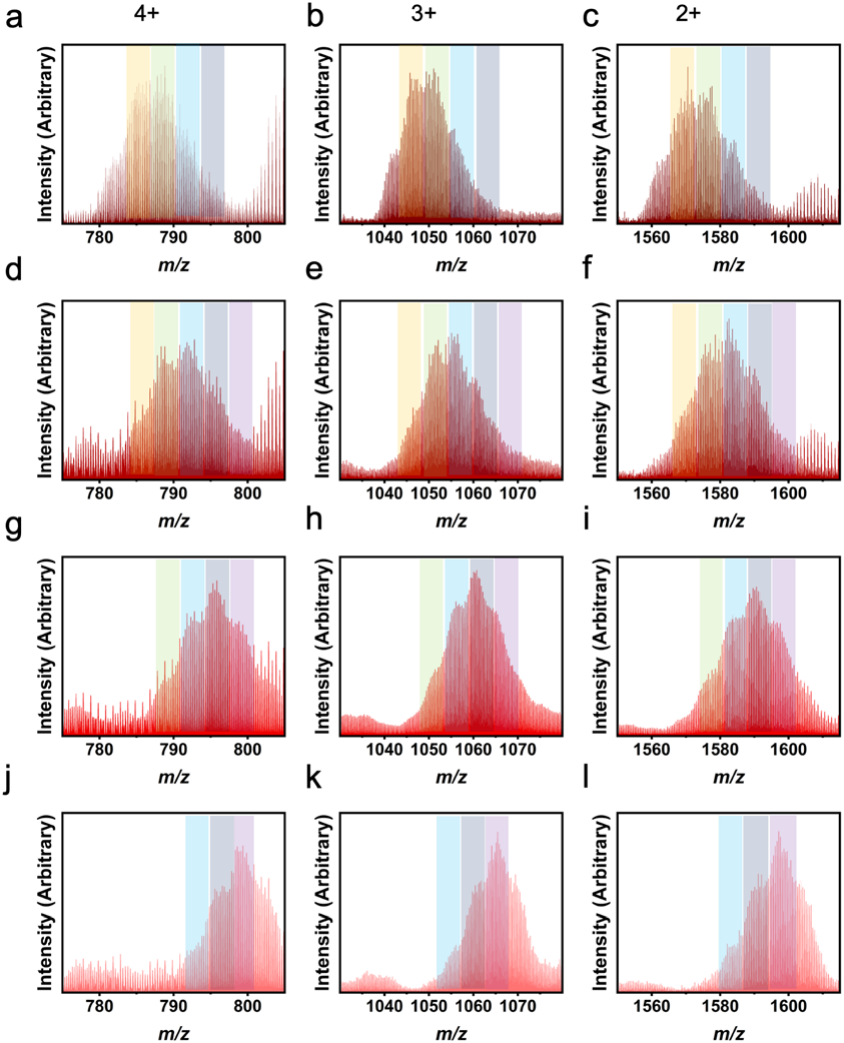
LC-MS results for heteroleptic 1,3-bdc : 5-methyl cages showing L^1^ : L^2^ ratios of 4 : 2 (a–c), 3 : 3 (d–f), 2 : 4 (g–i), 1 : 5 (j–l). Column 1 depicts the 4+ *m*/*z* region, column 2 depicts the 3+ *m*/*z* region, and column 3 depicts the 2+ *m*/*z* region. While the highest peak in each plot indicates the expected ligand ratio for the molecular cages, the other ratios can also be seen. Orange columns show peaks for 5 : 1, green for 4 : 2, blue for 3 : 3, indigo for 2 : 4, and purple for 1 : 5. The 6 : 0 and 0 : 6 peaks can also be seen in row 1 and 2, respectively.

Not only are ligand ratios of vastly different molecular weights in heteroleptic systems elucidated *via* LC-MS, but very small changes in molecular weight between L^1^ and L^2^ can be seen clearly with this characterization method. For a homoleptic 1,3-bdc cage, the *m*/*z* ratios expected are 782 (4+), 1042 (3+), and 1563 (2+). For a homoleptic 5-methyl cage, the *m*/*z* ratios expected are 803 (4+), 1070 (3+), and 1650 (2+) (Fig. S50†). A cage containing a ligand ratio of 5 : 1 (1,3-bdc : 5-methyl), would produce *m*/*z* peaks at 785 (4+), 1047 (3+), and 1570 (2+) (Fig. 6). Therefore, the ligand ratio observed in any given cage could be accurately determined *via* high-resolution LC-MS. Upon analyzing mass spectrometry data for these cages, a ligand ratio of 3 : 3 (1,3-bdc : 5-methyl) produced a product with a single peak on the chromatogram which, when integrated, afforded broad *m*/*z* peaks at 793 (4+), 1056 (3+), and 1585 (2+). These peaks match the expected *m*/*z* peaks. In addition, a ligand ratio of 2 : 4 (1,3-bdc : 5-methyl) produced a single peak on the chromatogram which, when integrated, resulted in *m*/*z* peaks at 796 (4+), 1061 (3+), and 1591 (2+). This is consistent with the expected *m*/*z* peaks at 796 (4+), 1061 (3+), and 1591 (2+), which match experimental peaks. A summary of the theoretical and experimental *m*/*z* values for all the 1,3-bdc : 5-methyl mixed ligand cage ratios are depicted in Table S2.†

With a better understanding of the product distributions that can be expected for mixed-ligand cages based on isophthalic acid ligands, we further explored combinations with 11 other 1,3-bdc-based ligands. Based off the original four samples discussed within this manuscript, we were interested in determining how incorporating two different functionalized ligands affects cage geometry. Through the synthesis of cages containing two types of active functionality, we increase the tunability and applications of these systems.

Based on ^1^H NMR and LC-MS, we observed a wide range of heteroleptic systems containing 3 : 3 ratios of different combinations of 1,3-bdc, 5-MPA, 5-methyl, 5-NH_2_, 5-OH, 5-CN, *m*-dobdc, 5-NO_2_, 5-*tert*-butyl, 5-Br, and 5-benzyloxy.

LC-MS spectra were analyzed using a direct injection method and, for consistency, spectra were taken at elution times of 2.201 min (±0.016 min) (Fig. S51–S77†). However, different ratios appear to elute at different times, suggesting the ability of these molecular cage products to be separated through further analytical methods (Fig. S78†). While the input for each combination of ligands was 3 : 3, a slight preference for one ligand or the other is observed, as seen with *m*-dobdc (Table 1). NMR and LC-MS ratios for each sample do not align for every sample, which is reasonable considering NMR analyzes the average of the sample, while LC-MS looks at the mode distribution.

**Table 1:** Ligand ratios from NMR and LC-MS

Ligand A	Ligand B	NMR A : B	MS A : B
1,3-bdc	5-CN	2.90 : 3.10	4 : 2
1,3-bdc	5-Br	3.48 : 2.52	4 : 2
1,3-bdc	5-NO_2_	3.67 : 2.33	4 : 2
5-Methyl	*m*-dobdc	4.71 : 1.29	5 : 1
5-Methyl	5-CN	2.80 : 3.20	4 : 2
5-Methyl	5-Br	3.61 : 2.39	4 : 2
5-CN	*m*-dobdc	4.82 : 1.18	5 : 1
5-NH_2_	5-NO_2_	1.97 : 4.03	3 : 3
5-CN	5-Br	3.62 : 2.38	2 : 4
5-CN	5-NO_2_	3.45 : 2.55	2 : 4

**Fig. 7:**
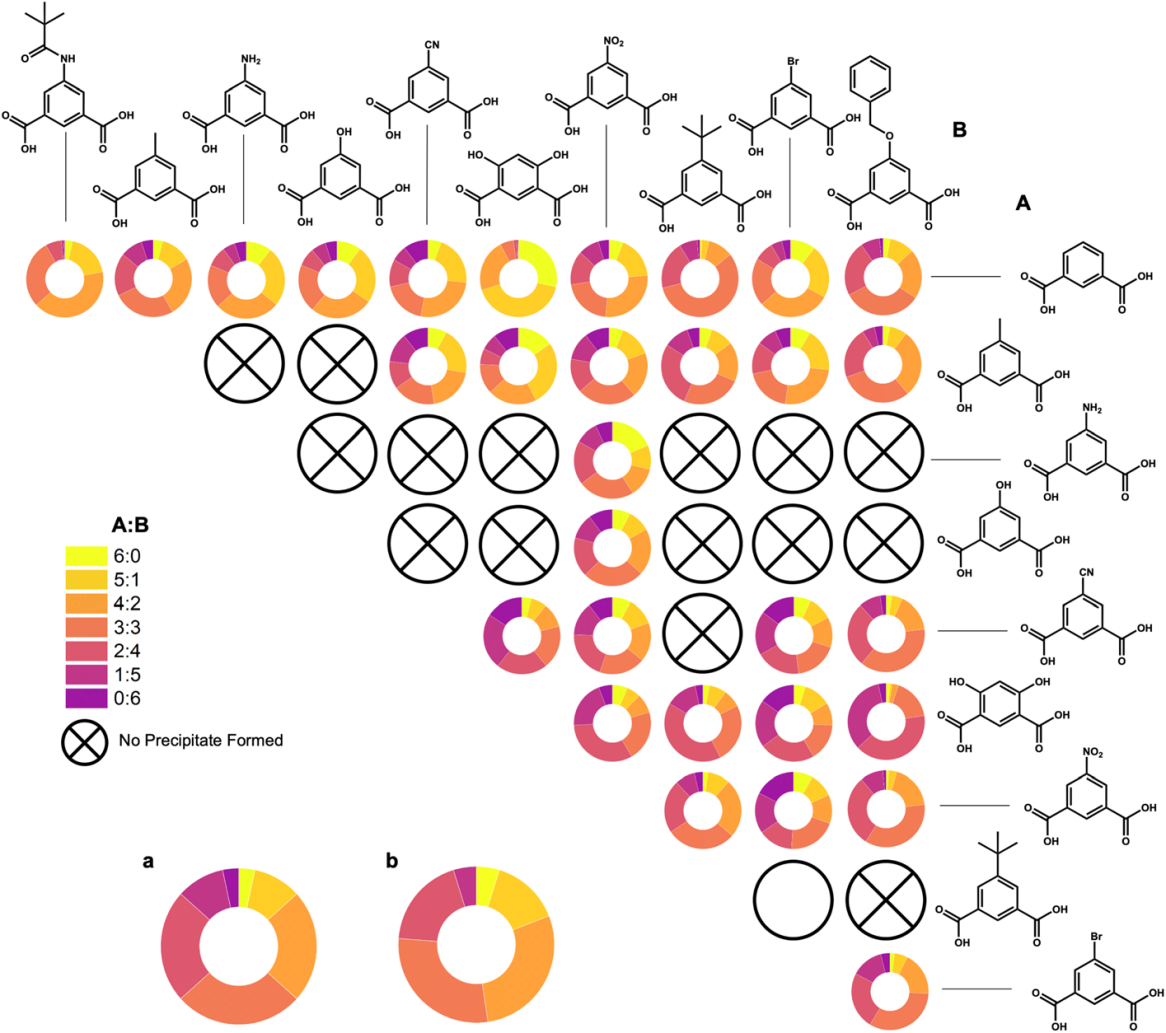
Percentages of all A : B ligand ratios (6 : 0 to 0 : 6) from LC-MS spectra, indicating which combinations yielded heteroleptic systems and one case which was inconclusive (5-*tert*-butyl : 5-Br), denoted by an open circle. Percentages of expected distributions of A : B ratios (a) and percentages of shifted distributions of A : B where B is a much bulkier ligand (b) are also included.

By taking the most intense peak in the spectra that is ±0.5*m*/*z* away from the predicted *m*/*z*, we have obtained percentages of ratios produced, from 6 : 0 to 0 : 6, for all combinations synthesized (Fig. 7). In addition to these basket-shaped Zr_12_L_6_ cages, the boat-shaped Zr_6_L_2_ cages are also present in the most of these LC-MS spectra (Fig. S79–S81†).

Expected distributions for an input of two bent ligand species in a 3 : 3 ratio are 1 : 3 : 7 : 8 : 7 : 3 : 1 (6 : 0 through 0 : 6). This matches with ligand species of similar molecular weights, for example, 1,3-bdc and 5-methyl. However, we see larger, bulkier ligand species depart from this expectation. This is reasonable considering in a basket-like structure there are two ligands that point towards each other. One can logically assume ligand–ligand interactions between bulkier ligands would prevent these ligands from forming homoleptic cages. In addition, in heteroleptic systems we can assume there is the same issue if a number greater than one of these bulky ligand species was incorporated into these heteroleptic systems where two of these ligands point toward each other. Therefore, for bulkier ligands, a shifted distribution is expected where we see the L^1^ : L^2^ cage ratios from 6 : 0 to 0 : 6 (where L^2^ is the bulkier ligand) is expected to be 1 : 3 : 6 : 6 : 4 : 1 : 0. For example, 1,3-bdc : 5-MPA shows this distribution quite similarly where little to no homoleptic cages are present, favoring the less bulky of the two species, 1,3-bdc. Similarly to initial experimentation, heteroleptic systems including *m*-dobdc preferentially select the secondary ligand in the system, creating a shifted distribution to favor this second ligand over *m*-dobdc.

It is important to also determine if this heteroleptic cage formation occurs during solvothermal synthesis, or if this is the product of ligand exchange from a homoleptic cage to a heteroleptic cage. To ensure this is truly heteroleptic cage formation, homoleptic 1,3-bdc cage was stirred with an excess of each of the four initial ligands used: 5-methyl, 5-MPA, 5-*tert*-butyl, and *m*-dobdc. Based off the LC-MS of the solid from these experiments (Fig. S82–S85†), the 1,3-bdc cage was the only cage present in the spectra. Therefore, it can be concluded that these ligands do not exchange under these conditions, which is in general agreement with the previous work reported by Choe and demonstrates the structural stability of these cages.^47^

Heteroleptic cages of 1,3-bdc with 5-methyl, 5-*tert*-butyl, *m*-dobdc, and 5-MPA ligands display high thermal stability for zirconium-based cages under both N_2_ and O_2_ (Fig. S86–S93†). Additionally, this method of cage synthesis was utilized in a click reaction where propargyl alcohol was clicked onto a molecular zirconium cage with a 4 : 2 ratio of 1,3-bdc : 5-N_3_. Based on IR, NMR, and LC-MS of the 1,3-bdc : 5-N_3_ cage and the clicked cage, it is clear that this method helps in increasing the number of clicked substituents (Fig. S94–S99†). Based on the percentages of these fully clicked ligand ratios, there is a clear preference for the 5 : 1 and 4 : 2 (1,3-bdc : 5-N_3_-PrOH) fully clicked cages, with a decrease in the presence of 2 : 4, 1 : 5, and 0 : 6 ratios (Fig. S97†). These ratios are still present and we expect them to be able to be isolated based on an increased intensity at different elution times in the LC-MS.

As mixed ligand syntheses can be used to tune cage structure, it can also be used to prepare cages containing groups that are otherwise incompatible with cage formation. For example, while the ligands used in other heteroleptic cages within this manuscript have previously formed molecular cages of varying geometry and nuclearity, *m*-dobdc has not been incorporated into zirconium cage structures. As *m*-dobdc has the same carboxylate–carboxylate angle as in 1,3-bdc, it would be expected it to form in either the Zr_12_L_6_ or the Zr_6_L_2_ geometries. When comparing these structures, it is important to note that in the Zr_12_L_6_ system, two ligands point toward each other in one orientation and, on the opposite face, the other four ligands point up. The issue with incorporating *m*-dobdc in this system stems from the side in which the two point toward each other due to ligand–ligand interactions. In the Zr_6_L_2_ system, the ligands only point up, however, the issue still lies in the accessibility of the hydroxide moieties. Therefore, to successfully incorporate *m*-dobdc in a zirconium cage structure, it is beneficial to employ a heteroleptic synthesis in which an unfunctionalized ligand, 1,3-bdc, can facilitate cage formation. In addition to these 1,3-bdc : functionalized heteroleptic cages, we have also synthesized cages containing two ligands containing different active functionality. These facile one-pot syntheses fill a critical need to increase tunability of these systems by allowing for ligand species that have been challenging to incorporate into these systems successfully form cage structures and incorporating two types of active functionality into one system.

## Conclusions

In conclusion, this work demonstrates potential to effectively modulate specific properties within zirconium-based PCCs through the strategic incorporation of functionality. While some ligands pose challenges for integration due to steric effects, our approach employing a facile heteroleptic one-pot synthesis has proven instrumental in achieving controlled ratios of cages containing at least two distinct species of ligands. Notably, we have successfully designed a straightforward heteroleptic zirconium cage synthesis, overcoming previous limitations associated with ligands that either adopted a mildly porous structure or resisted successful implementation in zirconium cages. The application of this method not only expands the scope of ligands that can be integrated into porous structures but also provides valuable insights into using functionality to finely tune the properties of these molecular cages. By successfully incorporating ligands hosting two different functional groups, we have created more tunable systems that have overcome the challenge with incorporating bulky active functionality into zirconium-based porous coordination cages. Our findings contribute to advancing the understanding of the relationship between ligand design and cage properties, which will allow for tailored development of zirconium-based porous materials for diverse applications.

## Author contributions

MNM preformed data acquisition and analysis. CMM and MNM developed LC-MS methodology. MRD and GPAY acquired and modelled SC-XRD data. EDB and MNM wrote the manuscript. All authors reviewed and edited the manuscript. EDB acquired funding and supervised the project.

## Conflicts of interest

There are no conflicts to declare.

## Supplementary Material

SC-016-D4SC06023G-s001

SC-016-D4SC06023G-s002

## Data Availability

The data supporting this article have been included as part of the ESI.† Crystallographic data is uploaded with the manuscript and has been deposited in the CCDC: 2321239.
