# Supplementary material for: Tunable synthesis of heteroleptic zirconium-based porous coordination cages
Source: Chem Sci. 2024 Nov 18;16(2):816–23. doi: 10.1039/d4sc06023g (PMC11616777; doi:10.1039/d4sc06023g)
Supplement: SC-016-D4SC06023G-s001 [file SC-016-D4SC06023G-s001.pdf]

Supplementary Information (SI) for Chemical Science.
This journal is © The Royal Society of Chemistry 2024

Electronic Supplementary Information

**Tunable Synthesis of Heteroleptic Zirconium-Based Porous Coordination Cages**

Merissa N. Morey[1], Christine M. Montone[1], Michael R. Dworzak[2], Glenn P.A Yap[2], Eric D. Bloch[1*]

[1] Department of Chemistry, Indiana University, Bloomington, IN 47405, USA

[2]Department of Chemistry & Biochemistry, University of Delaware, Newark, DE 19716, USA

*email: edbloch@iu.edu

Table of Contents

**Table S1. Ligand Structures and Abbreviations** .... 3
**Detailed Experimental Procedures** .... 4
Materials and Methods .... 4
Ligand Synthesis .... 4
Homoleptic Cage Synthesis .... 4
Stock Solutions for Heteroleptic Cage Synthesis .... 5
Heteroleptic Cage Synthesis .... 6
Control Experiments .... 12
Click Reaction .... 13
**Gas Adsorption Measurements** .... 14
**$^1$H-NMR Spectra** .... 18
**Powder X-Ray Diffraction (PXRD)** .... 37
**Liquid Chromatography- Mass Spectrometry (LC-MS)** .... 38
Example Calculations for LC-MS M/Z Peaks .... 39
**Detailed Single Crystal X-Ray Diffraction Information and Crystallographic Information** .... 51
**Thermogravimetric Analysis (TGA)** .... 52
**Click Reaction** .... 56
Infrared Spectroscopy (IR) .... 56
LC-MS .... 57
$^1$H NMR .... 58
**References** .... 59

## Table S1. Ligand Structures and Abbreviations

| Ligand Structure | Ligand Abbreviation |
|---|---|
| 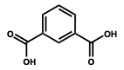 | 1,3-bdc |
| 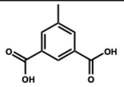 | 5-methyl |
| 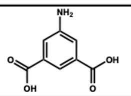 | 5-$NH_2$ |
| 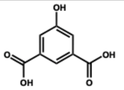 | 5-OH |
| 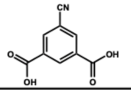 | 5-CN |
| 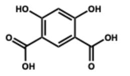 | *m*-dobdc |
| 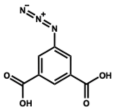 | 5-$N_3$ |

| Ligand Structure | Ligand Abbreviation |
|---|---|
| 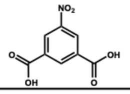 | 5-$NO_2$ |
| 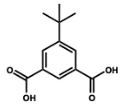 | 5-*tert*-butyl |
| 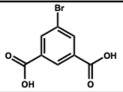 | 5-Br |
| 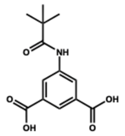 | 5-MPA |
| 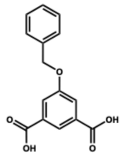 | 5-benzyloxy |
| 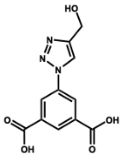 | 5-$N_3$-PrOH |

## Detailed Experimental Procedures

Materials and Methods

All reagents were obtained from commercial vendors and used without purification in air unless otherwise specified.

ESI-LC-MS experiments were run on a Waters Synapt G2S HDMS system equipped with a C18 column and a high-resolution single quadrupole detector with a 5 min LC-MS data collection program. Roughly 0.3 mg of cage material was dissolved in 4 mL MeOH, unless otherwise noted. Data was analyzed using MassLynx software.[1] Experiments using stock solutions 1-11 were carried out on the same instrument using a direct-injection loop bypass method.

Thermogravimetric Analysis (TGA) measurements were carried out from room temperature to 600 °C at a 2 °C $min^{-1}$ heating rate with a TA Q5000 SA under a nitrogen flow. Combustion TGA were also collected under oxygen flow from room temperature to 600 °C at a 2 °C $min^{-1}$ heating rate.

$^1$H-NMR spectra were obtained using a Bruker 500 MHz spectrometer and data obtained was processed in MestReNova NMR processor software. Cage acid digestions were performed by adding one drop of 35 wt. % DCl in $D_2O$ and 0.5 mL of DMSO-$d_6$ or MeOH-$d_4$, unless otherwise noted, to a 4 mL vial containing roughly 10 mg of cage and sonicated until homogenous.

Powder X-Ray Diffraction (PXRD) was carried out utilizing an Empyrean multipurpose diffractometer from PANalytical with a Cu radiation source. Data collection occured at room temperature, with a 15 mm mask, 0.5 ° slit, and step width of 5.00 °.

Infrared Spectroscopy (IR) was carried out using a Bruker Alpha II instrument using a KBr pellet method to collect data.

Ligand Synthesis

*5-(MPA)-1,3-bdc*

5-Aminoisophthalic acid (2.000 g, 11 mmol) and *tert*-butylacetal chloride (2.179 mL, 22 mmol) were added to a 250 mL RBF along with THF (100 mL) and stirred under reflux at 80˚C for 24 hours. Solvent was removed under vacuum, leaving a yellow solid behind. $CHCl_3$ (30 mL) was added to the crude product, stirred until powder was fully dispersed. Solvent was removed via Büchner funnel under reduced pressure. Two more washings with $CHCl_3$ were performed. Solvent was removed under reduced pressure using a Büchner funnel and light yellow solid was collected.

Homoleptic Cage Synthesis

*$[Zr_{12}(\mu_3\text{-}O)_4(\mu_2\text{-}OH)_{12}(Cp)_{12}(1,3\text{-}bdc)_6]Cl_4$*

In a 20 mL vial zirconocene dichloride (672 mg, 2.3 mmol) and isophthalic acid (191 mg, 1.15 mmol) were dissolved in DMF (19 mL). The stock solution (2.375 mL) and DI water (77 µL) were transferred to 9 individual 4 mL vials and heated at 65 °C for 8 hours. White solid was collected via centrifugation followed by decanting the mother liquor. The material was then washed 3x with DMF followed by 3 washes with $CHCl_3$ with solvent exchanged every 12 hours.

*$[Zr_{12}(\mu_3\text{-}O)_4(\mu_2\text{-}OH)_{12}(Cp)_{12}(5\text{-methyl bdc})_6]Cl_4$*

In a 20 mL vial zirconocene dichloride (225 mg, 0.770 mmol) and 5-methylisophthalic acid (75 mg, 0.416 mmol) were dissolved in DMF (20 mL). The stock solution (5 mL) and DI water (750 µL) were transferred to 4 individual 20 mL vials and heated at 65 °C for 8 hours. White solid was collected via centrifugation followed by decanting the mother liquor. The material was then washed 3x with DMF followed by 3 washes with $CHCl_3$ with solvent exchanged every 12 hours.

*$[Zr_6(\mu_3\text{-}O)_2(\mu_2\text{-}OH)_6(Cp)_6(CO_2)_2(5\text{-(MPA)-1,3-bdc})_2]Cl_2$*

In a 20 mL vial zirconocene dichloride (113 mg, 0.383 mmol) and 5-(MPA) isophthalic acid (37 mg, 0.140 mmol) were dissolved in DMF (10 mL). The stock solution (1 mL) and DI water (150 µL) were transferred to 10 individual 4 mL vials and heated at 45 °C for 10 hours. White solid was collected via centrifugation followed by decanting the mother liquor. Solid was washed with DMF three times followed by three washes with $CHCl_3$ with solvent exchanged every 12 hours.

Stock Solutions for Heteroleptic Cage Synthesis

*Zirconocene dichloride solution (1)*

In a 100 mL VWR jar zirconocene dichloride (4.746 g, 16.2 mmol) was dissolved in DMF (84 mL). The solution was sonicated until clear.

*Bdc stock solution (2)*

In a 50 mL VWR jar isophthalic acid (115.3 mg, 0.694 mmol) was dissolved in DMF (24 mL). The solution was sonicated until clear.

*t-butyl stock solution (3)*

In a 50 mL VWR jar 5-*tert*-butylisophthalic acid (202.4 mg, 0.911 mmol) was dissolved in DMF (32 mL). The solution was sonicated until clear.

*5-me stock solution (4)*

In a 50 mL VWR jar 5-methylisophthalic acid (163.1 mg, 0.905 mmol) was dissolved in DMF (32 mL). The solution was sonicated until clear.

*m-dobdc stock solution (5)*

In a 50 mL VWR jar 4,6-dihydroxyisophthalic acid (*m*-dobdc)[2] (178.8 mg, 0.902 mmol) was dissolved in DMF (32 mL). The solution was sonicated until clear.

*Br stock solution (6)*

In a 50 mL VWR jar 5-bromoisophthalic acid (247.0 mg, 1.01 mmol) was dissolved in DMF (36 mL). The solution was sonicated until clear.

*OH stock solution (7)*

In a 50 mL VWR jar 5-hydroxyisophthalic acid (185.7 mg, 1.02 mmol) was dissolved in DMF (36 mL). The solution was sonicated until clear.

*$NH_2$ stock solution (8)*

In a 50 mL VWR jar 5-aminoisophthalic acid (184.1 mg, 1.02 mmol) was dissolved in DMF (36 mL). The solution was sonicated until clear.

*CN stock solution (9)*
In a 50 mL VWR jar 5-cyanoisophthalic acid (195.2 mg, 1.02 mmol) was dissolved in DMF (36 mL). The solution was sonicated until clear.
*Benzyloxy stock solution (10)*
In a 50 mL VWR jar 5-benzyloxyisophthalic acid[3] (277.2 mg, 1.02 mmol) was dissolved in DMF (36 mL). The solution was sonicated until clear.
*$NO_2$ stock solution (11)*
In a 50 mL VWR jar 5-nitroisophthalic acid (215.3 mg, 1.02 mmol) was dissolved in DMF (36 mL). The solution was sonicated until clear.

Heteroleptic Cage Synthesis
*$[Zr_{12}(\mu_3\text{-}O)_4(\mu_2\text{-}OH)_{12}(Cp)_{12}(5\text{-methyl bdc})_2(bdc)_4]Cl_4$*
In a 20 mL vial zirconocene dichloride (225 mg, 0.770 mmol), 5-methylisophthalic acid (27 mg, 0.150 mmol), and isophthalic acid (50 mg, 0.301 mmol) were dissolved in DMF (20 mL). The stock solution (1 mL) and DI water (150 μL) were transferred to 20 individual 4 mL vials and heated at 45 °C for 8 hours. White solid was collected via centrifugation followed by decanting the mother liquor.
*$[Zr_{12}(\mu_3\text{-}O)_4(\mu_2\text{-}OH)_{12}(Cp)_{12}(5\text{-methyl bdc})_3(bdc)_3]Cl_4$*
In a 20 mL vial zirconocene dichloride (225 mg, 0.770 mmol), 5-methylisophthalic acid (41 mg, 0.226 mmol), and isophthalic acid (38 mg, 0.226 mmol) were dissolved in DMF (20 mL). The stock solution (1 mL) and DI water (150 μL) were transferred to 20 individual 4 mL vials and heated at 45 °C for 8 hours. White solid was collected via centrifugation followed by decanting the mother liquor. Material was activated under $N_2$ flow at 40 °C for 16 hours.
*$[Zr_{12}(\mu_3\text{-}O)_4(\mu_2\text{-}OH)_{12}(Cp)_{12}(5\text{-methyl bdc})_4(bdc)_2]Cl_4$*
In a 20 mL vial zirconocene dichloride (225 mg, 0.770 mmol), 5-methylisophthalic acid (54 mg, 0.301 mmol), and isophthalic acid (25 mg, 0.150 mmol) were dissolved in DMF (20 mL). The stock solution (1 mL) and DI water (150 μL) were transferred to 20 individual 4 mL vials and heated at 45 °C for 8 hours. White solid was collected via centrifugation followed by decanting the mother liquor.
*$[Zr_{12}(\mu_3\text{-}O)_4(\mu_2\text{-}OH)_{12}(Cp)_{12}(5\text{-methyl bdc})_5(bdc)]Cl_4$*
In a 20 mL vial zirconocene dichloride (225 mg, 0.770 mmol), 5-methylisophthalic acid (68 mg, 0.375 mmol), and isophthalic acid (13 mg, 0.0752 mmol) were dissolved in DMF (20 mL). The stock solution (1 mL) and DI water (150 μL) were transferred to 20 individual 4 mL vials and heated at 45 °C for 8 hours. White solid was collected via centrifugation followed by decanting the mother liquor.
*$[Zr_{12}(\mu_3\text{-}O)_4(\mu_2\text{-}OH)_{12}(Cp)_{12}(m\text{-dobdc})(bdc)_5]Cl_4$*
In a 20 mL vial zirconocene dichloride (112.5 mg, 0.385 mmol), 4,6-dihydroxyisophthalic acid[2] (8 mg, 0.0376 mmol), and isophthalic acid (31 mg, 0.188 mmol) were dissolved in DMF (10 mL). The stock solution (1 mL) and DI water (150 μL) were transferred to 10

individual 4 mL vials and heated at 45 °C for 8.3 hours. White solid was collected via centrifugation followed by decanting the mother liquor.

*$[Zr_{12}(\mu_3\text{-}O)_4(\mu_2\text{-}OH)_{12}(Cp)_{12}(m\text{-}dobdc)_2(bdc)_4]Cl_4$*

In a 20 mL vial zirconocene dichloride (112.5 mg, 0.385 mmol), 4,6-dihydroxyisophthalic acid[2] (15 mg, 0.0750 mmol), and isophthalic acid (25 mg, 0.150 mmol) were dissolved in DMF (10 mL). The stock solution (1 mL) and DI water (150 µL) were transferred to 10 individual 4 mL vials and heated at 45 °C for 8.3 hours. White solid was collected via centrifugation followed by decanting the mother liquor.

*$[Zr_{12}(\mu_3\text{-}O)_4(\mu_2\text{-}OH)_{12}(Cp)_{12}(m\text{-}dobdc)_3(bdc)_3]Cl_4$*

In a 20 mL vial zirconocene dichloride (112.5 mg, 0.385 mmol), 4,6-dihydroxyisophthalic acid[2] (22 mg, 0.113 mmol), and isophthalic acid (19 mg, 0.113 mmol) were dissolved in DMF (10 mL). The stock solution (1 mL) and DI water (150 µL) were transferred to 10 individual 4 mL vials and heated at 45 °C for 8.3 hours. White solid was collected via centrifugation followed by decanting the mother liquor. Material was activated under $N_2$ flow at 40 °C for 16 hours.

*$[Zr_{12}(\mu_3\text{-}O)_4(\mu_2\text{-}OH)_{12}(Cp)_{12}(5\text{-}(MPA)bdc)(bdc)_5]Cl_4$*

In a 20 mL vial zirconocene dichloride (112.5 mg, 0.385 mmol), 5-(MPA) isophthalic acid (10 mg, 0.0376 mmol), and isophthalic acid (31 mg, 0.188 mmol) were dissolved in DMF (10 mL). The stock solution (1 mL) and DI water (150 µL) were transferred to 10 individual 4 mL vials and heated at 45 °C for 8.3 hours. White solid was collected via centrifugation followed by decanting the mother liquor.

*$[Zr_{12}(\mu_3\text{-}O)_4(\mu_2\text{-}OH)_{12}(Cp)_{12}(5\text{-}(MPA)bdc)_2(bdc)_4]Cl_4$*

In a 20 mL vial zirconocene dichloride (112.5 mg, 0.385 mmol), 5-(MPA) isophthalic acid (20 mg, 0.0750 mmol), and isophthalic acid (25 mg, 0.151 mmol) were dissolved in DMF (10 mL). The stock solution (1 mL) and DI water (150 µL) were transferred to 10 individual 4 mL vials and heated at 45 °C for 8.3 hours. White solid was collected via centrifugation followed by decanting the mother liquor.

*$[Zr_{12}(\mu_3\text{-}O)_4(\mu_2\text{-}OH)_{12}(Cp)_{12}(5\text{-}(MPA)bdc)_3(bdc)_3]Cl_4$*

In a 20 mL vial zirconocene dichloride (112.5 mg, 0.385 mmol), 5-(MPA) isophthalic acid (30 mg, 0.113 mmol), and isophthalic acid (19 mg, 0.113 mmol) were dissolved in DMF (10 mL). The stock solution (1 mL) and DI water (150 µL) were transferred to 10 individual 4 mL vials and heated at 45 °C for 8.3 hours. White solid was collected via centrifugation followed by decanting the mother liquor. Material was activated under $N_2$ flow at 40 °C for 16 hours.

*$[Zr_{12}(\mu_3\text{-}O)_4(\mu_2\text{-}OH)_{12}(Cp)_{12}(5\text{-}(MPA)bdc)_4(bdc)_2]Cl_4$*

In a 20 mL vial zirconocene dichloride (112.5 mg, 0.385 mmol), 5-(MPA) isophthalic acid (40 mg, 0.151 mmol), and isophthalic acid (12.5 mg, 0.0750 mmol) were dissolved in DMF (10 mL). The stock solution (1 mL) and DI water (150 µL) were transferred to 10 individual 4 mL vials and heated at 45 °C for 8.3 hours. White solid was collected via centrifugation followed by decanting the mother liquor.

*$[Zr_{12}(\mu_3\text{-}O)_4(\mu_2\text{-}OH)_{12}(Cp)_{12}(5\text{-}tert\text{-}butyl\ bdc)_2(bdc)_4]Cl_4$*

In a 20 mL vial zirconocene dichloride (112.5 mg, 0.385 mmol), 5-*tert*-butylisophthalic acid (16.5 mg, 0.0750 mmol), and isophthalic acid (25 mg, 0.151 mmol) were dissolved in DMF (10 mL). The stock solution (1 mL) and DI water (150 μL) were transferred to 10 individual 4 mL vials and heated at 45 °C for 8.3 hours. White solid was collected via centrifugation followed by decanting the mother liquor.

*$[Zr_{12}(\mu_3\text{-}O)_4(\mu_2\text{-}OH)_{12}(Cp)_{12}(5\text{-}tert\text{-}butyl\ bdc)_3(bdc)_3]Cl_4$*

In a 20 mL vial zirconocene dichloride (112.5 mg, 0.385 mmol), 5-*tert*-butylisophthalic acid (25 mg, 0.113 mmol), and isophthalic acid (19 mg, 0.113 mmol) were dissolved in DMF (10 mL). The stock solution (1 mL) and DI water (150 μL) were transferred to 10 individual 4 mL vials and heated at 45 °C for 8.3 hours. White solid was collected via centrifugation followed by decanting the mother liquor. Material was activated under $N_2$ flow at 40 °C for 16 hours.

*$[Zr_{12}(\mu_3\text{-}O)_4(\mu_2\text{-}OH)_{12}(Cp)_{12}(5\text{-}tert\text{-}butyl\ bdc)_4(bdc)_2]Cl_4$*

In a 20 mL vial zirconocene dichloride (112.5 mg, 0.385 mmol), 5-*tert*-butylisophthalic acid (32.5 mg, 0.151 mmol), and isophthalic acid (12.5 mg, 0.0750 mmol) were dissolved in DMF (10 mL). The stock solution (1 mL) and DI water (150 μL) were transferred to 10 individual 4 mL vials and heated at 45 °C for 8.3 hours. White solid was collected via centrifugation followed by decanting the mother liquor.

*$[Zr_{12}(\mu_3\text{-}O)_4(\mu_2\text{-}OH)_{12}(Cp)_{12}(bdc)_3(5\text{-}OH\ bdc)_3]Cl_4$*

In a 20 mL vial, 2 mL stock solution (1), 4 mL stock solution (2), and 4 mL stock solution (7) were added. DI water (1500 μL) was then added to the vial and heated at 45 °C for 8 hours. White solid was collected via centrifugation followed by decanting the mother liquor. The solid was washed three times with DMF and three times with ethyl acetate. Solvent was replaced after 12 hours each wash.

*$[Zr_{12}(\mu_3\text{-}O)_4(\mu_2\text{-}OH)_{12}(Cp)_{12}(bdc)_3(5\text{-}NH_2\ bdc)_3]Cl_4$*

In a 20 mL vial, 2 mL stock solution (1), 4 mL stock solution (2), and 4 mL stock solution (8) were added. DI water (1500 μL) was then added to the vial and heated at 45 °C for 8 hours. White solid was collected via centrifugation followed by decanting the mother liquor. The solid was washed three times with DMF and three times with ethyl acetate. Solvent was replaced after 12 hours each wash.

*$[Zr_{12}(\mu_3\text{-}O)_4(\mu_2\text{-}OH)_{12}(Cp)_{12}(bdc)_3(5\text{-}CN\ bdc)_3]Cl_4$*

In a 20 mL vial, 2 mL stock solution (1), 4 mL stock solution (2), and 4 mL stock solution (9) were added. DI water (1500 μL) was then added to the vial and heated at 45 °C for 8 hours. White solid was collected via centrifugation followed by decanting the mother liquor. The solid was washed three times with DMF and three times with ethyl acetate. Solvent was replaced after 12 hours each wash.

*$[Zr_{12}(\mu_3\text{-}O)_4(\mu_2\text{-}OH)_{12}(Cp)_{12}(bdc)_3(5\text{-}benzyloxy\ bdc)_3]Cl_4$*

In a 20 mL vial, 2 mL stock solution (1), 4 mL stock solution (2), and 4 mL stock solution (10) were added. DI water (1500 μL) was then added to the vial and heated at 45 °C for

8 hours. White solid was collected via centrifugation followed by decanting the mother liquor. The solid was washed three times with DMF and three times with ethyl acetate. Solvent was replaced after 12 hours each wash.

*$[Zr_{12}(\mu_3\text{-}O)_4(\mu_2\text{-}OH)_{12}(Cp)_{12}(bdc)_3(5\text{-}Br\ bdc)_3]Cl_4$*

In a 20 mL vial, 2 mL stock solution (1), 4 mL stock solution (2), and 4 mL stock solution (6) were added. DI water (1500 µL) was then added to the vial and heated at 45 °C for 8 hours. White solid was collected via centrifugation followed by decanting the mother liquor. The solid was washed three times with DMF and three times with ethyl acetate. Solvent was replaced after 12 hours each wash.

*$[Zr_{12}(\mu_3\text{-}O)_4(\mu_2\text{-}OH)_{12}(Cp)_{12}(bdc)_3(5\text{-}NO_2\ bdc)_3]Cl_4$*

In a 20 mL vial, 2 mL stock solution (1), 4 mL stock solution (2), and 4 mL stock solution (11) were added. DI water (1500 µL) was then added to the vial and heated at 45 °C for 8 hours. White solid was collected via centrifugation followed by decanting the mother liquor. The solid was washed three times with DMF and three times with ethyl acetate. Solvent was replaced after 12 hours each wash.

*$[Zr_{12}(\mu_3\text{-}O)_4(\mu_2\text{-}OH)_{12}(Cp)_{12}(5\text{-}methyl\ bdc)_3(5\text{-}tert\text{-}butyl\ bdc)_3]Cl_4$*

In a 20 mL vial, 2 mL stock solution (1), 4 mL stock solution (4), and 4 mL stock solution (3) were added. DI water (1500 µL) was then added to the vial and heated at 45 °C for 8 hours. White solid was collected via centrifugation followed by decanting the mother liquor. The solid was washed three times with DMF and three times with ethyl acetate. Solvent was replaced after 12 hours each wash.

*$[Zr_{12}(\mu_3\text{-}O)_4(\mu_2\text{-}OH)_{12}(Cp)_{12}(5\text{-}methyl\ bdc)_3(m\text{-}dobdc)_3]Cl_4$*

In a 20 mL vial, 2 mL stock solution (1), 4 mL stock solution (4), and 4 mL stock solution (5) were added. DI water (1500 µL) was then added to the vial and heated at 45 °C for 8 hours. White solid was collected via centrifugation followed by decanting the mother liquor. The solid was washed three times with DMF and three times with ethyl acetate. Solvent was replaced after 12 hours each wash.

*$[Zr_{12}(\mu_3\text{-}O)_4(\mu_2\text{-}OH)_{12}(Cp)_{12}(5\text{-}methyl\ bdc)_3(5\text{-}CN\ bdc)_3]Cl_4$*

In a 20 mL vial, 2 mL stock solution (1), 4 mL stock solution (4), and 4 mL stock solution (9) were added. DI water (1500 µL) was then added to the vial and heated at 45 °C for 8 hours. White solid was collected via centrifugation followed by decanting the mother liquor. The solid was washed three times with DMF and three times with ethyl acetate. Solvent was replaced after 12 hours each wash.

*$[Zr_{12}(\mu_3\text{-}O)_4(\mu_2\text{-}OH)_{12}(Cp)_{12}(5\text{-}methyl\ bdc)_3(5\text{-}NO_2\ bdc)_3]Cl_4$*

In a 20 mL vial, 2 mL stock solution (1), 4 mL stock solution (4), and 4 mL stock solution (11) were added. DI water (1500 µL) was then added to the vial and heated at 45 °C for 8 hours. White solid was collected via centrifugation followed by decanting the mother liquor. The solid was washed three times with DMF and three times with ethyl acetate. Solvent was replaced after 12 hours each wash.

*[$Zr_{12}$($\mu_3$-O)$_4$($\mu_2$-OH)$_{12}$(Cp)$_{12}$(5-methyl bdc)$_3$(5-Br bdc)$_3$]$Cl_4$*

In a 20 mL vial, 2 mL stock solution (1), 4 mL stock solution (4), and 4 mL stock solution (6) were added. DI water (1500 µL) was then added to the vial and heated at 45 °C for 8 hours. White solid was collected via centrifugation followed by decanting the mother liquor. The solid was washed three times with DMF and three times with ethyl acetate. Solvent was replaced after 12 hours each wash.

*[$Zr_{12}$($\mu_3$-O)$_4$($\mu_2$-OH)$_{12}$(Cp)$_{12}$(5-methyl bdc)$_3$(5-benzyloxy bdc)$_3$]$Cl_4$*

In a 20 mL vial, 2 mL stock solution (1), 4 mL stock solution (4), and 4 mL stock solution (10) were added. DI water (1500 µL) was then added to the vial and heated at 45 °C for 8 hours. White solid was collected via centrifugation followed by decanting the mother liquor. The solid was washed three times with DMF and three times with ethyl acetate. Solvent was replaced after 12 hours each wash.

*[$Zr_{12}$($\mu_3$-O)$_4$($\mu_2$-OH)$_{12}$(Cp)$_{12}$(5-$NH_2$ bdc)$_3$(5-$NO_2$ bdc)$_3$]$Cl_4$*

In a 20 mL vial, 2 mL stock solution (1), 4 mL stock solution (8), and 4 mL stock solution (11) were added. DI water (1500 µL) was then added to the vial and heated at 45 °C for 8 hours. White solid was collected via centrifugation followed by decanting the mother liquor. The solid was washed three times with DMF and three times with ethyl acetate. Solvent was replaced after 12 hours each wash.

*[$Zr_{12}$($\mu_3$-O)$_4$($\mu_2$-OH)$_{12}$(Cp)$_{12}$(5-OH bdc)$_3$(5-$NO_2$ bdc)$_3$]$Cl_4$*

In a 20 mL vial, 2 mL stock solution (1), 4 mL stock solution (7), and 4 mL stock solution (11) were added. DI water (1500 µL) was then added to the vial and heated at 45 °C for 8 hours. White solid was collected via centrifugation followed by decanting the mother liquor. The solid was washed three times with DMF and three times with ethyl acetate. Solvent was replaced after 12 hours each wash.

*[$Zr_{12}$($\mu_3$-O)$_4$($\mu_2$-OH)$_{12}$(Cp)$_{12}$(5-CN bdc)$_3$(m-dobdc)$_3$]$Cl_4$*

In a 20 mL vial, 2 mL stock solution (1), 4 mL stock solution (9), and 4 mL stock solution (5) were added. DI water (1500 µL) was then added to the vial and heated at 45 °C for 8 hours. White solid was collected via centrifugation followed by decanting the mother liquor. The solid was washed three times with DMF and three times with ethyl acetate. Solvent was replaced after 12 hours each wash.

*[$Zr_{12}$($\mu_3$-O)$_4$($\mu_2$-OH)$_{12}$(Cp)$_{12}$(5-CN bdc)$_3$(5-$NO_2$ bdc)$_3$]$Cl_4$*

In a 20 mL vial, 2 mL stock solution (1), 4 mL stock solution (9), and 4 mL stock solution (11) were added. DI water (1500 µL) was then added to the vial and heated at 45 °C for 8 hours. White solid was collected via centrifugation followed by decanting the mother liquor. The solid was washed three times with DMF and three times with ethyl acetate. Solvent was replaced after 12 hours each wash.

*[$Zr_{12}$($\mu_3$-O)$_4$($\mu_2$-OH)$_{12}$(Cp)$_{12}$(5-CN bdc)$_3$(5-Br bdc)$_3$]$Cl_4$*

In a 20 mL vial, 2 mL stock solution (1), 4 mL stock solution (9), and 4 mL stock solution (6) were added. DI water (1500 µL) was then added to the vial and heated at 45 °C for 8 hours. White solid was collected via centrifugation followed by decanting the mother

liquor. The solid was washed three times with DMF and three times with ethyl acetate. Solvent was replaced after 12 hours each wash.

*$[Zr_{12}(\mu_3\text{-}O)_4(\mu_2\text{-}OH)_{12}(Cp)_{12}(5\text{-}CN\ bdc)_3(5\text{-}benzyloxy\ bdc)_3]Cl_4$*

In a 20 mL vial, 2 mL stock solution (1), 4 mL stock solution (9), and 4 mL stock solution (10) were added. DI water (1500 µL) was then added to the vial and heated at 45 °C for 8 hours. White solid was collected via centrifugation followed by decanting the mother liquor. The solid was washed three times with DMF and three times with ethyl acetate. Solvent was replaced after 12 hours each wash.

*$[Zr_{12}(\mu_3\text{-}O)_4(\mu_2\text{-}OH)_{12}(Cp)_{12}(m\text{-}dobdc)_3(5\text{-}NO_2\ bdc)_3]Cl_4$*

In a 20 mL vial, 2 mL stock solution (1), 4 mL stock solution (5), and 4 mL stock solution (11) were added. DI water (1500 µL) was then added to the vial and heated at 45 °C for 8 hours. White solid was collected via centrifugation followed by decanting the mother liquor. The solid was washed three times with DMF and three times with ethyl acetate. Solvent was replaced after 12 hours each wash.

*$[Zr_{12}(\mu_3\text{-}O)_4(\mu_2\text{-}OH)_{12}(Cp)_{12}(m\text{-}dobdc)_3(5\text{-}tert\text{-}butyl\ bdc)_3]Cl_4$*

In a 20 mL vial, 2 mL stock solution (1), 4 mL stock solution (5), and 4 mL stock solution (3) were added. DI water (1500 µL) was then added to the vial and heated at 45 °C for 8 hours. White solid was collected via centrifugation followed by decanting the mother liquor. The solid was washed three times with DMF and three times with ethyl acetate. Solvent was replaced after 12 hours each wash.

*$[Zr_{12}(\mu_3\text{-}O)_4(\mu_2\text{-}OH)_{12}(Cp)_{12}(m\text{-}dobdc)_3(5\text{-}Br\ bdc)_3]Cl_4$*

In a 20 mL vial, 2 mL stock solution (1), 4 mL stock solution (5), and 4 mL stock solution (6) were added. DI water (1500 µL) was then added to the vial and heated at 45 °C for 8 hours. White solid was collected via centrifugation followed by decanting the mother liquor. The solid was washed three times with DMF and three times with ethyl acetate. Solvent was replaced after 12 hours each wash.

*$[Zr_{12}(\mu_3\text{-}O)_4(\mu_2\text{-}OH)_{12}(Cp)_{12}(m\text{-}dobdc)_3(5\text{-}benzyloxy\ bdc)_3]Cl_4$*

In a 20 mL vial, 2 mL stock solution (1), 4 mL stock solution (5), and 4 mL stock solution (10) were added. DI water (1500 µL) was then added to the vial and heated at 45 °C for 8 hours. White solid was collected via centrifugation followed by decanting the mother liquor. The solid was washed three times with DMF and three times with ethyl acetate. Solvent was replaced after 12 hours each wash.

*$[Zr_{12}(\mu_3\text{-}O)_4(\mu_2\text{-}OH)_{12}(Cp)_{12}(5\text{-}NO_2\ bdc)_3(5\text{-}tert\text{-}butyl\ bdc)_3]Cl_4$*

In a 20 mL vial, 2 mL stock solution (1), 4 mL stock solution (11), and 4 mL stock solution (3) were added. DI water (1500 µL) was then added to the vial and heated at 45 °C for 8 hours. White solid was collected via centrifugation followed by decanting the mother liquor. The solid was washed three times with DMF and three times with ethyl acetate. Solvent was replaced after 12 hours each wash.

*$[Zr_{12}(\mu_3\text{-}O)_4(\mu_2\text{-}OH)_{12}(Cp)_{12}(5\text{-}NO_2\ bdc)_3(5\text{-}Br\ bdc)_3]Cl_4$*

In a 20 mL vial, 2 mL stock solution (1), 4 mL stock solution (11), and 4 mL stock solution (6) were added. DI water (1500 µL) was then added to the vial and heated at 45 °C for 8 hours. White solid was collected via centrifugation followed by decanting the mother liquor. The solid was washed three times with DMF and three times with ethyl acetate. Solvent was replaced after 12 hours each wash.

*$[Zr_{12}(\mu_3\text{-}O)_4(\mu_2\text{-}OH)_{12}(Cp)_{12}(5\text{-}NO_2\ bdc)_3(5\text{-}benxyloxy\ bdc)_3]Cl_4$*

In a 20 mL vial, 2 mL stock solution (1), 4 mL stock solution (11), and 4 mL stock solution (10) were added. DI water (1500 µL) was then added to the vial and heated at 45 °C for 8 hours. White solid was collected via centrifugation followed by decanting the mother liquor. The solid was washed three times with DMF and three times with ethyl acetate. Solvent was replaced after 12 hours each wash.

*$[Zr_{12}(\mu_3\text{-}O)_4(\mu_2\text{-}OH)_{12}(Cp)_{12}(5\text{-}Br\ bdc)_3(5\text{-}benzyloxy\ bdc)_3]Cl_4$*

In a 20 mL vial, 2 mL stock solution (1), 4 mL stock solution (6), and 4 mL stock solution (10) were added. DI water (1500 µL) was then added to the vial and heated at 45 °C for 8 hours. White solid was collected via centrifugation followed by decanting the mother liquor. The solid was washed three times with DMF and three times with ethyl acetate. Solvent was replaced after 12 hours each wash.

*$[Zr_{12}(\mu_3\text{-}O)_4(\mu_2\text{-}OH)_{12}(Cp)_{12}(1,3\text{-}bdc)_4(5\text{-}N_3)_2]Cl_4$*

In a 20 mL vial, 1,3-bdc (100 mg, 0.602 mmol) was dissolved in DMF (16 mL). In a second 20 mL vial, 5-$N_3$ (62 mg, 0.300 mmol) was dissolved in DMF (16 mL). In a 50 mL VWR jar, zirconocene dichloride (1.356 g, 4.63 mmol) was dissolved in DMF (24 mL). To a third 20 mL vial, 4 mL of the 1,3-bdc solution was added, 4 mL of the 5-N3 solution was added, and 2 mL of the zirconocene dichloride solution was added. DI water (1500 µL) was then added to the vial and heated at 45 °C for 8 hours. The solid was washed three times with DMF and three times with chloroform. Solvent was replaced after 2 hours each wash.

<u>Control Experiments</u>

In a 20 mL vial, $[Zr_{12}(\mu_3\text{-}O)_4(\mu_2\text{-}OH)_{12}(Cp)_{12}(1,3\text{-}bdc)_6]Cl_4$ (16 mg, 0.0547 mmol) was added and dissolved in 12 mL DMF. In another 20 mL vial, 5-methylisophthalic acid (3.5 mg, 0.0194 mmol) was dissolved in DMF (3 mL). In another 20 mL vial, MPA (1.7 mg, 0.0064 mmol) was dissolved in DMF. In another 20 mL vial, 5-*tert*-butylisophthalic acid (2.1 mg, 0.0094 mmol) was dissolved in DMF. In another 20 mL vial, *m*-dobdc[2] (1.9 mg, 0.0096 mmol) was dissolved in DMF. To each vial with ligand in it, 3 mL of the $[Zr_{12}(\mu_3\text{-}O)_4(\mu_2\text{-}OH)_{12}(Cp)_{12}(1,3\text{-}bdc)_6]^{4+}$ stock solution was added. These four vials were left to stir at room temperature for 24 hours. Samples were centrifuged and white solid was collected.

Click Reaction

*$[Zr_{12}(\mu_3\text{-}O)_4(\mu_2\text{-}OH)_{12}(Cp)_{12}(1,3\text{-}bdc)_4(5\text{-}N_3\text{-}PrOH)_2]Cl_4$*

Adapted from a previously reported procedure.[4] $[Zr_{12}(\mu_3\text{-}O)_4(\mu_2\text{-}OH)_{12}(Cp)_{12}(1,3\text{-}bdc)_4(5\text{-}N_3)_2]Cl_4$ (50 mg, 0.0130 mmol) was dissolved in dry DMF (20 mL) in a 50 mL vial. Trimethylamine (70 µL) and propargyl alcohol (100 µL) were also added to this vessel. The vial was capped with a septa and sparged for 10 min with nitrogen. To a 20 mL vial, CuI (2 mg) was dissolved in dry acetonitrile (2 mL). This vial was capped with a septa and sparged with nitrogen for 5 min. This CuI solution was injected into the cage solution under active nitrogen, maintaining an air-free environment. The reaction continued at room temperature for 14 hours, after which, the solution was centrifuged and decanted into a new vial to remove any impurities. The product was precipitated via addition of excess diethyl ether to the decanted solution. The precipitate was isolated via centrifugation and washed with diethyl ether three times, with solvent replaced every two hours before drying under vacuum.

## Gas Adsorption Measurements

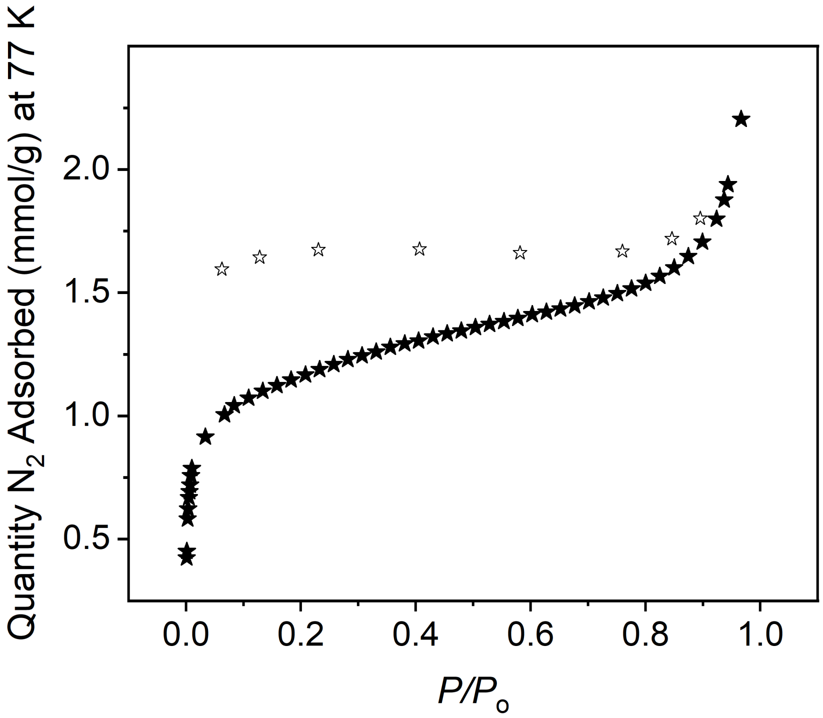


**Figure 1.** $N_2$ adsorption (solid black stars) and desorption (hollow black stars) for the heteroleptic 3:3 1,3-bdc:5-methyl cage.

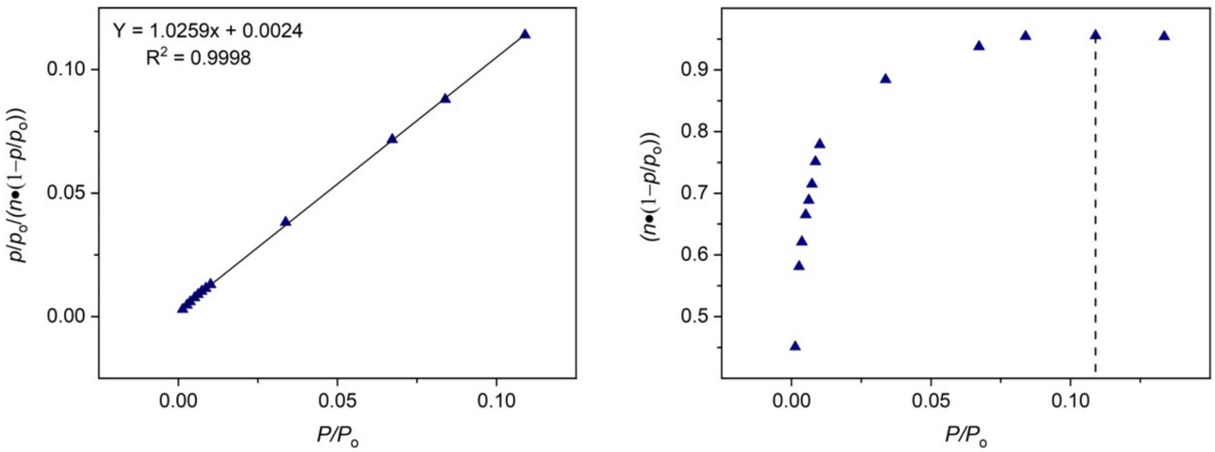


**Figure 2.** Left: The slope of the best fit line for $P/P_0 < 0.109$ is 1.0259 and the y-intercept is 0.0024, which satisfies the second BET consistency criterion. This results in a measured surface area of 99.8 $m^2/g$ to $N_2$. Right: Plot of $n(1-P/P_0)$ vs. $P/P_0$ to determine the maximum $P/P_0$ used in the BET linear fit according to the first BET consistency criterion for $N_2$ adsorption at 77 K for the 3:3 1,3-bdc:5-methyl cage.

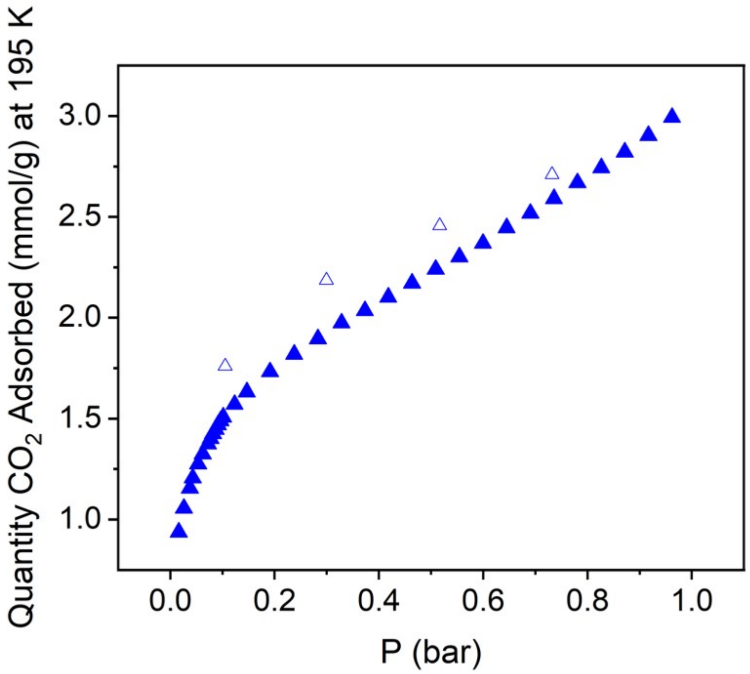


**Figure 3.** $CO_2$ adsorption (solid blue stars) and desorption (hollow blue stars) for the heteroleptic 3:3 1,3-bdc:*m*-dobdc cage.

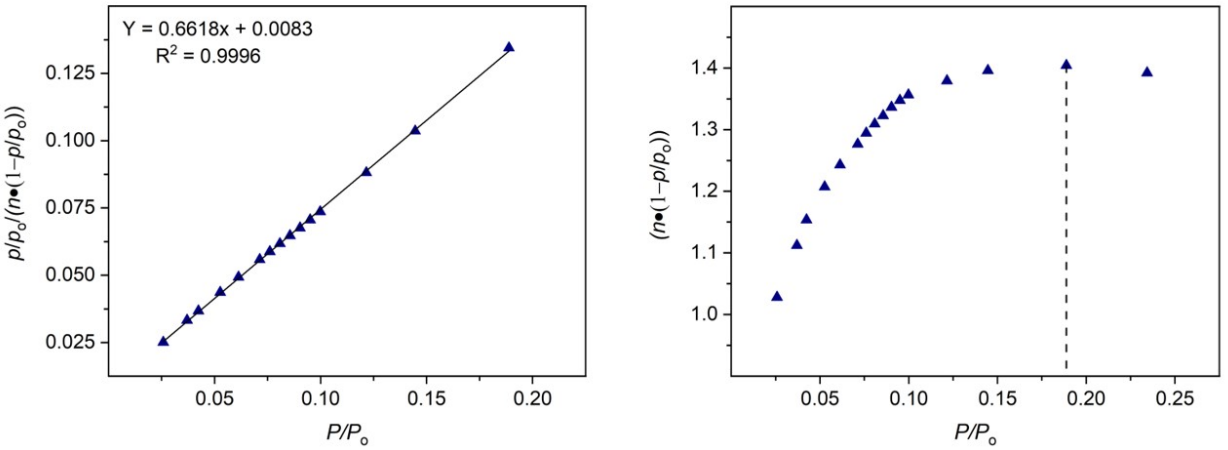


**Figure 4.** Left: The slope of the best fit line for $P/P_0 < 0.189$ is 0.6618 and the y-intercept is 0.0083, which satisfies the second BET consistency criterion. This results in a measured surface area of 154 $m^2/g$ to $CO_2$. Right: Plot of $n(1-P/P_0)$ vs. $P/P_0$ to determine the maximum $P/P_0$ used in the BET linear fit according to the first BET consistency criterion for $CO_2$ adsorption at 195 K for the 3:3 1,3-bdc:*m*-dobdc cage.

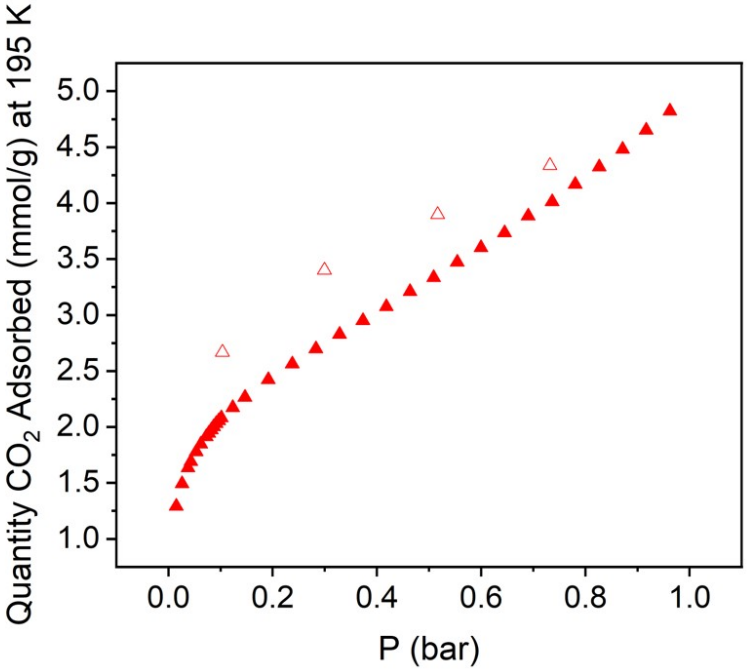


**Figure 5.** $CO_2$ adsorption (solid red stars) and desorption (hollow red stars) for the heteroleptic 3:3 1,3-bdc:5-MPA cage.

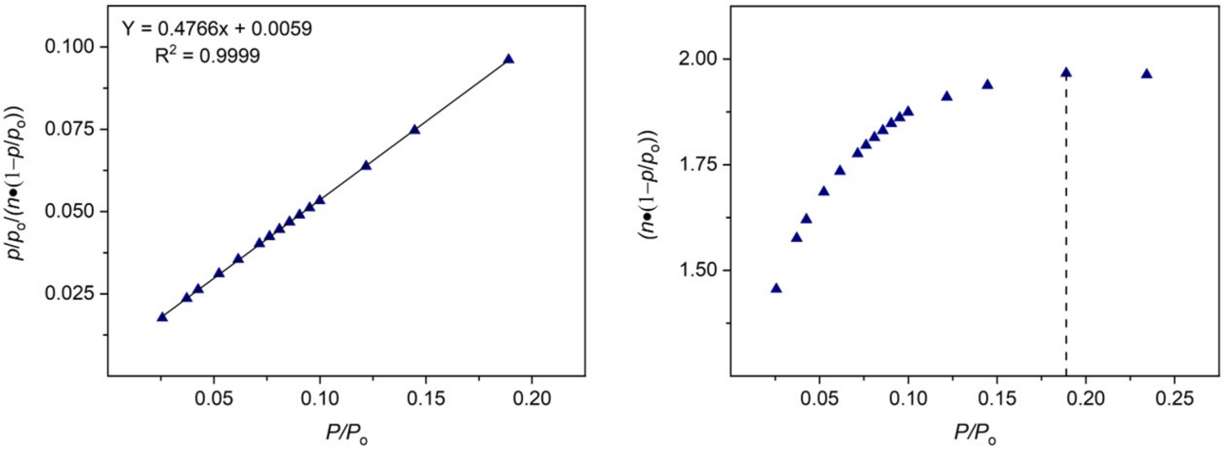


**Figure 6.** Left: The slope of the best fit line for $P/P_0 < 0.189$ is 0.4766 and the y-intercept is 0.0059, which satisfies the second BET consistency criterion. This results in a measured surface area of 214 $m^2/g$ to $CO_2$. Right: Plot of $n(1-P/P_0)$ vs. $P/P_0$ to determine the maximum $P/P_0$ used in the BET linear fit according to the first BET consistency criterion for $CO_2$ adsorption at 195 K for the 3:3 1,3-bdc:5-MPA cage.

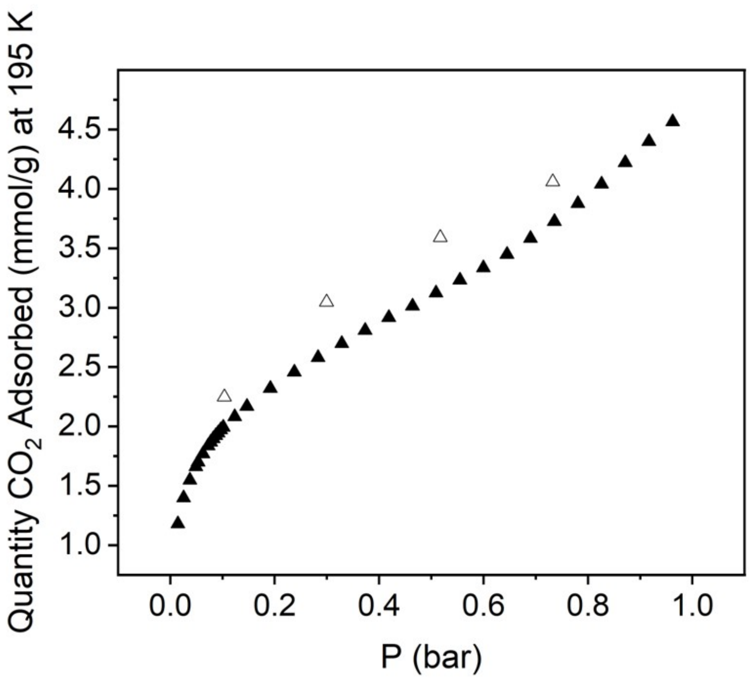


**Figure 7.** $CO_2$ adsorption (solid black stars) and desorption (hollow black stars) for the heteroleptic 3:3 1,3-bdc:5-*tert*-butyl cage.

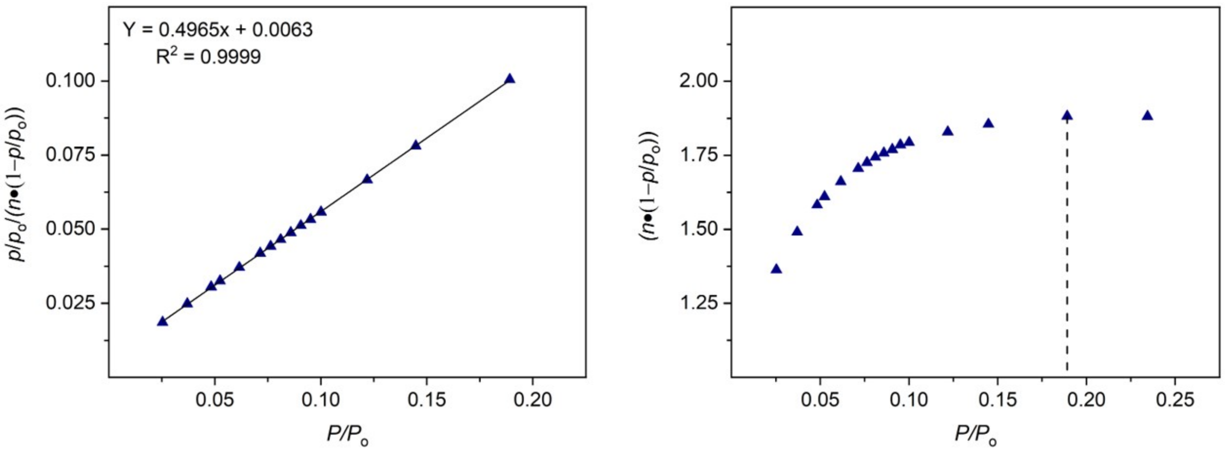


**Figure 8.** Left: The slope of the best fit line for $P/P_0 < 0.189$ is 0.4965 and the y-intercept is 0.0063, which satisfies the second BET consistency criterion. This results in a measured surface area of 206 $m^2/g$ to $CO_2$. Right: Plot of $n(1-P/P_0)$ vs. $P/P_0$ to determine the maximum $P/P_0$ used in the BET linear fit according to the first BET consistency criterion for $CO_2$ adsorption at 195 K for the 3:3 1,3-bdc:5-*tert*-butyl cage.

# $^1$H-NMR Spectra

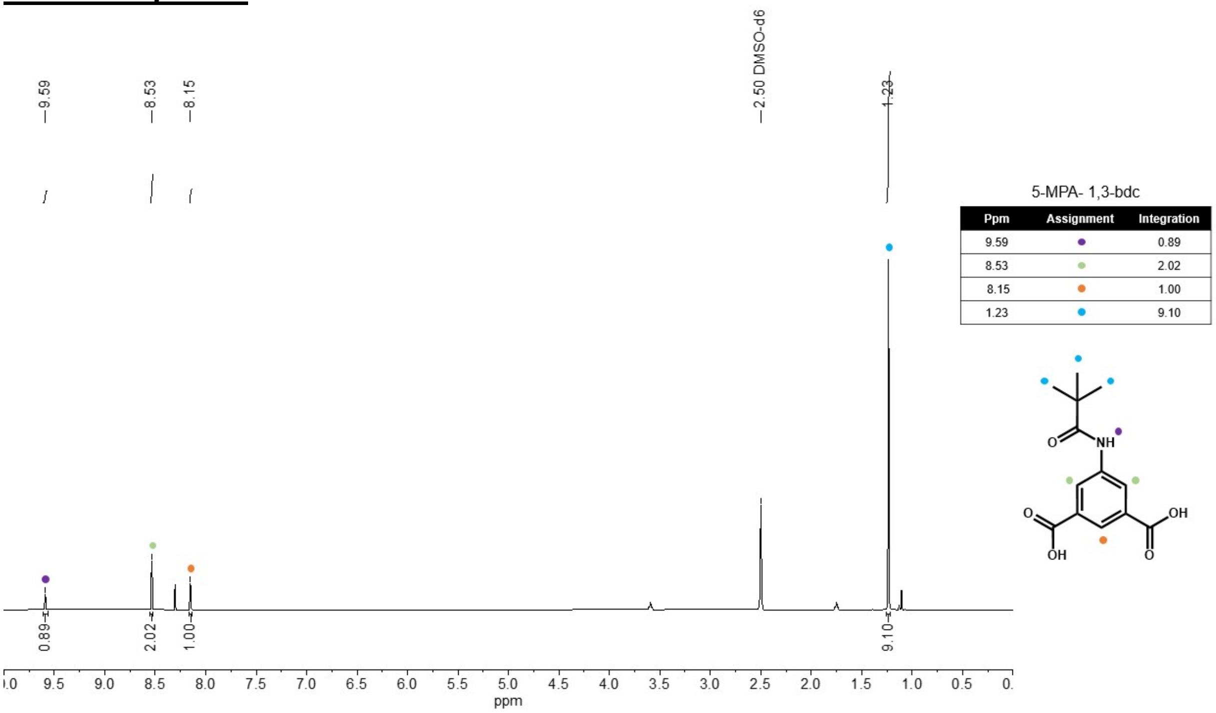


**Figure 9.** $^1$H NMR for the 5-MPA ligand in DMSO-$d_6$.

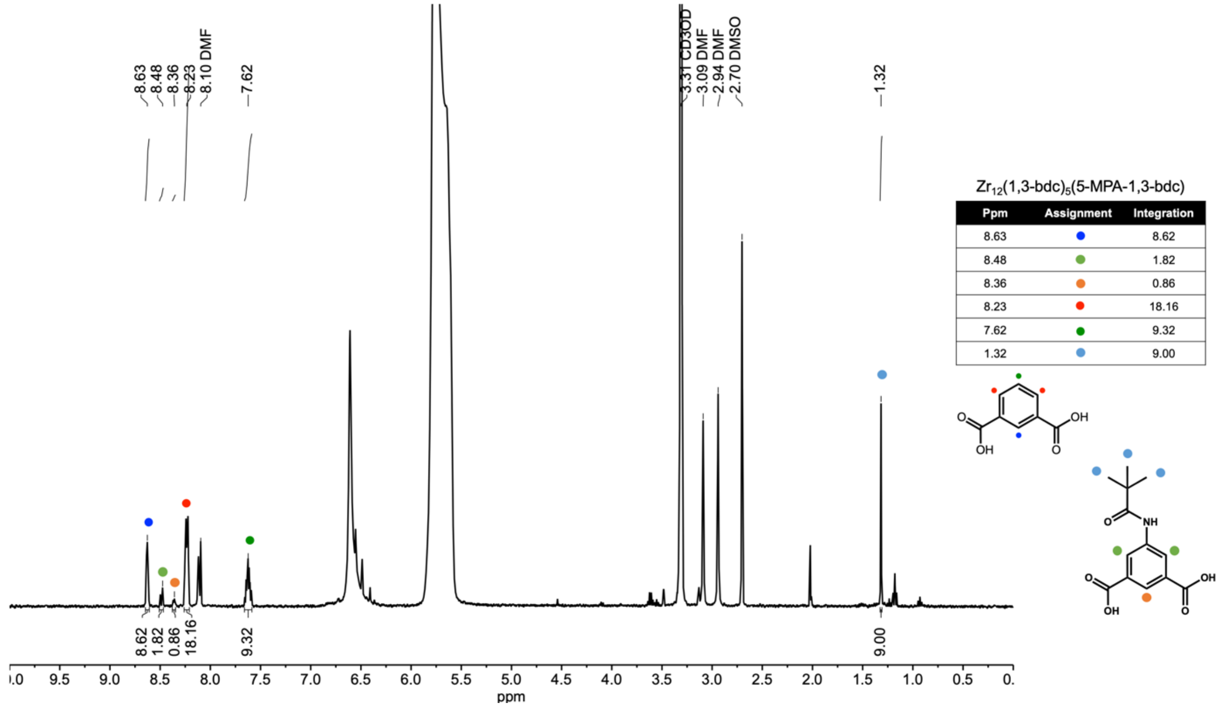


**Figure 10.** $^1$H NMR for the 1,3-bdc: 5-MPA heteroleptic cage with a $L^1$:$L^2$ ligand ratio of 5:1 in MeOH-$d_4$.

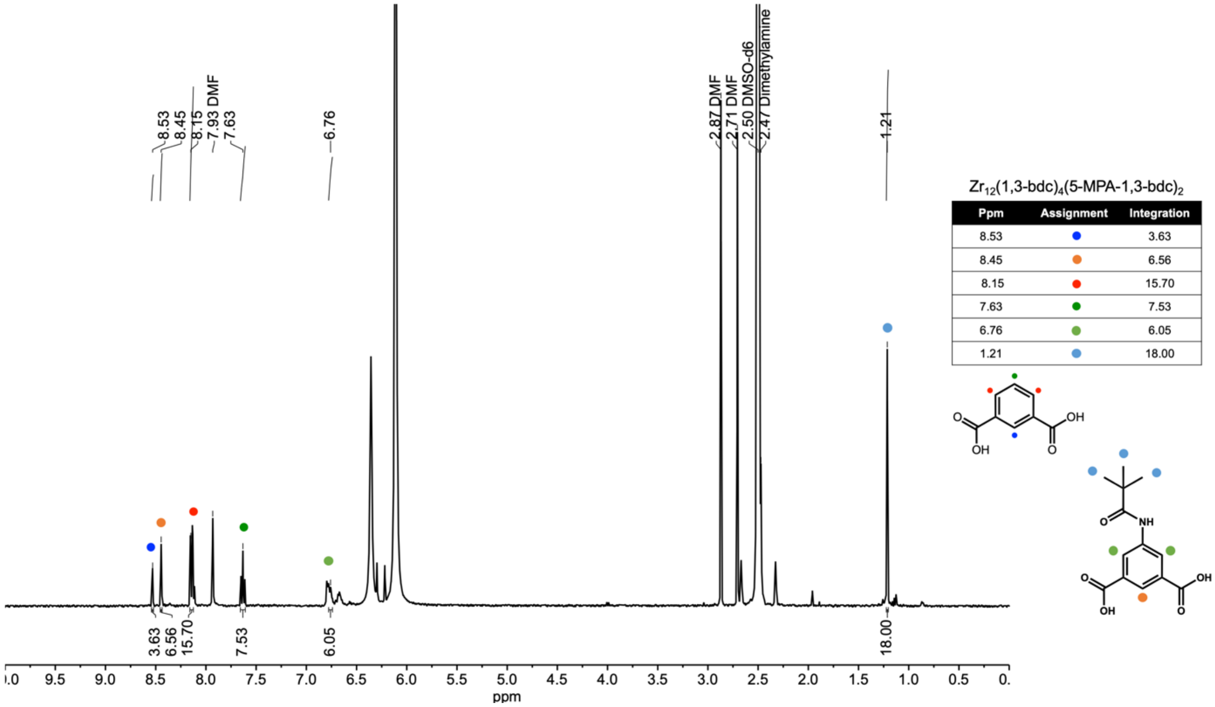


**Figure 11.** $^1$H NMR for the 1,3-bdc: 5-MPA heteroleptic cage with a $L^1$:$L^2$ ligand ratio of 4:2 in DMSO-$d_6$.

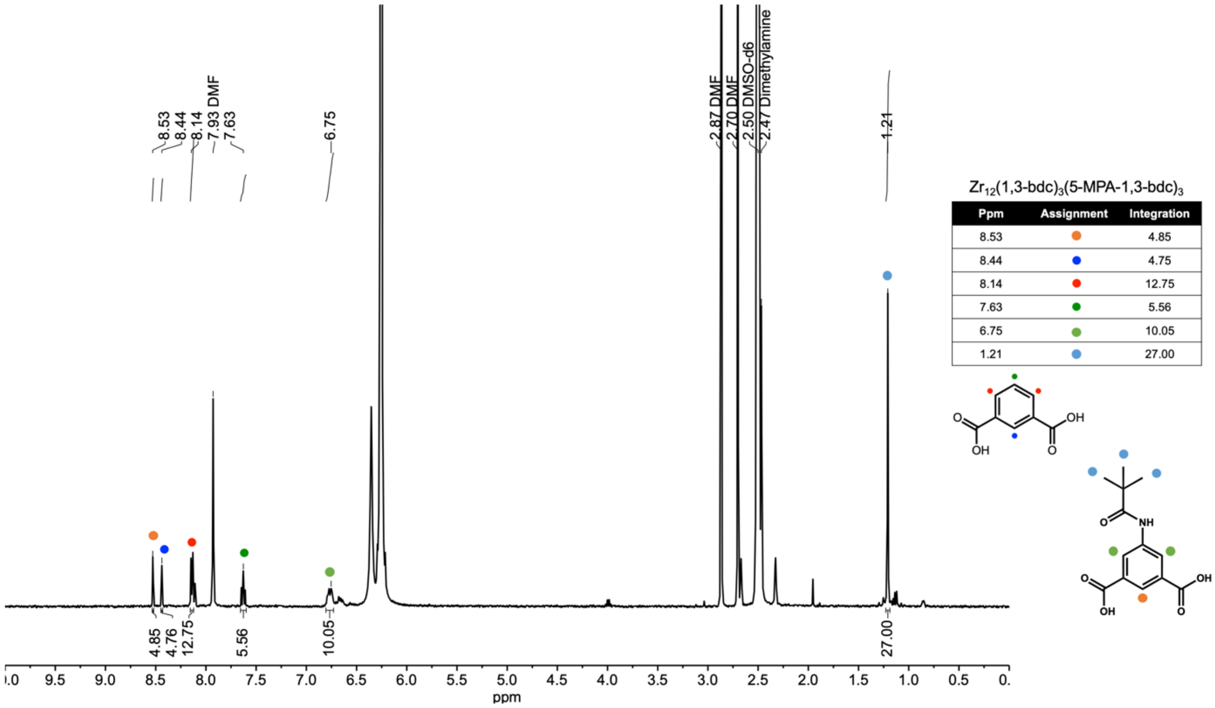


**Figure 12.** $^1$H NMR for the 1,3-bdc: 5-MPA heteroleptic cage with a $L^1$:$L^2$ ligand ratio of 3:3 in DMSO-$d_6$.

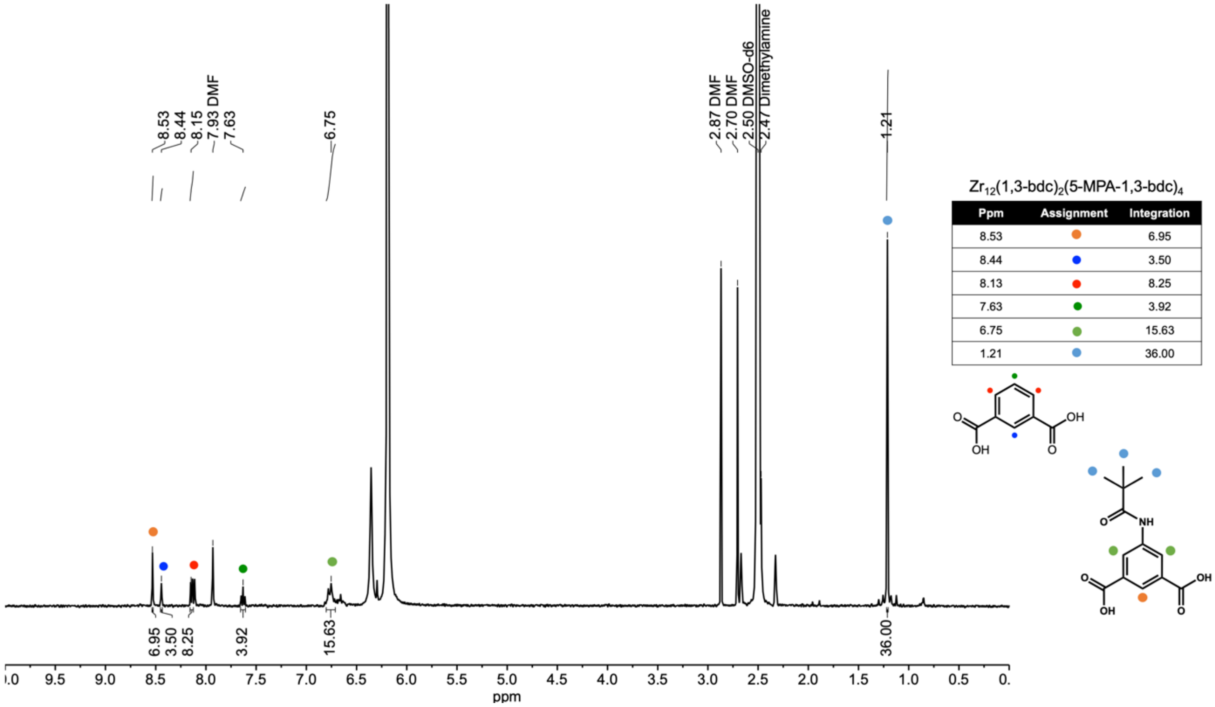


**Figure 13.** $^1H$ NMR for the 1,3-bdc: 5-MPA heteroleptic cage with a $L^1:L^2$ ligand ratio of 2:4 in DMSO-$d_6$.

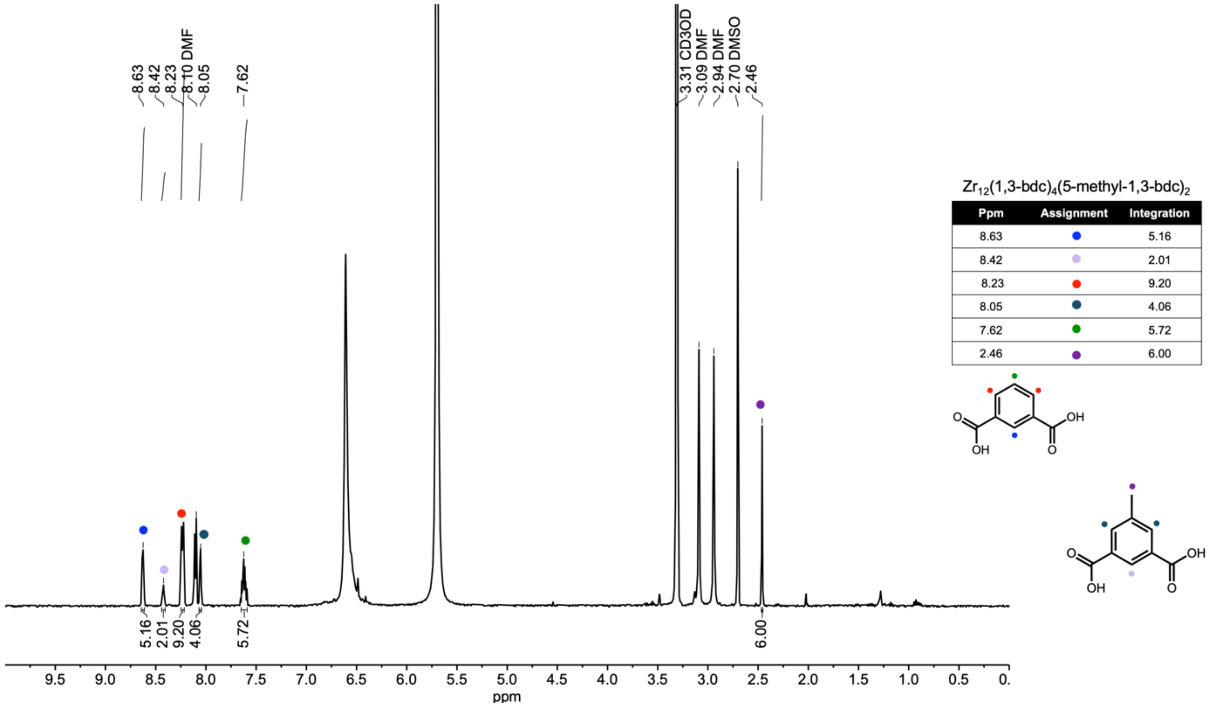


**Figure 14.** $^1H$ NMR for the 1,3-bdc: 5-methyl heteroleptic cage with a $L^1:L^2$ ligand ratio of 4:2 in MeOH-$d_4$.

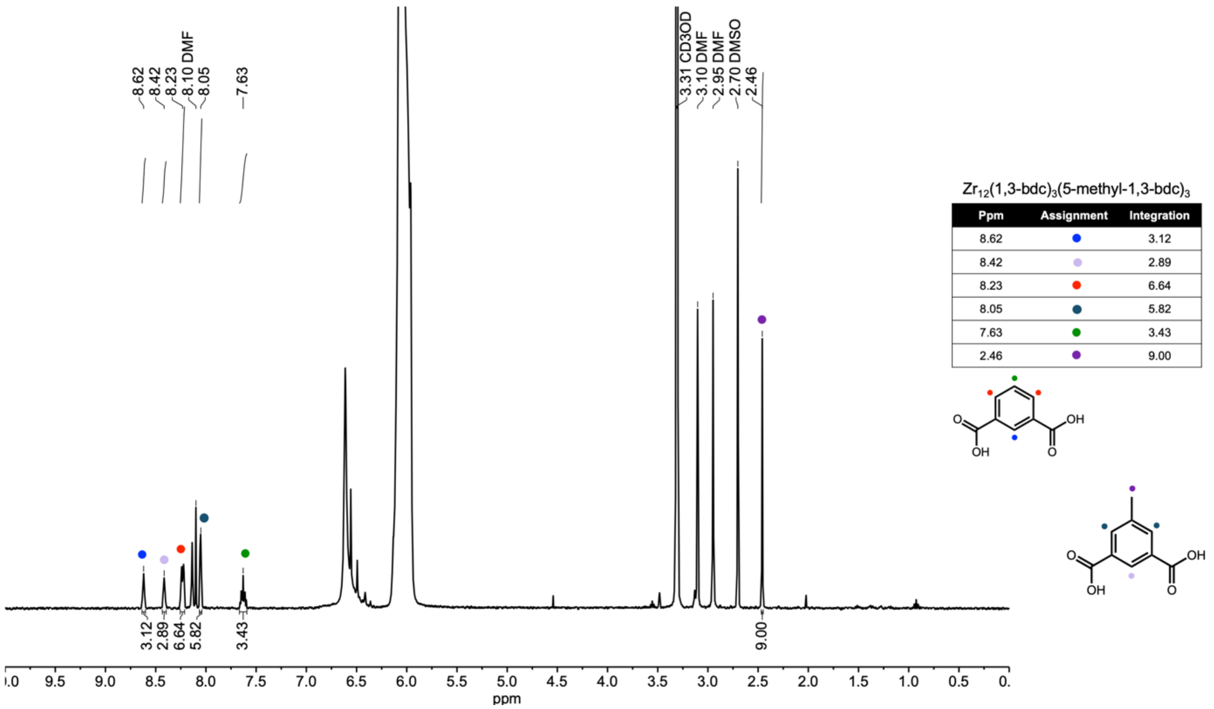


**Figure 15.** $^1$H NMR for the 1,3-bdc: 5-methyl heteroleptic cage with a $L^1$:$L^2$ ligand ratio of 3:3 in MeOH-$d_4$.

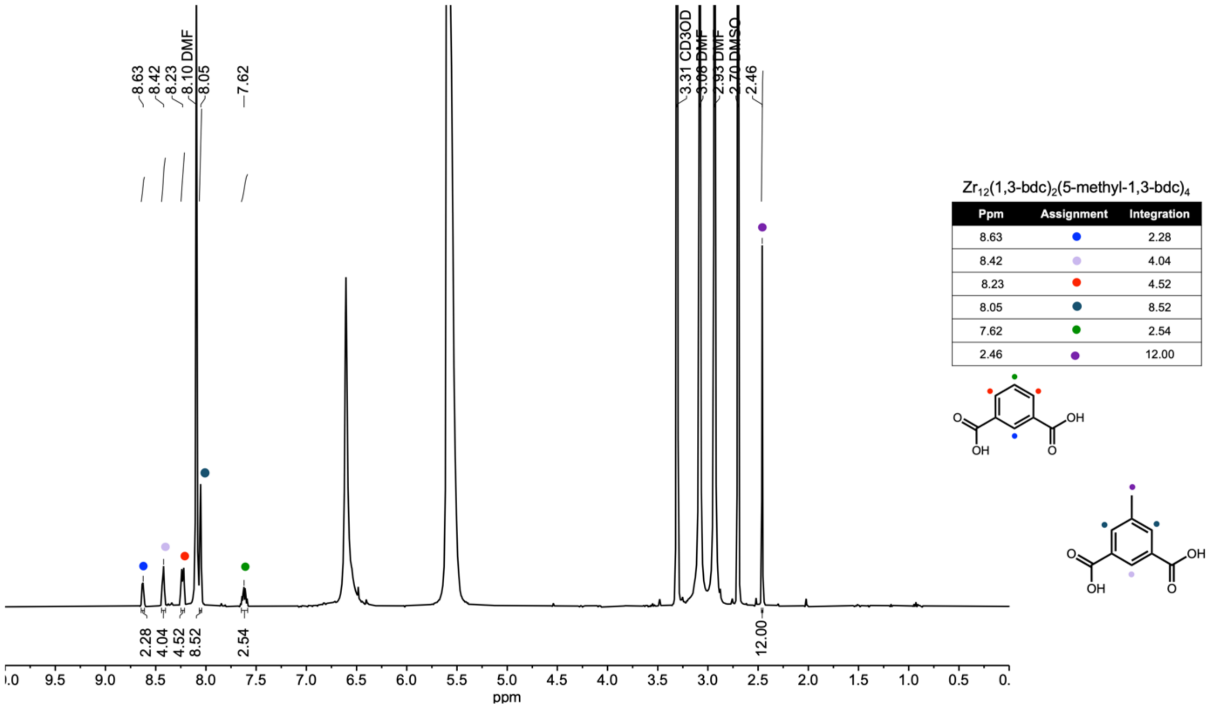


**Figure 16.** $^1$H NMR for the 1,3-bdc: 5-methyl heteroleptic cage with a $L^1$:$L^2$ ligand ratio of 2:4 in MeOH-$d_4$.

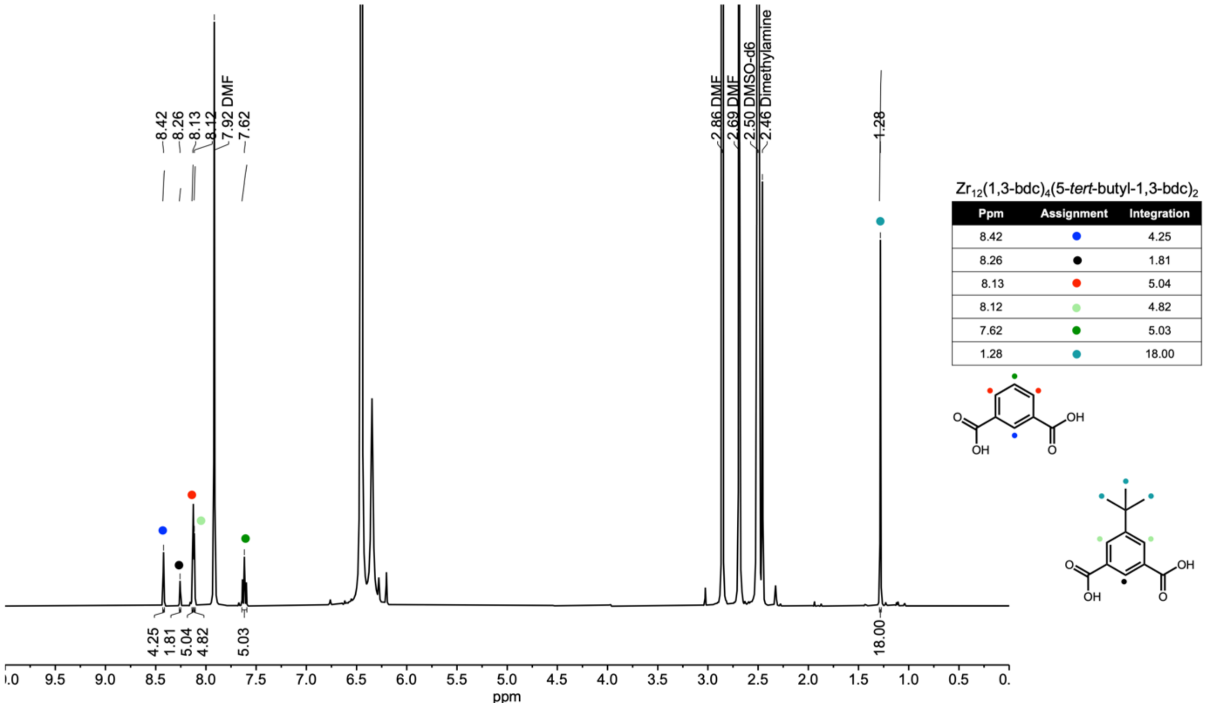


**Figure 17.** $^{1}H$ NMR for the 1,3-bdc: 5-*tert*-butyl heteroleptic cage with a $L^1$:$L^2$ ligand ratio of 4:2 in DMSO-$d_6$.

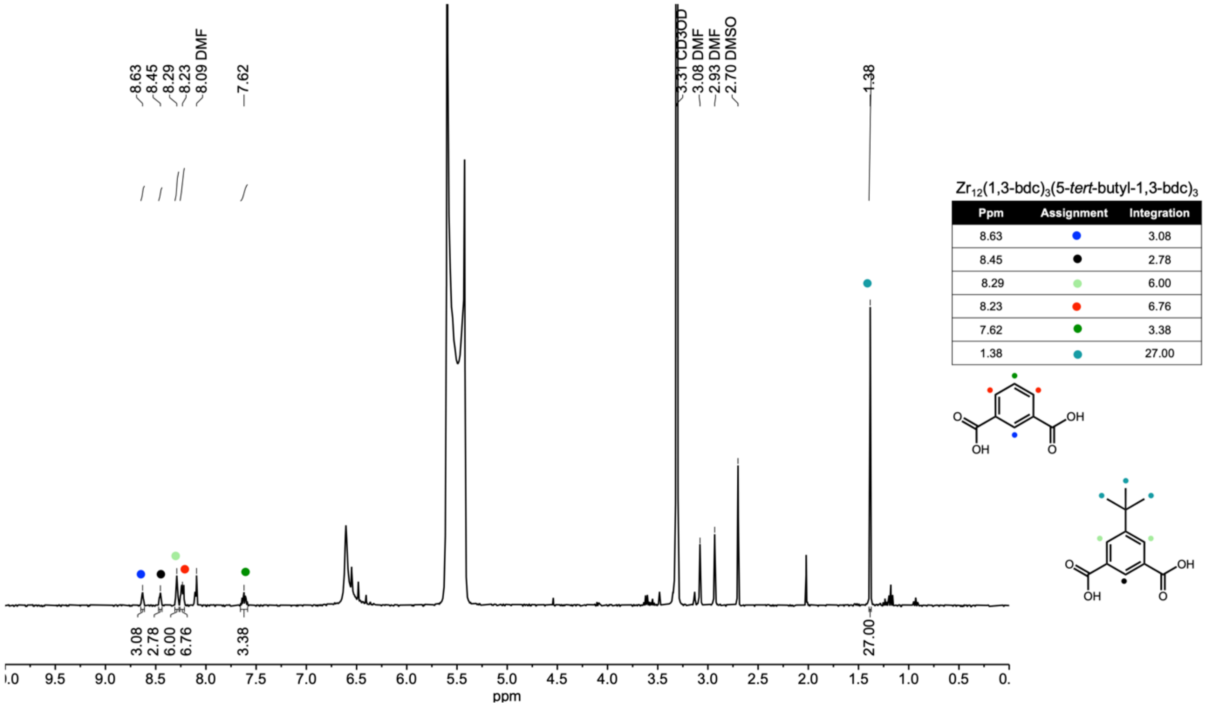


**Figure 18.** $^{1}H$ NMR for the 1,3-bdc: 5-*tert*-butyl heteroleptic cage with a $L^1$:$L^2$ ligand ratio of 3:3 in MeOH-$d_4$.

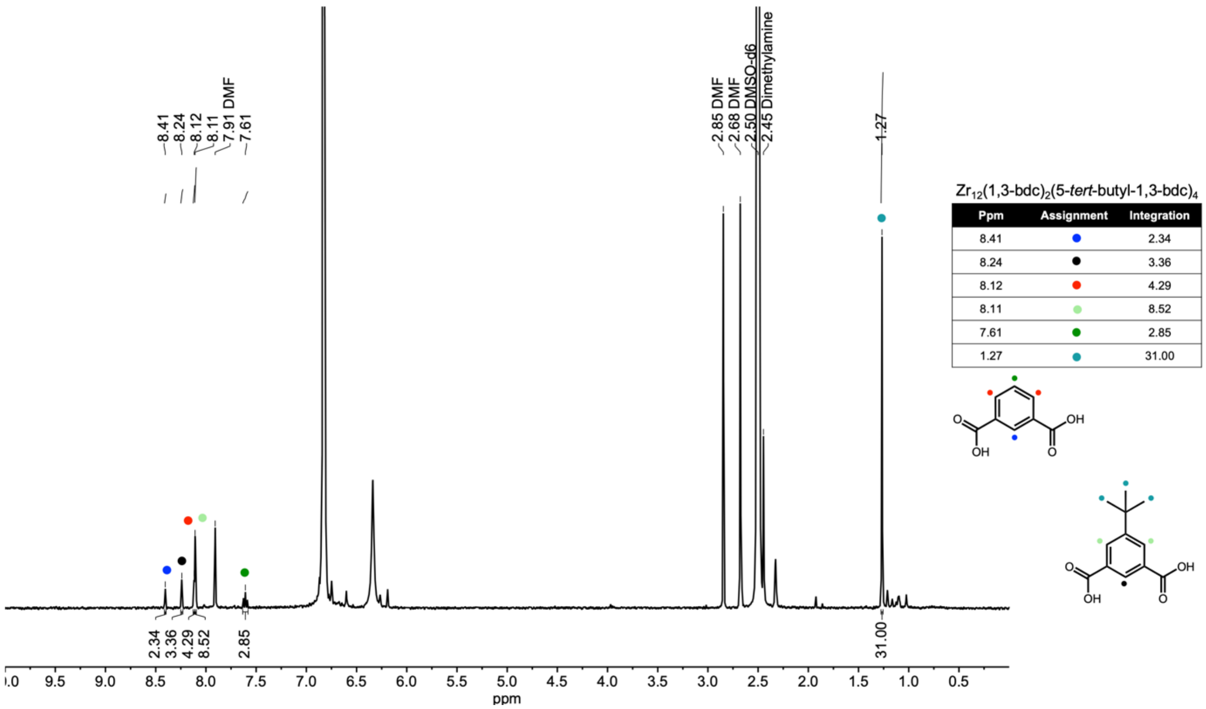


Zr$_{12}$(1,3-bdc)$_2$(5-*tert*-butyl-1,3-bdc)$_4$

| Ppm | Assignment | Integration |
|---|---|---|
| 8.41 | ● (blue) | 2.34 |
| 8.24 | ● (black) | 3.36 |
| 8.12 | ● (red) | 4.29 |
| 8.11 | ● (light green) | 8.52 |
| 7.61 | ● (green) | 2.85 |
| 1.27 | ● (teal) | 31.00 |

**Figure 19.** $^1$H NMR for the 1,3-bdc: 5-*tert*-butyl heteroleptic cage with a L$^1$:L$^2$ ligand ratio of 2:4 in DMSO-d$_6$.

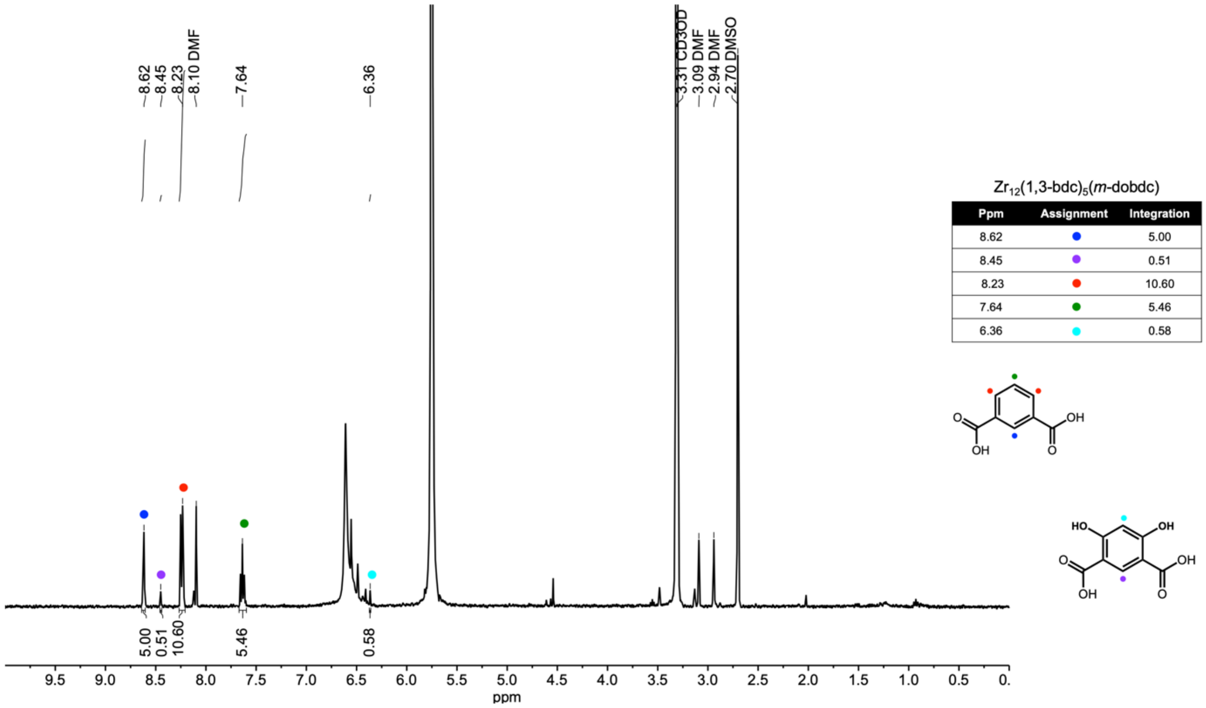


Zr$_{12}$(1,3-bdc)$_5$(*m*-dobdc)

| Ppm | Assignment | Integration |
|---|---|---|
| 8.62 | ● (blue) | 5.00 |
| 8.45 | ● (purple) | 0.51 |
| 8.23 | ● (red) | 10.60 |
| 7.64 | ● (green) | 5.46 |
| 6.36 | ● (cyan) | 0.58 |

**Figure 20.** $^1$H NMR for the 1,3-bdc: *m*-dobdc heteroleptic cage with a L$^1$:L$^2$ ligand ratio of 5:1 in MeOH-d$_4$.

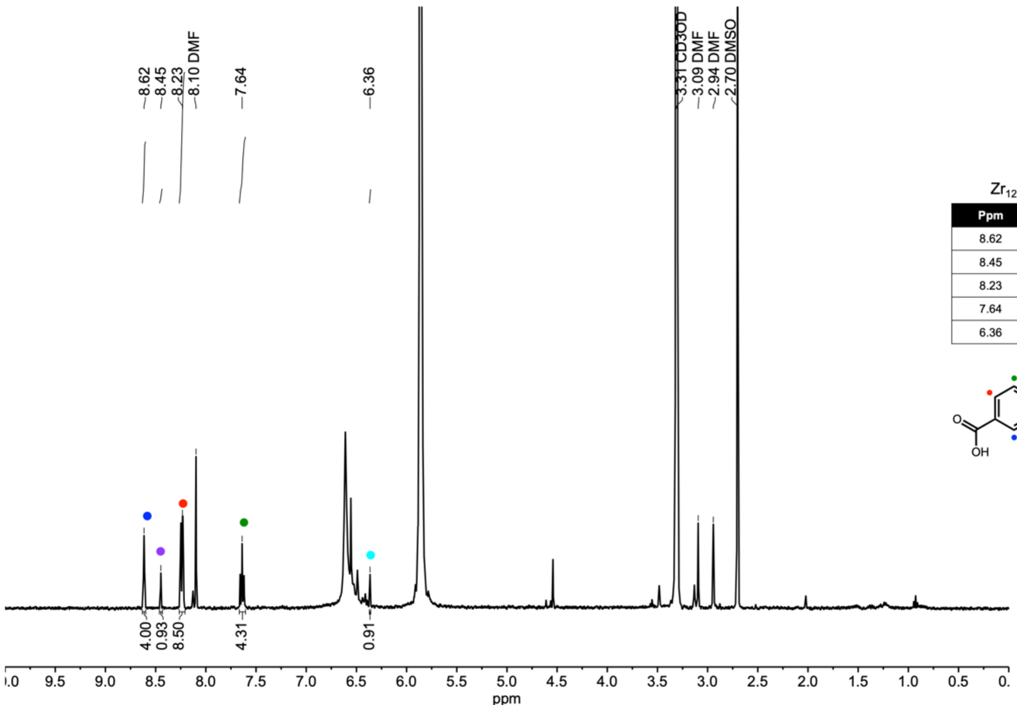


Zr$_{12}$(1,3-bdc)$_4$(*m*-dobdc)$_2$

| Ppm | Assignment | Integration |
|---|---|---|
| 8.62 | ● (blue) | 4.00 |
| 8.45 | ● (purple) | 0.93 |
| 8.23 | ● (red) | 8.50 |
| 7.64 | ● (green) | 4.31 |
| 6.36 | ● (cyan) | 0.91 |

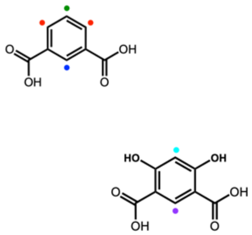


**Figure 21.** $^1$H NMR for the 1,3-bdc: *m*-dobdc heteroleptic cage with a L$^1$:L$^2$ ligand ratio of 4:2 in MeOH-d$_4$.

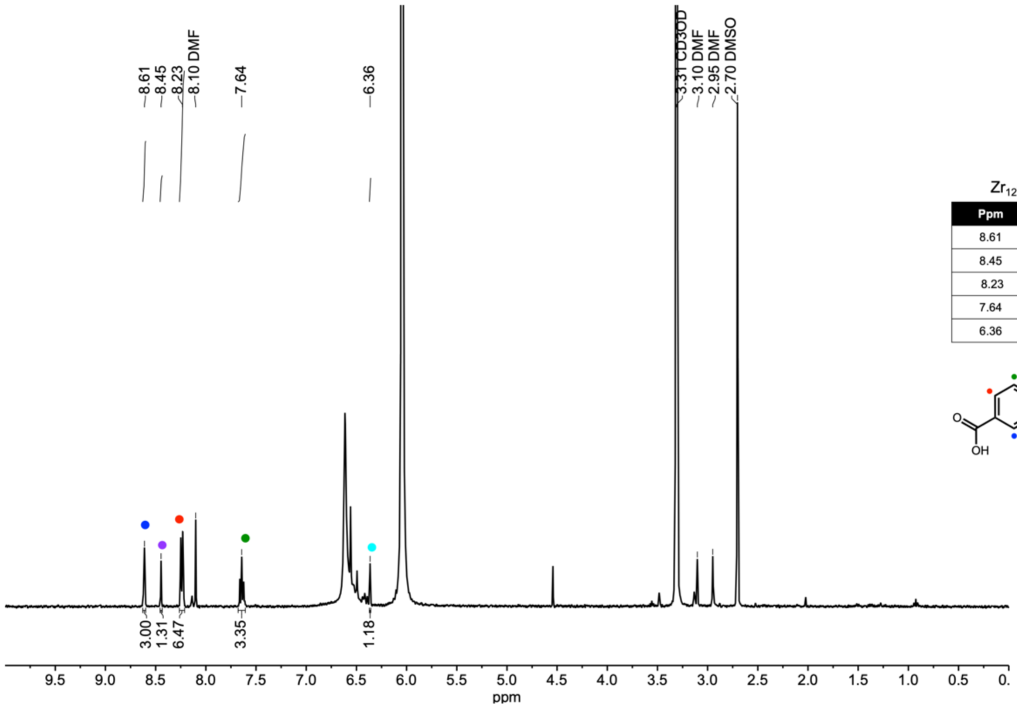


Zr$_{12}$(1,3-bdc)$_3$(*m*-dobdc)$_3$

| Ppm | Assignment | Integration |
|---|---|---|
| 8.61 | ● (blue) | 3.00 |
| 8.45 | ● (purple) | 1.31 |
| 8.23 | ● (red) | 6.47 |
| 7.64 | ● (green) | 3.35 |
| 6.36 | ● (cyan) | 1.18 |

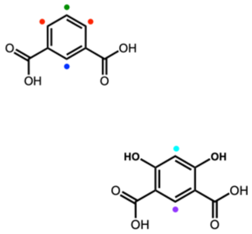


**Figure 22.** $^1$H NMR for the 1,3-bdc: *m*-dobdc heteroleptic cage with a L$^1$:L$^2$ ligand ratio of 3:3 in MeOH-d$_4$.

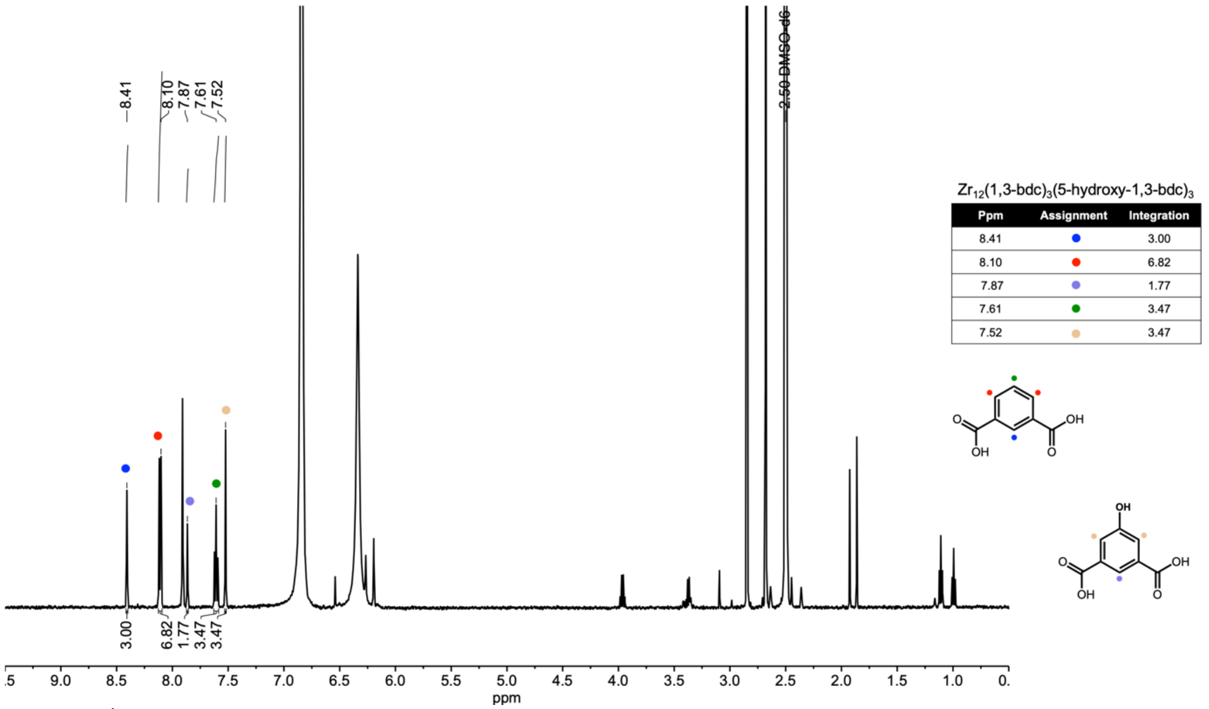


$Zr_{12}(1,3\text{-bdc})_3(5\text{-hydroxy-1,3-bdc})_3$

| Ppm | Assignment | Integration |
|---|---|---|
| 8.41 | ● (blue) | 3.00 |
| 8.10 | ● (red) | 6.82 |
| 7.87 | ● (lavender) | 1.77 |
| 7.61 | ● (green) | 3.47 |
| 7.52 | ● (tan) | 3.47 |

**Figure 23.** $^1$H NMR of the 3:3 1,3-bdc: 5-OH heteroleptic cage in DMSO-$d_6$.

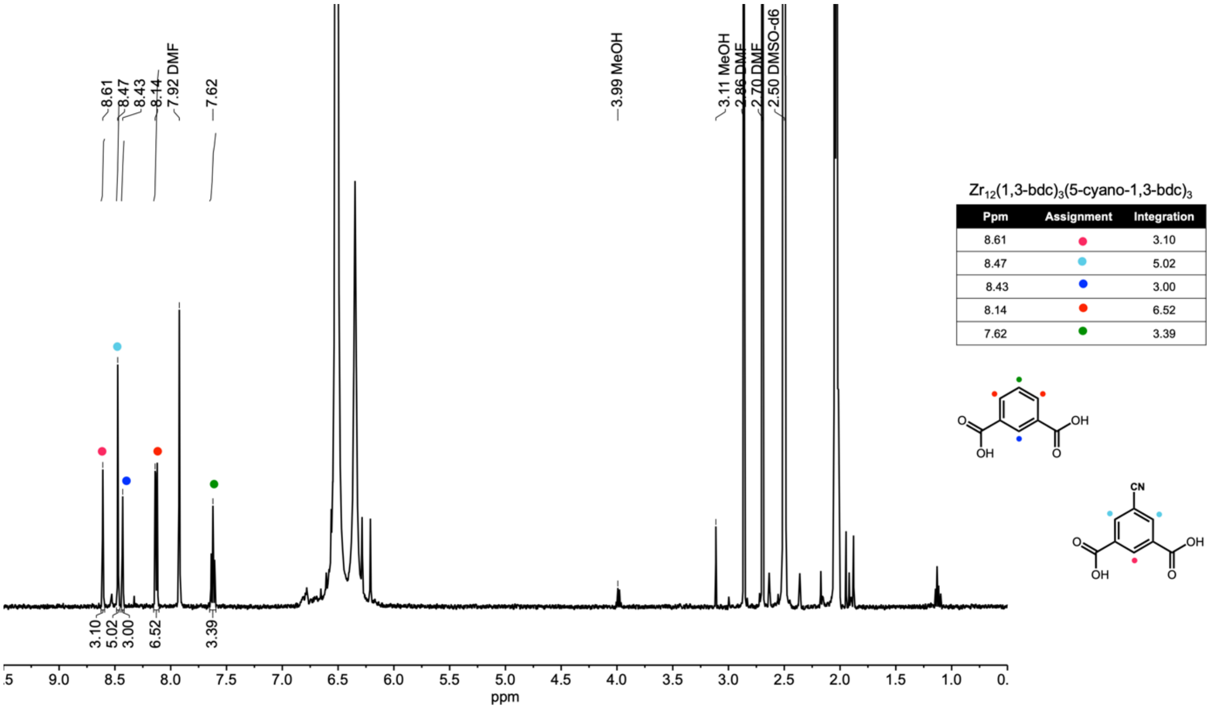


$Zr_{12}(1,3\text{-bdc})_3(5\text{-cyano-1,3-bdc})_3$

| Ppm | Assignment | Integration |
|---|---|---|
| 8.61 | ● (pink) | 3.10 |
| 8.47 | ● (light blue) | 5.02 |
| 8.43 | ● (blue) | 3.00 |
| 8.14 | ● (red) | 6.52 |
| 7.62 | ● (green) | 3.39 |

**Figure 24.** $^1$H NMR of the 3:3 1,3-bdc: 5-CN heteroleptic cage in DMSO-$d_6$.

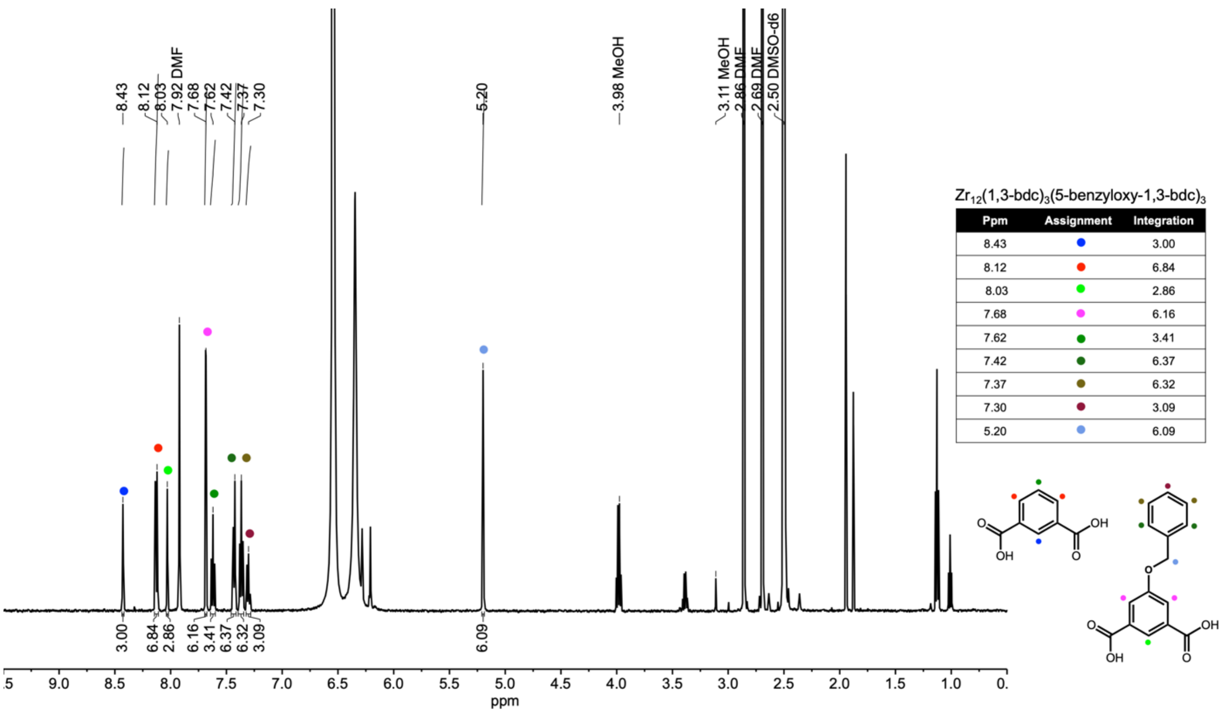


$Zr_{12}$(1,3-bdc)$_3$(5-benzyloxy-1,3-bdc)$_3$

| Ppm | Assignment | Integration |
|---|---|---|
| 8.43 | ● (blue) | 3.00 |
| 8.12 | ● (red) | 6.84 |
| 8.03 | ● (light green) | 2.86 |
| 7.68 | ● (pink) | 6.16 |
| 7.62 | ● (green) | 3.41 |
| 7.42 | ● (dark green) | 6.37 |
| 7.37 | ● (olive) | 6.32 |
| 7.30 | ● (maroon) | 3.09 |
| 5.20 | ● (light blue) | 6.09 |

**Figure 25.** $^1$H NMR of the 3:3 1,3-bdc: 5-benzyloxy heteroleptic cage in DMSO-d$_6$.

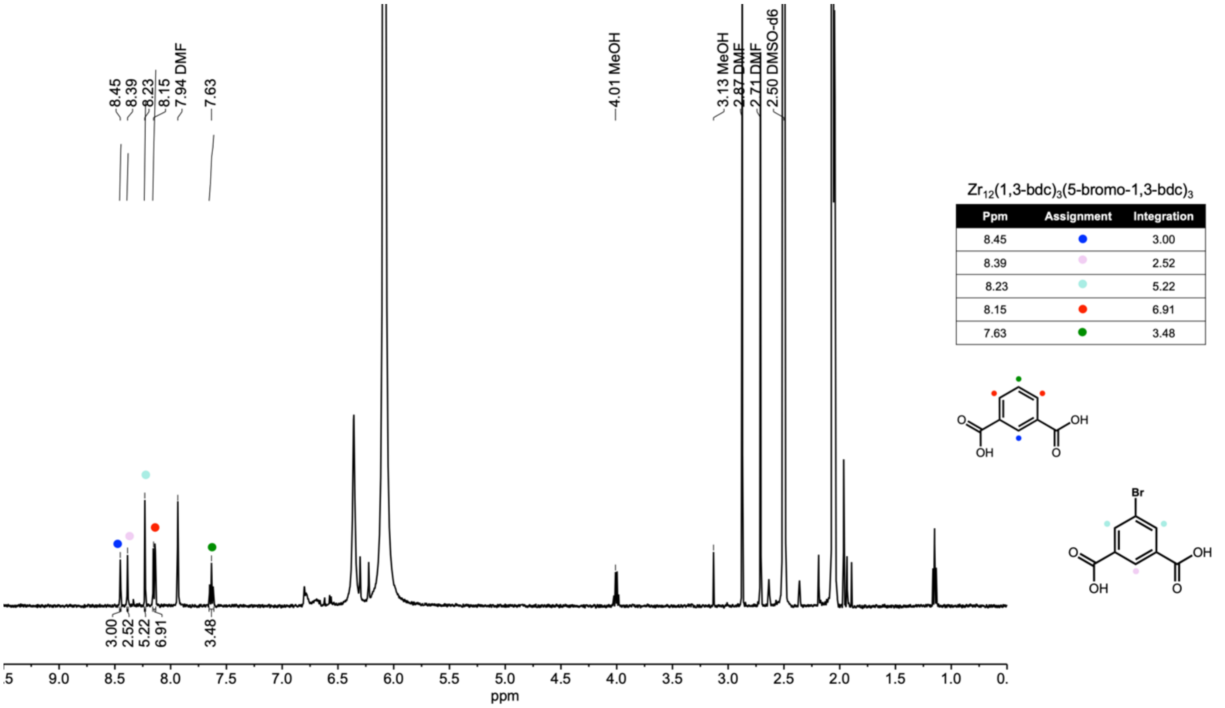


$Zr_{12}$(1,3-bdc)$_3$(5-bromo-1,3-bdc)$_3$

| Ppm | Assignment | Integration |
|---|---|---|
| 8.45 | ● (blue) | 3.00 |
| 8.39 | ● (light pink) | 2.52 |
| 8.23 | ● (light cyan) | 5.22 |
| 8.15 | ● (red) | 6.91 |
| 7.63 | ● (green) | 3.48 |

**Figure 26.** $^1$H NMR of the 3:3 1,3-bdc: 5-Br heteroleptic cage in DMSO-d$_6$

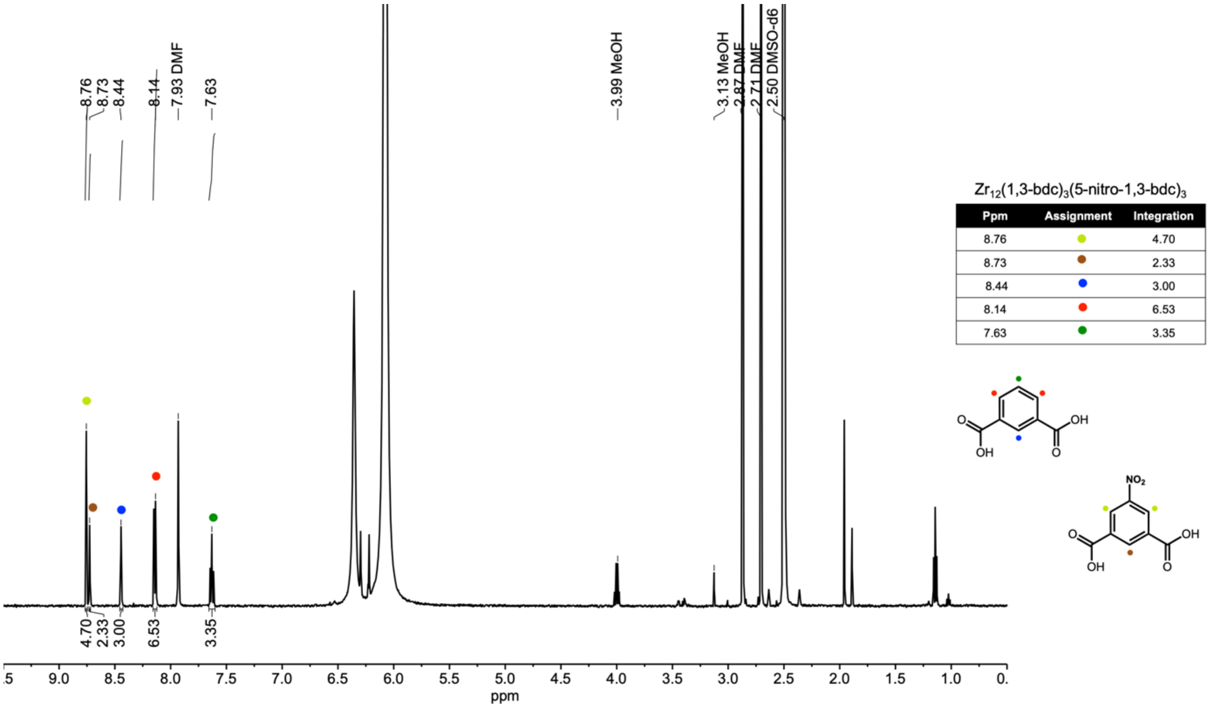


Zr$_{12}$(1,3-bdc)$_3$(5-nitro-1,3-bdc)$_3$

| Ppm | Assignment | Integration |
|---|---|---|
| 8.76 | ● (yellow-green) | 4.70 |
| 8.73 | ● (brown) | 2.33 |
| 8.44 | ● (blue) | 3.00 |
| 8.14 | ● (red) | 6.53 |
| 7.63 | ● (green) | 3.35 |

**Figure 27.** $^1$H NMR of the 3:3 1,3-bdc: 5-$NO_2$ heteroleptic cage in DMSO-d$_6$.

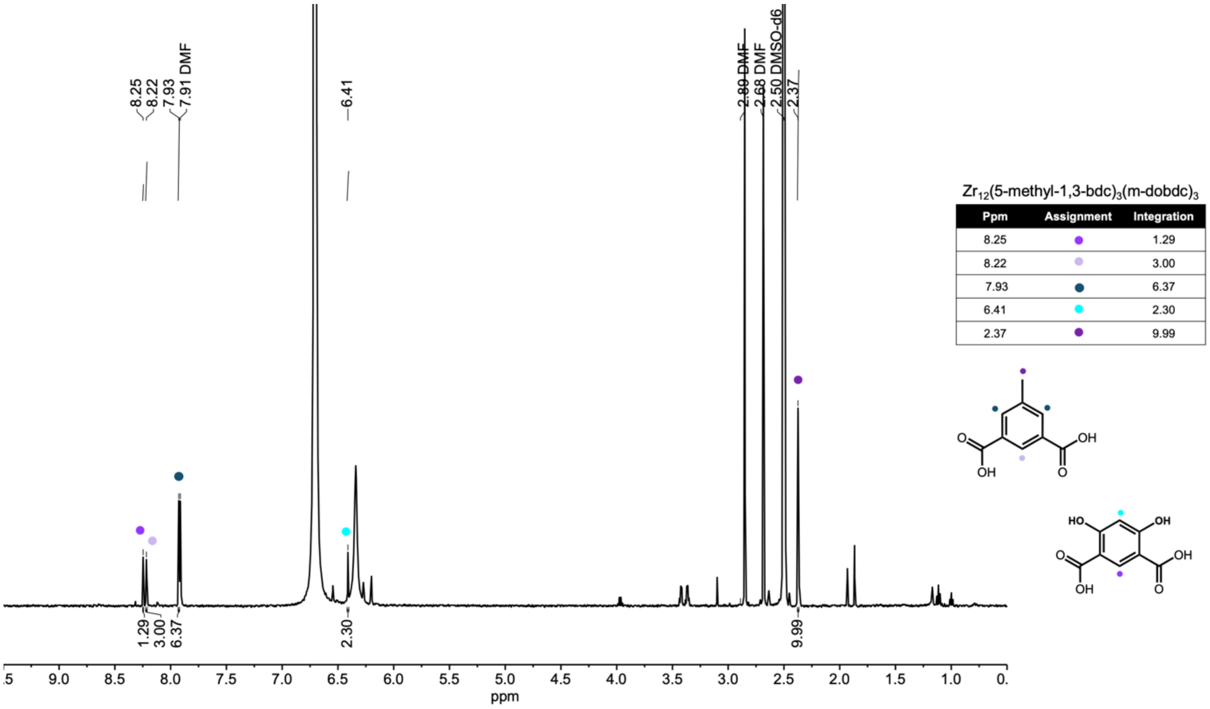


Zr$_{12}$(5-methyl-1,3-bdc)$_3$(m-dobdc)$_3$

| Ppm | Assignment | Integration |
|---|---|---|
| 8.25 | ● (purple) | 1.29 |
| 8.22 | ● (lavender) | 3.00 |
| 7.93 | ● (dark teal) | 6.37 |
| 6.41 | ● (cyan) | 2.30 |
| 2.37 | ● (dark purple) | 9.99 |

**Figure 28.** $^1$H NMR of the 3:3 5-methyl: *m*-dobdc heteroleptic cage in DMSO-d$_6$.

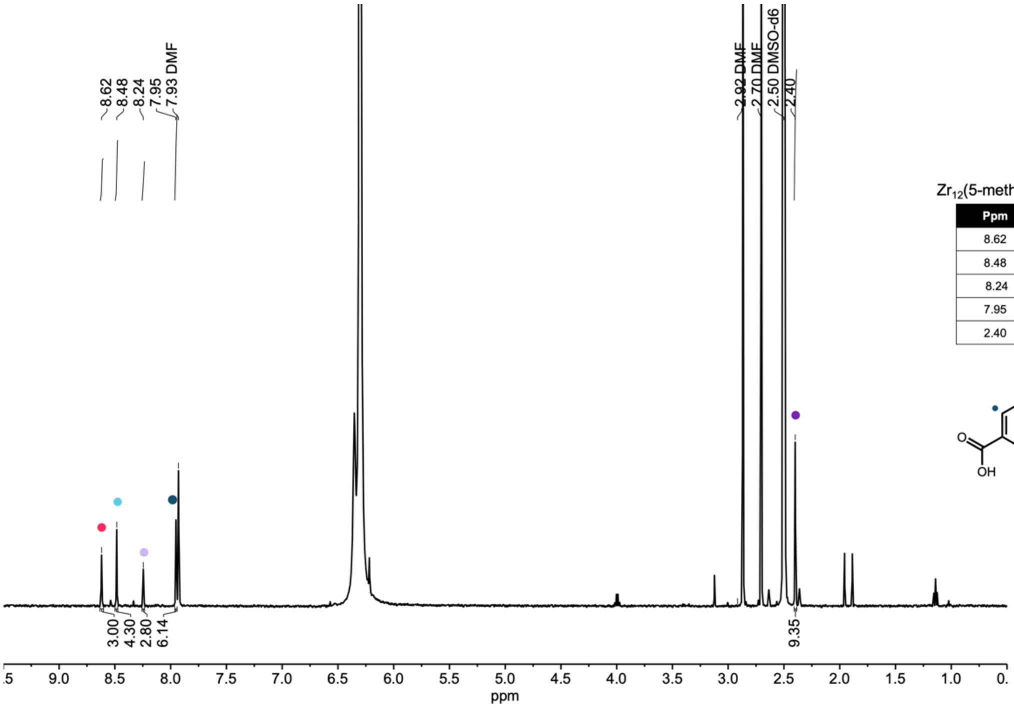


Zr$_{12}$(5-methyl-1,3-bdc)$_3$(5-cyano-1,3-bdc)$_3$

| Ppm | Assignment | Integration |
|---|---|---|
| 8.62 | ● (red) | 3.00 |
| 8.48 | ● (cyan) | 4.30 |
| 8.24 | ● (lavender) | 2.80 |
| 7.95 | ● (dark blue) | 6.14 |
| 2.40 | ● (purple) | 9.35 |

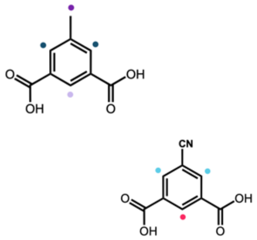


**Figure 29.** $^1$H NMR of the 3:3 5-methyl: 5-CN heteroleptic cage in DMSO-d$_6$.

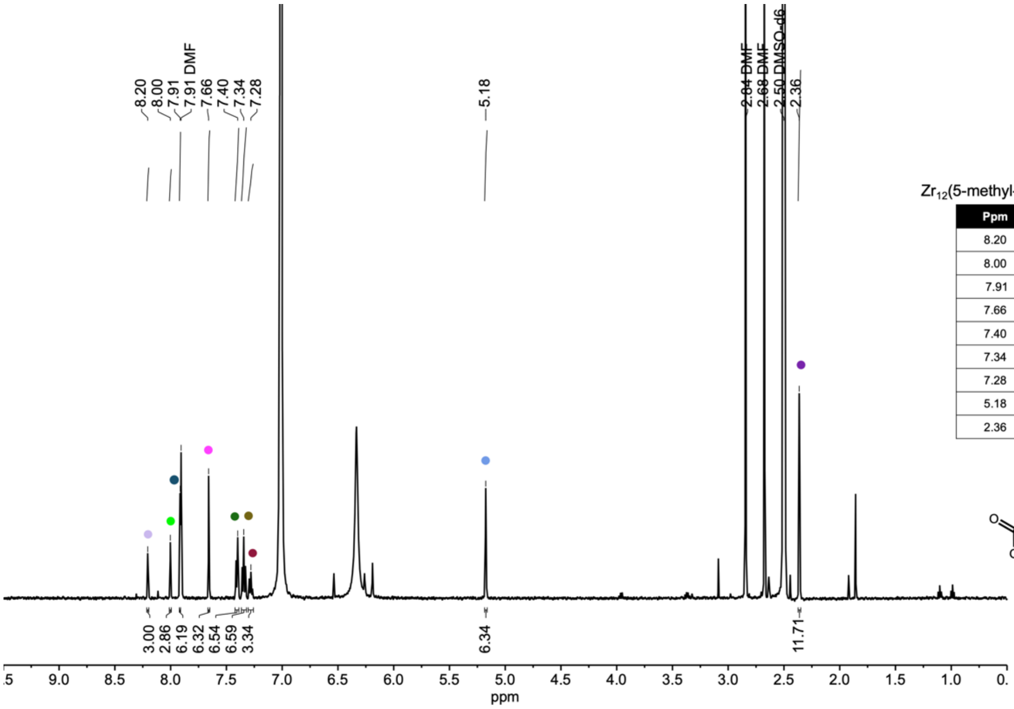


Zr$_{12}$(5-methyl-1,3-bdc)$_3$(5-benzyloxy-1,3-bdc)$_3$

| Ppm | Assignment | Integration |
|---|---|---|
| 8.20 | ● (lavender) | 3.00 |
| 8.00 | ● (green) | 2.86 |
| 7.91 | ● (dark blue) | 6.19 |
| 7.66 | ● (magenta) | 6.32 |
| 7.40 | ● (dark green) | 6.54 |
| 7.34 | ● (olive) | 6.59 |
| 7.28 | ● (maroon) | 3.34 |
| 5.18 | ● (light blue) | 6.34 |
| 2.36 | ● (purple) | 11.71 |

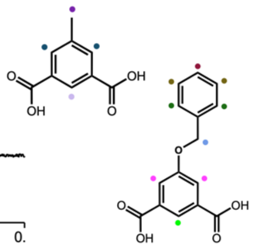


**Figure 30.** $^1$H NMR of the 3:3 5-methyl: 5-benzyloxy heteroleptic cage in DMSO-d$_6$.

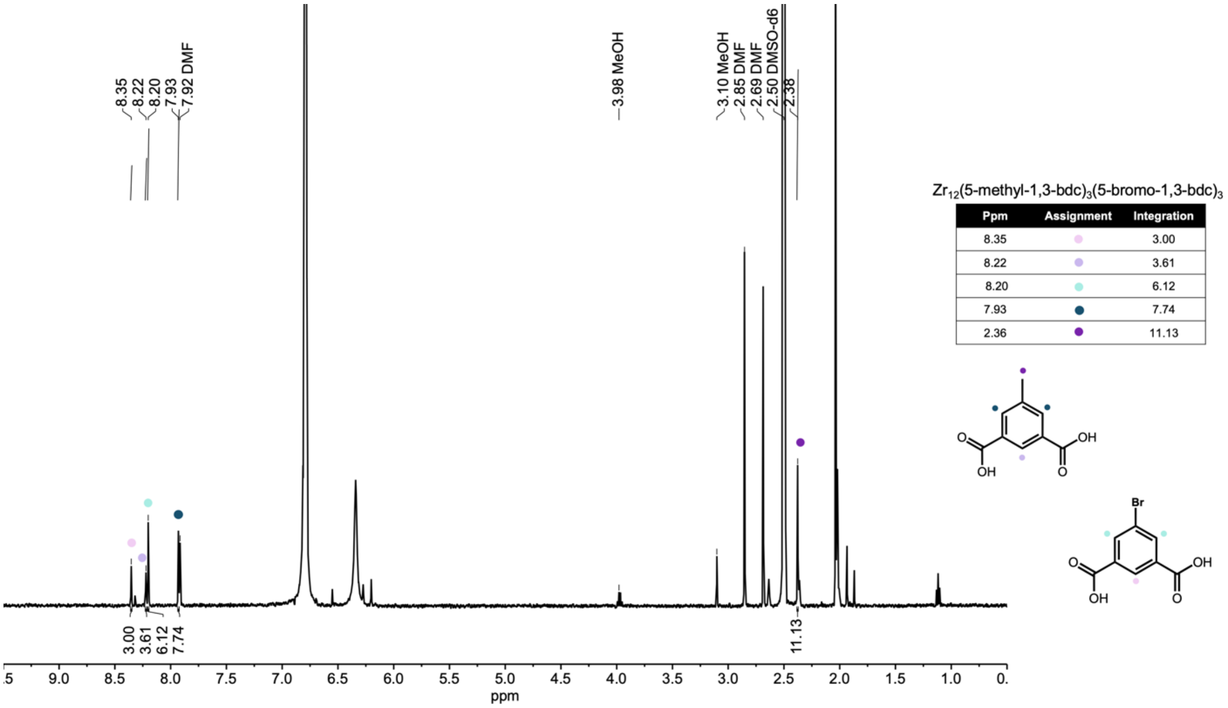


Zr$_{12}$(5-methyl-1,3-bdc)$_3$(5-bromo-1,3-bdc)$_3$

| Ppm | Assignment | Integration |
|---|---|---|
| 8.35 | ● | 3.00 |
| 8.22 | ● | 3.61 |
| 8.20 | ● | 6.12 |
| 7.93 | ● | 7.74 |
| 2.36 | ● | 11.13 |

**Figure 31.** $^{1}$H NMR of the 3:3 5-methyl: 5-Br heteroleptic cage in DMSO-d$_6$.

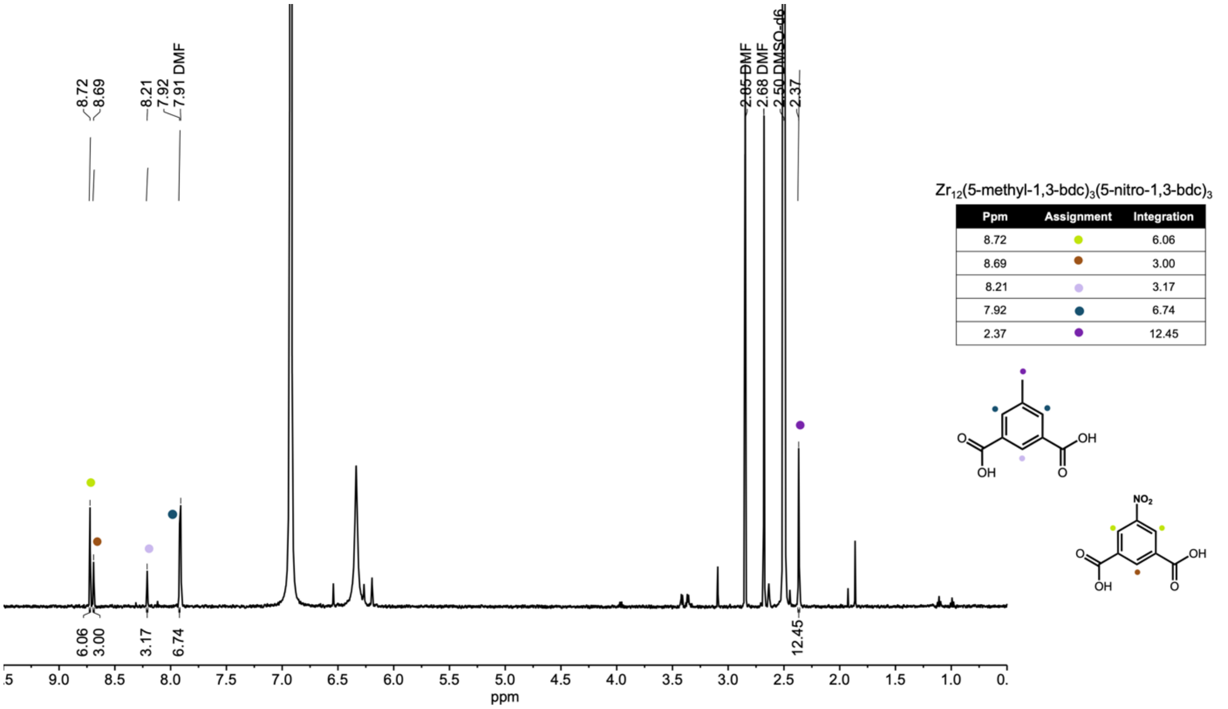


Zr$_{12}$(5-methyl-1,3-bdc)$_3$(5-nitro-1,3-bdc)$_3$

| Ppm | Assignment | Integration |
|---|---|---|
| 8.72 | ● | 6.06 |
| 8.69 | ● | 3.00 |
| 8.21 | ● | 3.17 |
| 7.92 | ● | 6.74 |
| 2.37 | ● | 12.45 |

**Figure 32.** $^{1}$H NMR of the 3:3 5-methyl: 5-NO$_2$ heteroleptic cage in DMSO-d$_6$.

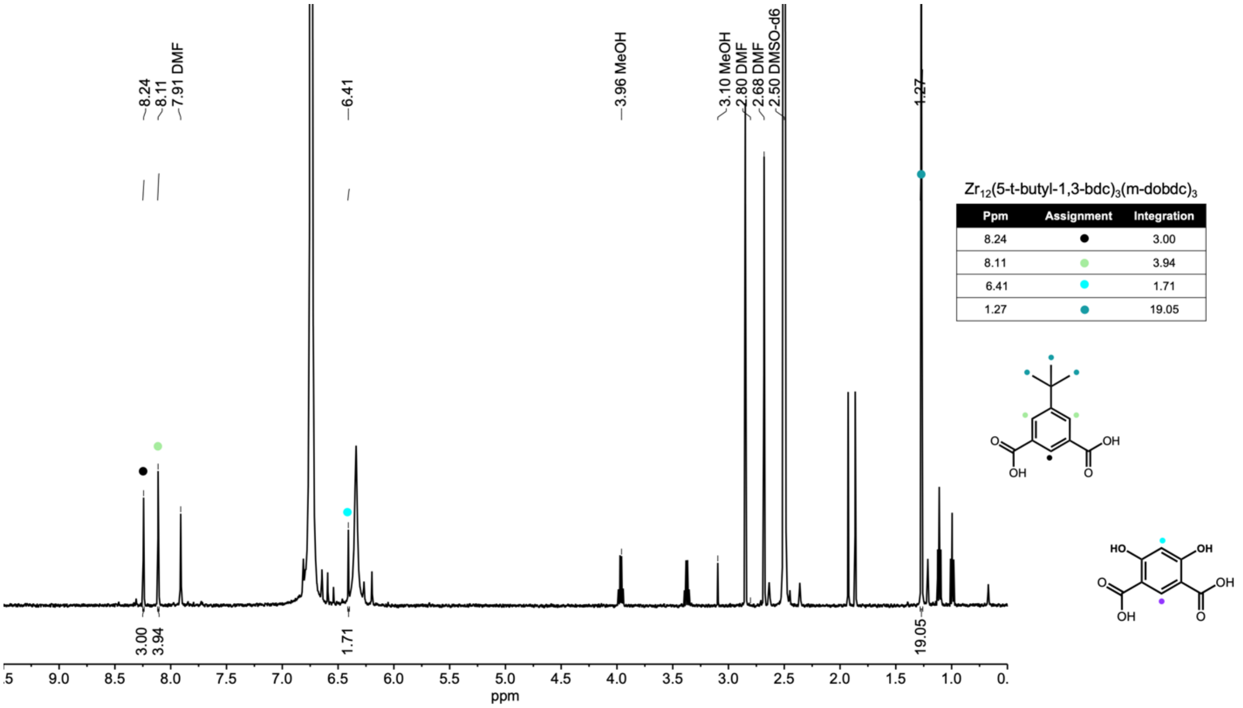


**Figure 33.** $^{1}$H NMR of the 3:3 5-*tert*-butyl: *m*-dobdc heteroleptic cage in DMSO-$d_6$.

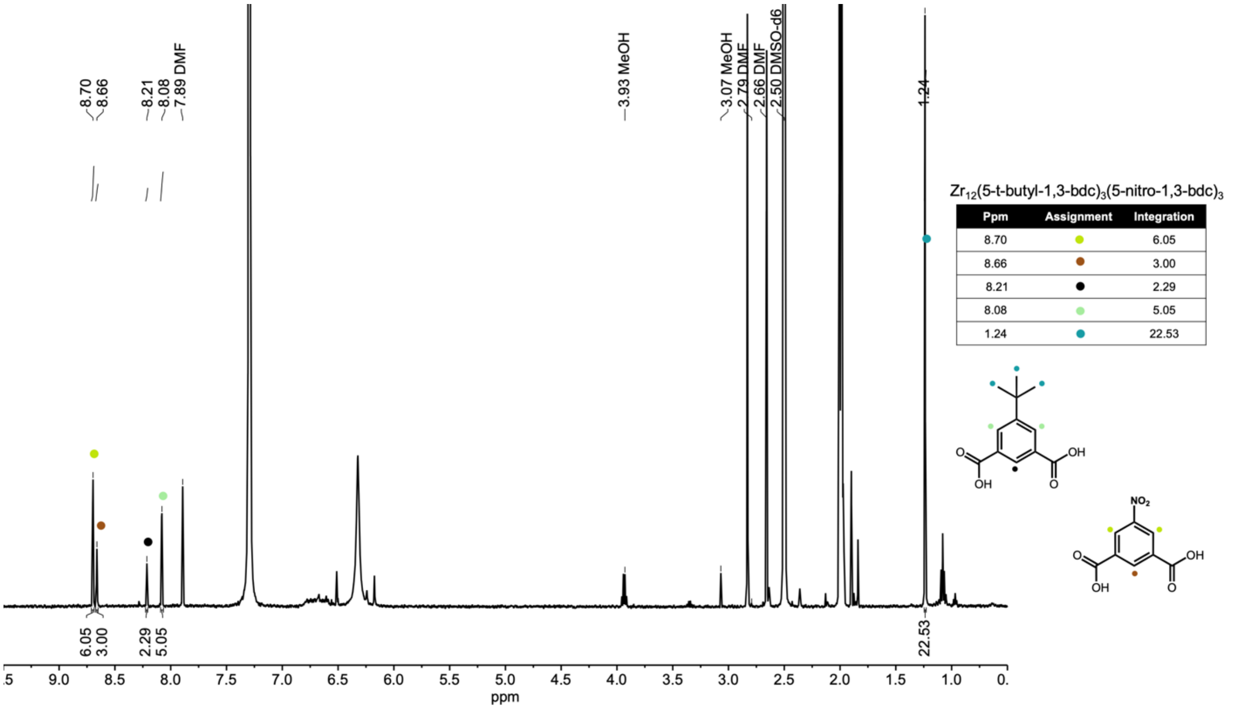


**Figure 34.** $^{1}$H NMR of the 3:3 5-*tert*-butyl: 5-$NO_2$ heteroleptic cage in DMSO-$d_6$.

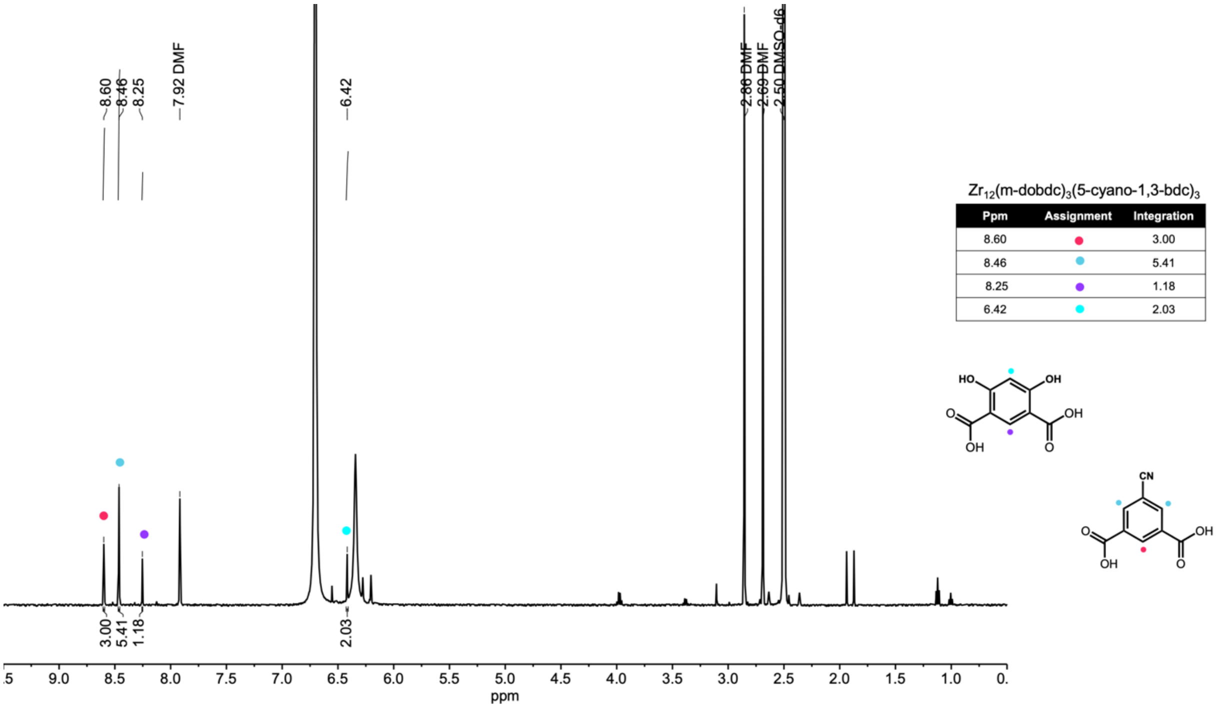


$Zr_{12}$(m-dobdc)$_3$(5-cyano-1,3-bdc)$_3$

| Ppm | Assignment | Integration |
|---|---|---|
| 8.60 | ● | 3.00 |
| 8.46 | ● | 5.41 |
| 8.25 | ● | 1.18 |
| 6.42 | ● | 2.03 |

**Figure 35.** $^1$H NMR of the 3:3 *m*-dobdc: 5-CN heteroleptic cage in DMSO-d$_6$.

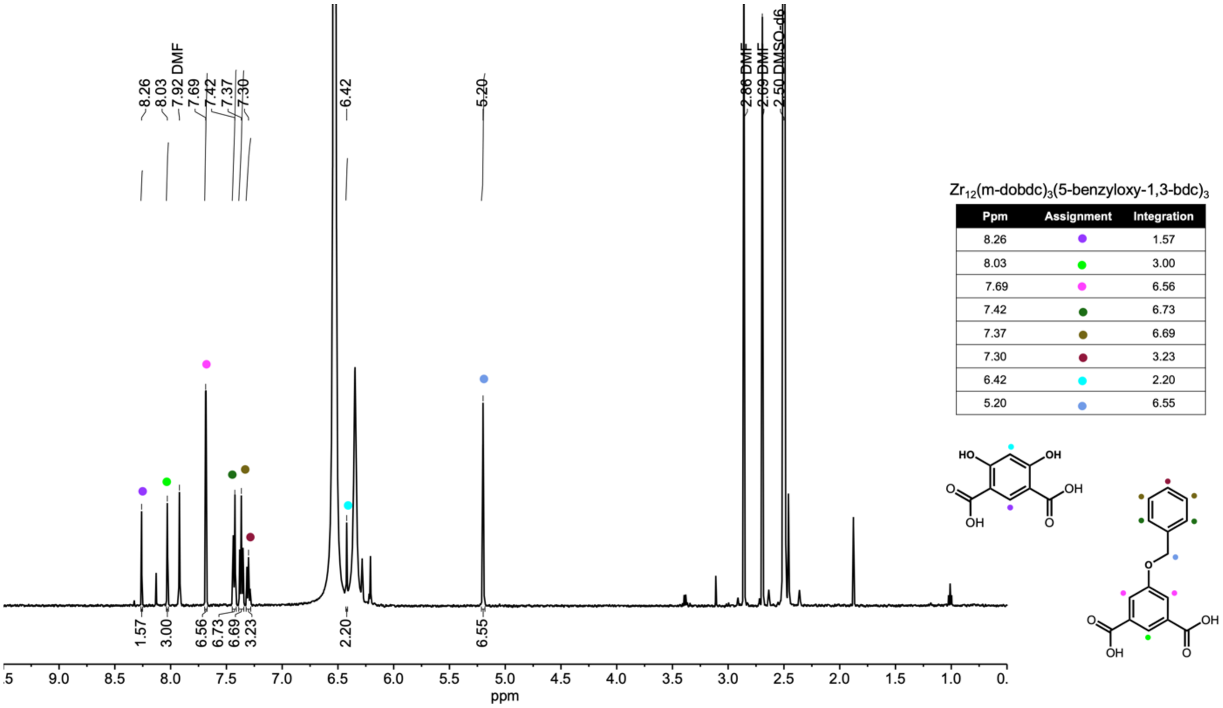


$Zr_{12}$(m-dobdc)$_3$(5-benzyloxy-1,3-bdc)$_3$

| Ppm | Assignment | Integration |
|---|---|---|
| 8.26 | ● | 1.57 |
| 8.03 | ● | 3.00 |
| 7.69 | ● | 6.56 |
| 7.42 | ● | 6.73 |
| 7.37 | ● | 6.69 |
| 7.30 | ● | 3.23 |
| 6.42 | ● | 2.20 |
| 5.20 | ● | 6.55 |

**Figure 36.** $^1$H NMR of the 3:3 *m*-dobdc: 5-benzyloxy heteroleptic cage in DMSO-d$_6$.

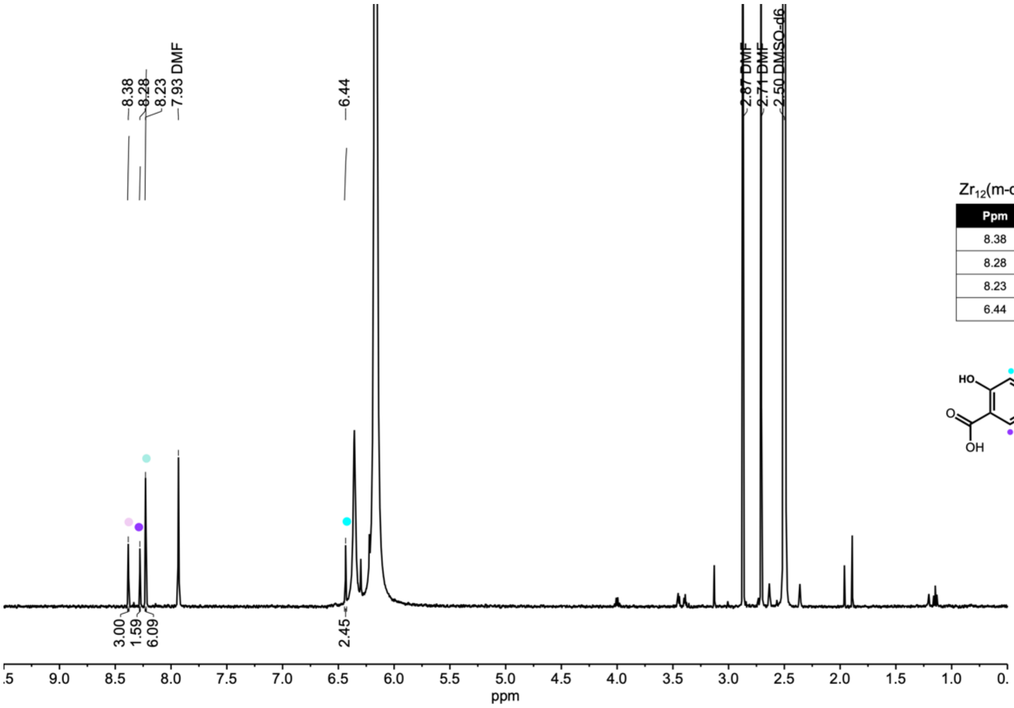


$Zr_{12}(m\text{-dobdc})_3(5\text{-bromo-1,3-bdc})_3$

| Ppm | Assignment | Integration |
|---|---|---|
| 8.38 | ● | 3.00 |
| 8.28 | ● | 1.59 |
| 8.23 | ● | 6.09 |
| 6.44 | ● | 2.45 |

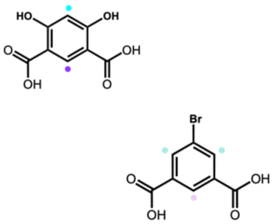


**Figure 37.** $^{1}$H NMR of the 3:3 *m*-dobdc: 5-Br heteroleptic cage in DMSO-d$_6$.

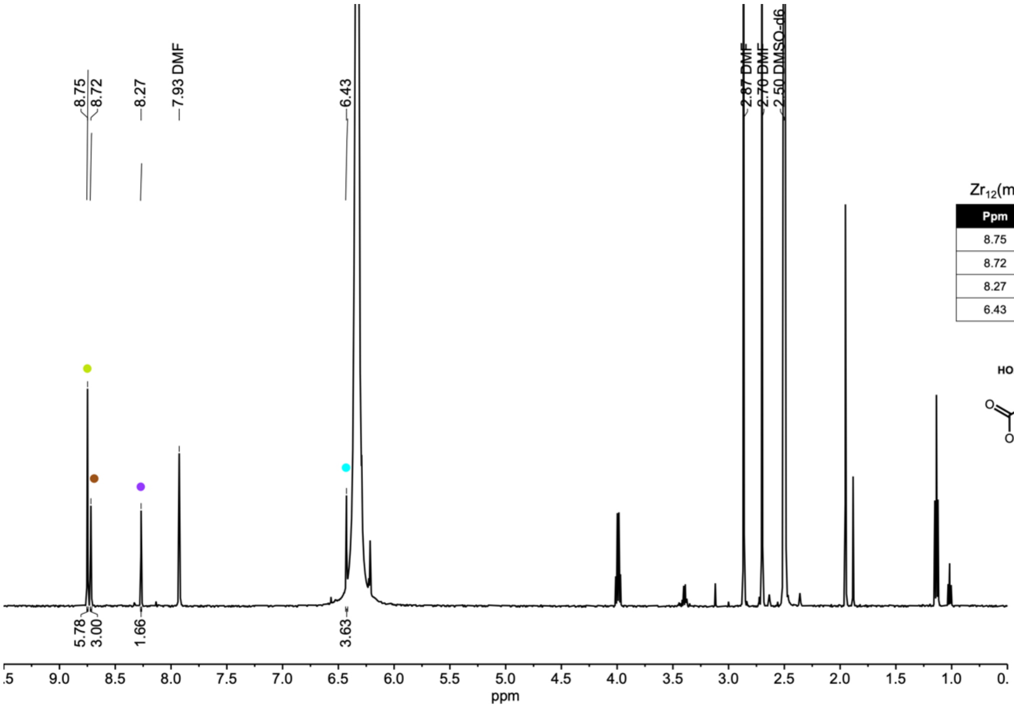


$Zr_{12}(m\text{-dobdc})_3(5\text{-nitro-1,3-bdc})_3$

| Ppm | Assignment | Integration |
|---|---|---|
| 8.75 | ● | 5.78 |
| 8.72 | ● | 3.00 |
| 8.27 | ● | 1.66 |
| 6.43 | ● | 3.63 |

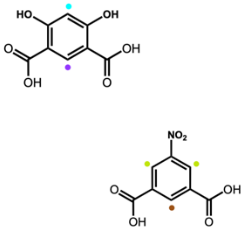


**Figure 38.** $^{1}$H NMR of the 3:3 *m*-dobdc: 5-$NO_2$ heteroleptic cage in DMSO-d$_6$.

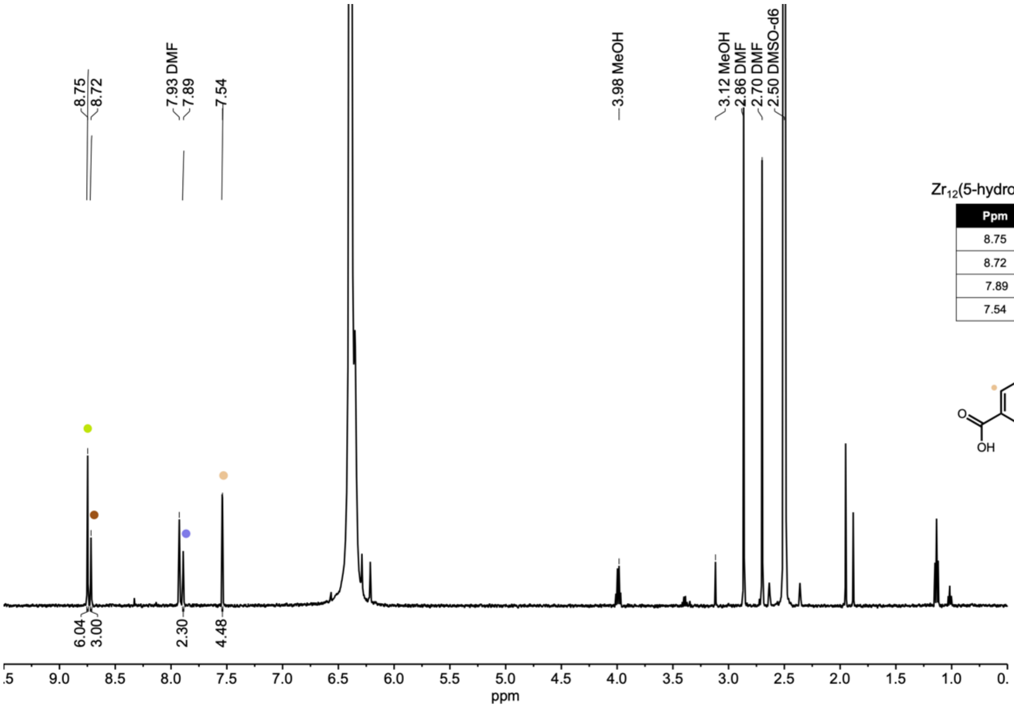


$Zr_{12}$(5-hydroxy-1,3-bdc)$_3$(5-nitro-1,3-bdc)$_3$

| Ppm | Assignment | Integration |
|---|---|---|
| 8.75 | ● | 6.04 |
| 8.72 | ● | 3.00 |
| 7.89 | ● | 2.30 |
| 7.54 | ● | 4.48 |

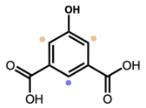


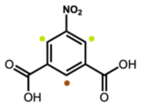


**Figure 39.** $^{1}$H NMR of the 3:3 5-OH: 5-$NO_2$ heteroleptic cage in DMSO-$d_6$.

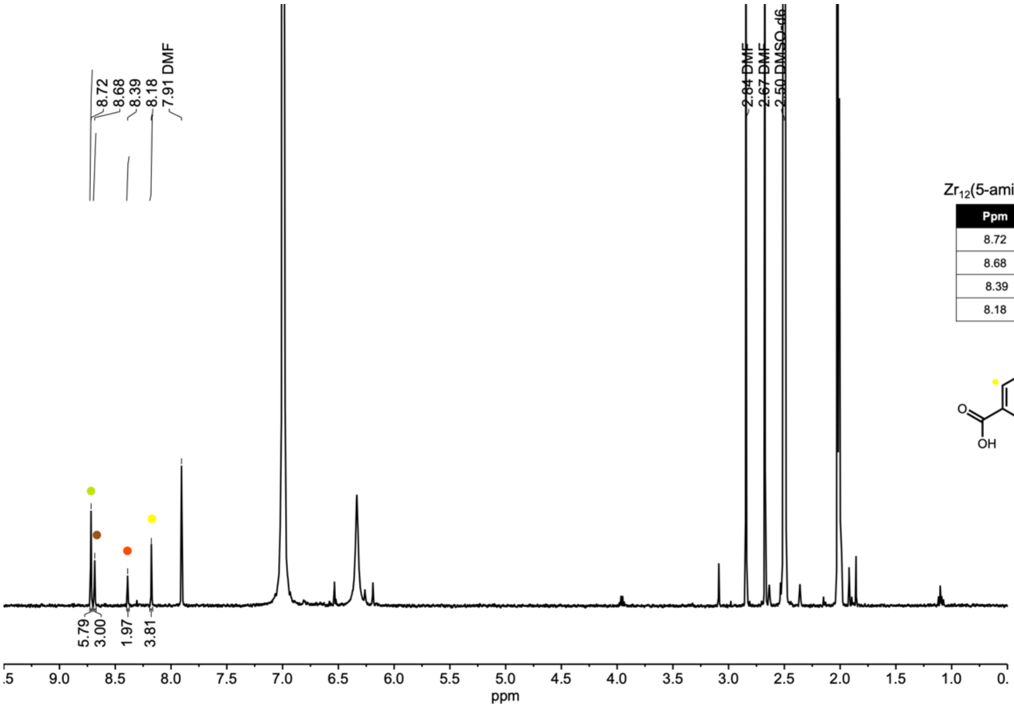


$Zr_{12}$(5-amino-1,3-bdc)$_3$(5-nitro-1,3-bdc)$_3$

| Ppm | Assignment | Integration |
|---|---|---|
| 8.72 | ● | 5.79 |
| 8.68 | ● | 3.00 |
| 8.39 | ● | 2.30 |
| 8.18 | ● | 4.48 |

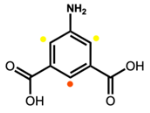


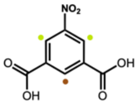


**Figure 40.** $^{1}$H NMR of the 3:3 5-$NH_2$: 5-$NO_2$ heteroleptic cage in DMSO-$d_6$.

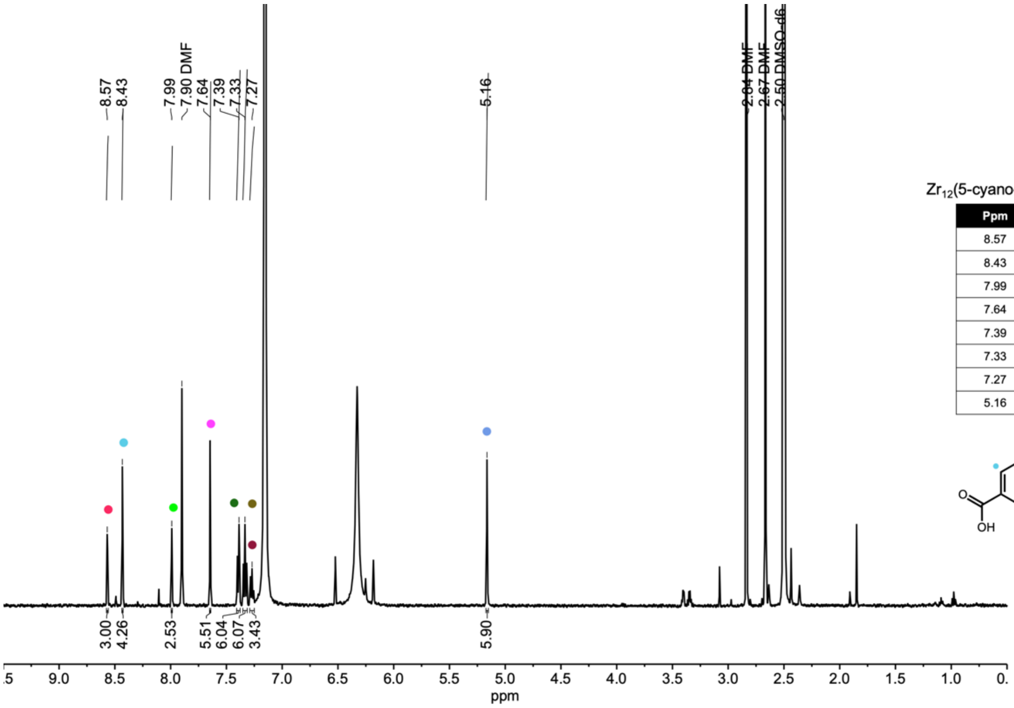


Zr$_{12}$(5-cyano-1,3-bdc)$_3$(5-benzyloxy-1,3-bdc)$_3$

| Ppm | Assignment | Integration |
|---|---|---|
| 8.57 | ● (red) | 3.00 |
| 8.43 | ● (light blue) | 4.26 |
| 7.99 | ● (green) | 2.53 |
| 7.64 | ● (magenta) | 5.51 |
| 7.39 | ● (dark green) | 6.04 |
| 7.33 | ● (olive) | 6.07 |
| 7.27 | ● (maroon) | 3.43 |
| 5.16 | ● (blue) | 5.90 |

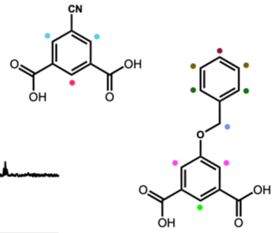


**Figure 41.** $^1$H NMR of the 3:3 5-CN: 5-benzyloxy heteroleptic cage in DMSO-d$_6$.

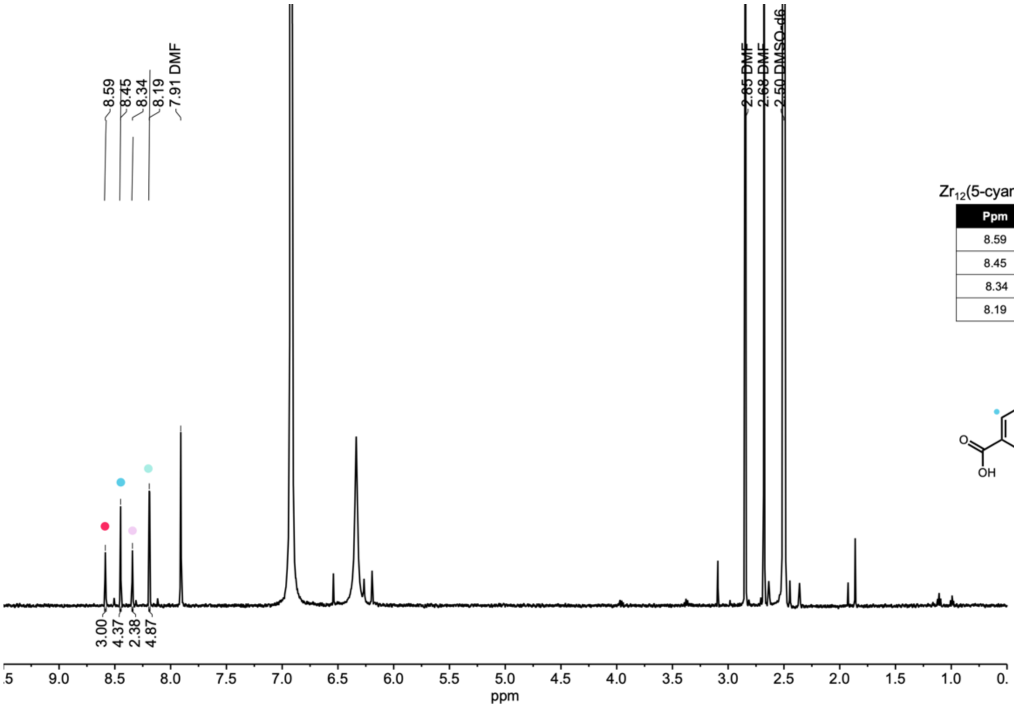


Zr$_{12}$(5-cyano-1,3-bdc)$_3$(5-bromo-1,3-bdc)$_3$

| Ppm | Assignment | Integration |
|---|---|---|
| 8.59 | ● (red) | 3.00 |
| 8.45 | ● (light blue) | 4.37 |
| 8.34 | ● (pale pink) | 2.38 |
| 8.19 | ● (pale cyan) | 4.87 |

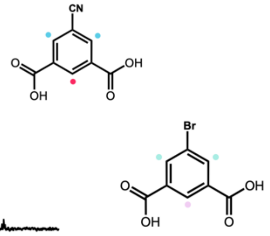


**Figure 42.** $^1$H NMR of the 3:3 5-CN: 5-Br heteroleptic cage in DMSO-d$_6$.

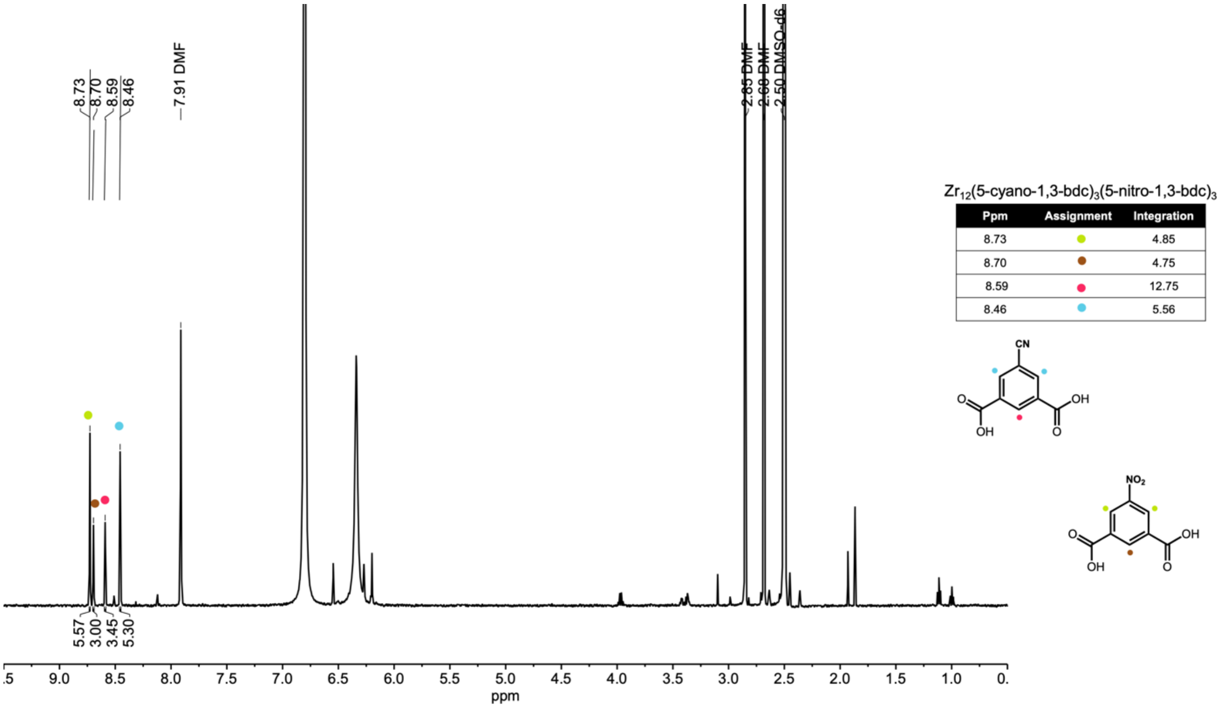


$Zr_{12}$(5-cyano-1,3-bdc)$_3$(5-nitro-1,3-bdc)$_3$

| Ppm | Assignment | Integration |
|---|---|---|
| 8.73 | ● | 4.85 |
| 8.70 | ● | 4.75 |
| 8.59 | ● | 12.75 |
| 8.46 | ● | 5.56 |

**Figure 43.** $^1$H NMR of the 3:3 5-CN: 5-$NO_2$ heteroleptic cage in DMSO-$d_6$.

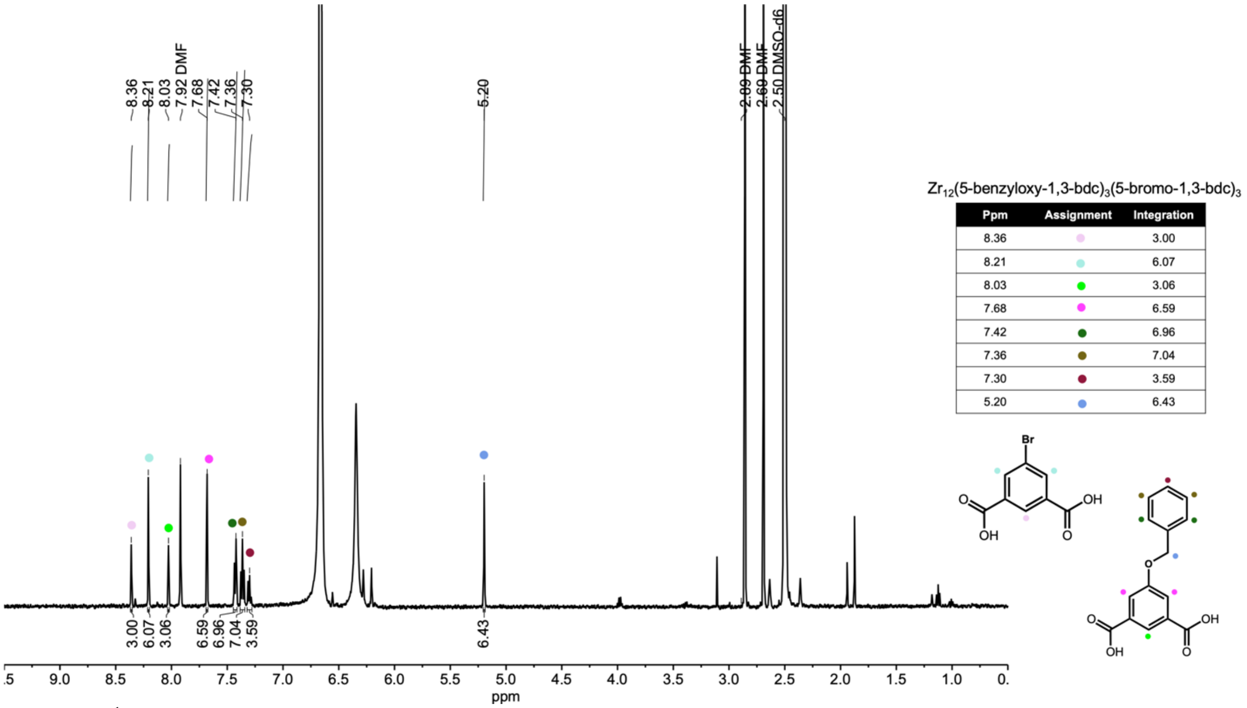


$Zr_{12}$(5-benzyloxy-1,3-bdc)$_3$(5-bromo-1,3-bdc)$_3$

| Ppm | Assignment | Integration |
|---|---|---|
| 8.36 | ● | 3.00 |
| 8.21 | ● | 6.07 |
| 8.03 | ● | 3.06 |
| 7.68 | ● | 6.59 |
| 7.42 | ● | 6.96 |
| 7.36 | ● | 7.04 |
| 7.30 | ● | 3.59 |
| 5.20 | ● | 6.43 |

**Figure 44.** $^1$H NMR of the 3:3 5-benzyloxy: 5-Br heteroleptic cage in DMSO-$d_6$.

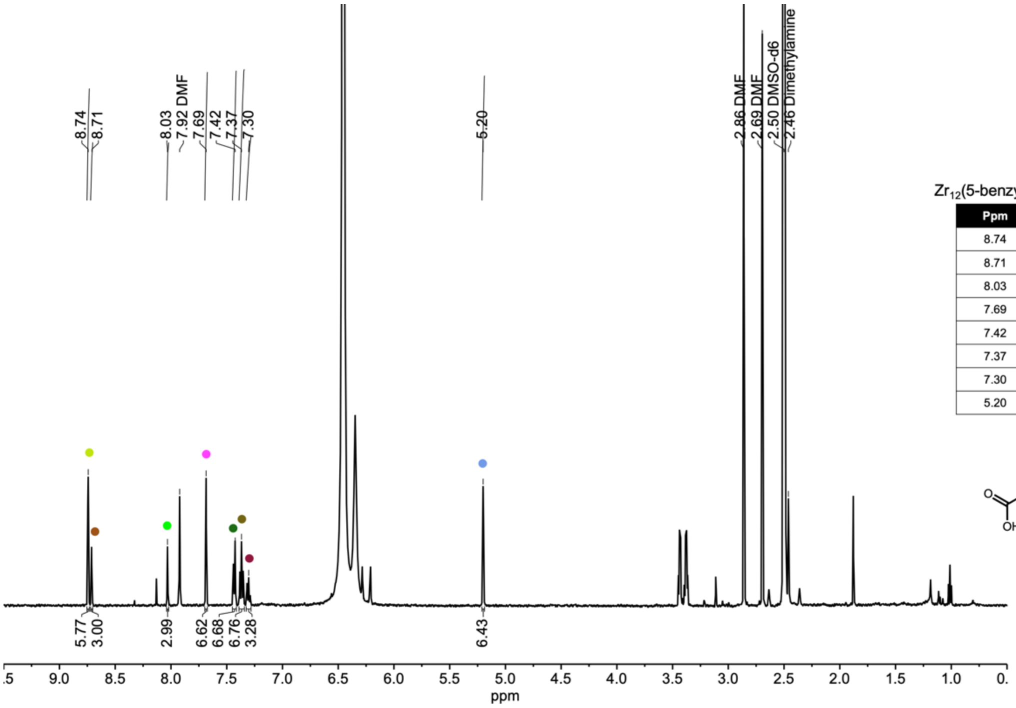


$Zr_{12}$(5-benzyloxy-1,3-bdc)$_3$(5-nitro-1,3-bdc)$_3$

| Ppm | Assignment | Integration |
|---|---|---|
| 8.74 | ● | 5.77 |
| 8.71 | ● | 3.00 |
| 8.03 | ● | 2.99 |
| 7.69 | ● | 6.62 |
| 7.42 | ● | 6.68 |
| 7.37 | ● | 6.76 |
| 7.30 | ● | 3.28 |
| 5.20 | ● | 6.43 |

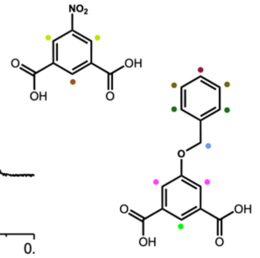


**Figure 45.** $^1$H NMR of the 3:3 5-benzyloxy: 5-$NO_2$ heteroleptic cage in DMSO-d$_6$.

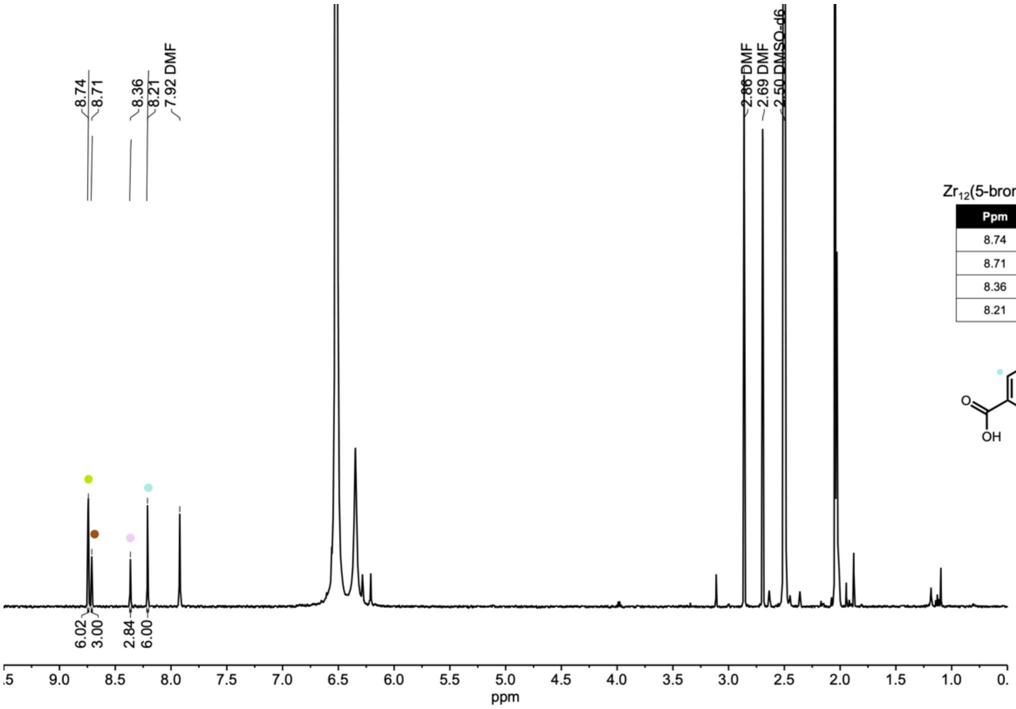


$Zr_{12}$(5-bromo-1,3-bdc)$_3$(5-nitro-1,3-bdc)$_3$

| Ppm | Assignment | Integration |
|---|---|---|
| 8.74 | ● | 6.02 |
| 8.71 | ● | 3.00 |
| 8.36 | ● | 2.84 |
| 8.21 | ● | 6.00 |

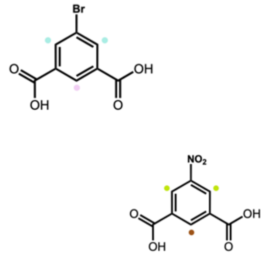


**Figure 46.** $^1$H NMR of the 3:3 5-Br: 5-$NO_2$ heteroleptic cage in DMSO-d$_6$.

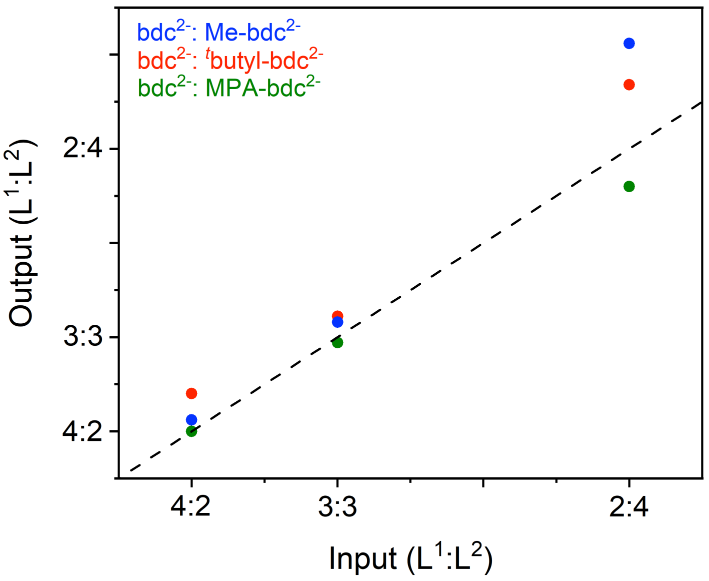


**Figure 47.** Use of the digested cage NMR peak integrations where ratios of ligands put into the reaction were compared to ligand ratios from the spectra where $L^1$ is 1,3-bdc while $L^2$ is either 5-methyl$^{2-}$ (blue), 5-*tert*-butyl$^{2-}$ (red), or 5-MPA$^{2-}$ (green) and are compared to the expected ligand ratios.

## Powder X-Ray Diffraction (PXRD)

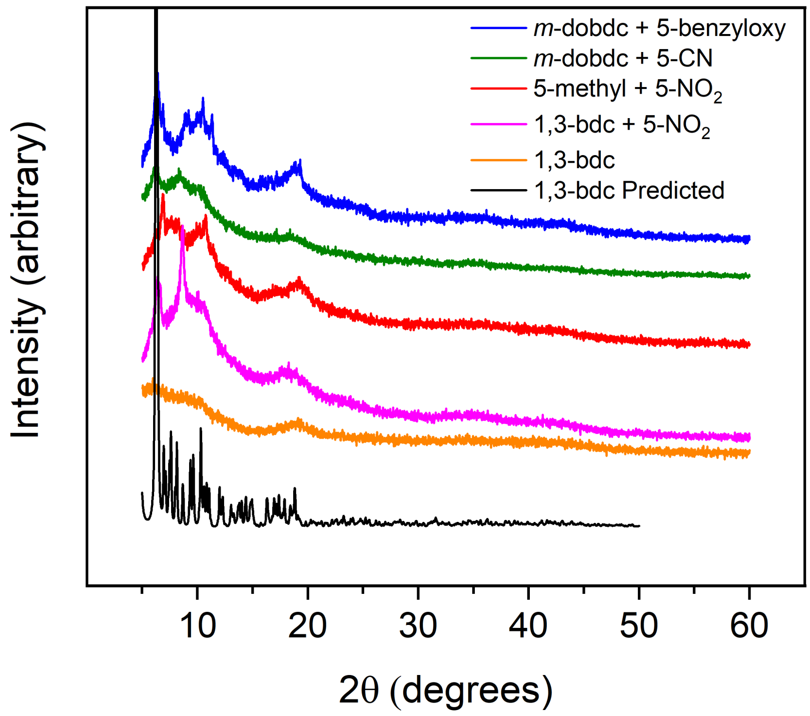


**Figure 48.** PXRD patterns for 3:3 heteroleptic cages *m*-dobdc:5-benzyloxy (blue), *m*-dobdc:5-CN (green), 5-methyl:5-$NO_2$ (red), 1,3-bdc:5-$NO_2$ (pink) and the homoleptic 1,3-bdc cage (orange). The predicted pattern has been generated through Mercury using a previously reported data set.[5]

## Liquid Chromatography- Mass Spectrometry (LC-MS)

**Table S2. Theoretical and experimental results for *M/Z* ratios for 1,3-bdc: 5-methyl heteroleptic cages.**

| Ratio of 1,3-bdc : 5-methyl | Value | *M/Z* 4+ | *M/Z* 3+ | *M/Z* 2+ |
|---|---|---|---|---|
| 4:2 | Theoretical | 789.2 | 1052 | 1577 |
| | Experimental | 788.8 | 1051 | 1578 |
| 3:3 | Theoretical | 792.7 | 1057 | 1584 |
| | Experimental | 792.8 | 1056 | 1583 |
| 2:4 | Theoretical | 796.2 | 1061 | 1591 |
| | Experimental | 795.8 | 1061 | 1591 |
| 1:5 | Theoretical | 799.7 | 1066 | 1598 |
| | Experimental | 799.4 | 1065 | 1597 |

**Table S3. Theoretical and experimental results for *M/Z* ratios for 1,3-bdc: 5-*tert*-butyl heteroleptic cages.**

| Ratio of 1,3-bdc : 5-*tert*-butyl | Value | *M/Z* 4+ | *M/Z* 3+ | *M/Z* 2+ |
|---|---|---|---|---|
| 4:2 | Theoretical | 810.2 | 1080 | 1619 |
| | Experimental | 809.8 | 1080 | 1619 |
| 3:3 | Theoretical | 824.2 | 1099 | 1647 |
| | Experimental | 824.1 | 1098 | 1647 |
| 2:4 | Theoretical | 838.3 | 1117 | 1676 |
| | Experimental | 838.4 | 1117 | 1675 |

**Table S4. Theoretical and experimental results for *M/Z* ratios for 1,3-bdc: 5-MPA heteroleptic cages.**

| Ratio of 1,3-bdc : 5-MPA | Value | *M/Z* 4+ | *M/Z* 3+ | *M/Z* 2+ |
|---|---|---|---|---|
| 5:1 | Theoretical | 806.9 | 1076 | 1613 |
| | Experimental | 806.8 | 1076 | 1612 |
| 4:2 | Theoretical | 831.7 | 1108 | 1662 |
| | Experimental | 831.4 | 1109 | 1663 |
| 3:3 | Theoretical | 856.5 | 1141 | 1712 |
| | Experimental | 856.4 | 1142 | 1712 |
| 2:4 | Theoretical | 881.3 | 1174 | 1762 |
| | Experimental | 881.4 | 1175 | 1762 |

**Table S5. Theoretical and experimental results for *M/Z* ratios for 1,3-bdc: *m*-dobdc heteroleptic cages.**

| Ratio of 1,3-bdc : *m*-dobdc | Value | *M/Z* 4+ | *M/Z* 3+ | *M/Z* 2+ |
|---|---|---|---|---|
| 5:1 | Theoretical | 790.2 | 1053 | 1579 |
| | Experimental | 781.8 | 1042 | 1563 |
| 4:2 | Theoretical | 798.2 | 1064 | 1595 |
| | Experimental | 781.8 | 1042 | 1563 |
| 3:3 | Theoretical | 806.1 | 1075 | 1611 |
| | Experimental | 789.4 | 1053 | 1579 |

<u>Example Calculations for LC-MS M/Z Peaks</u>

Basket ($Zr_{12}L_6$)
M/Z 4+: (Cage Mass g/mol-(35.45 g/mol*4))/4
M/Z 3+: (Cage Mass g/mol-(35.45 g/mol*4)-1.008 g/mol)/3
M/Z 2+: (Cage Mass g/mol-(35.45 g/mol*4)-(1.008 g/mol*2))/2

Boat ($Zr_6L_2$)
M/Z 2+: (Cage Mass g/mol-(35.45 g/mol*2))/2
M/Z 1+: Cage Mass g/mol-(35.45 g/mol*2)-1.008 g/mol

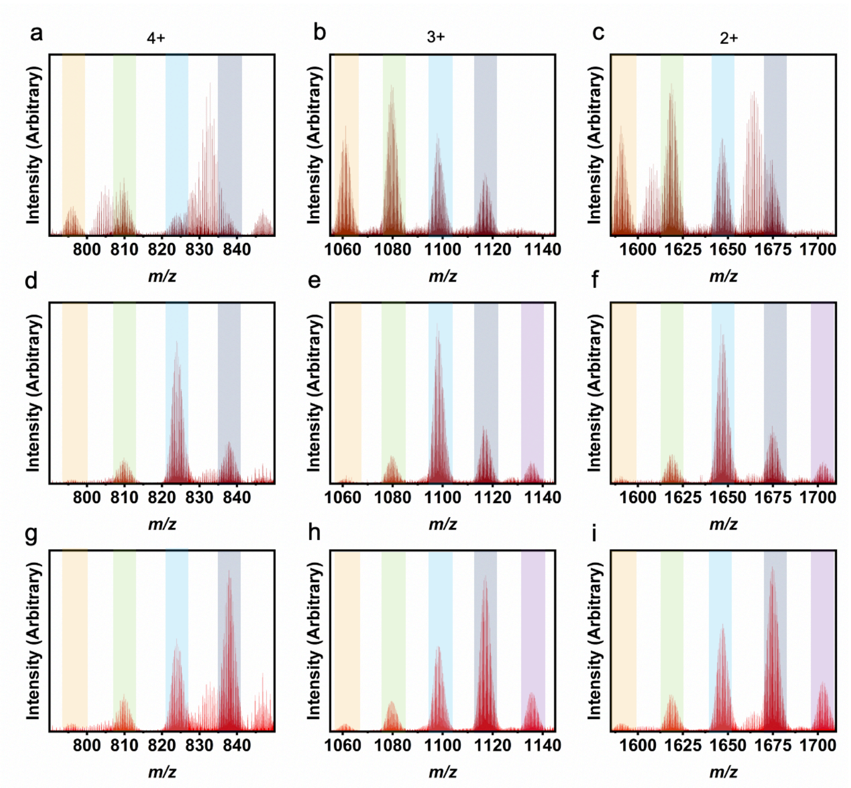


**Figure 49.** LC-MS results for heteroleptic 1,3-bdc: 5-*tert*-butyl cages showing $L^1$:$L^2$ ratios of 4:2 (a-c), 3:3 (d-f), and 2:4 (g-i). Column 1 depicts the 4+ M/Z region, column 2 the 3+ M/Z region, and column 3 the 2+ M/Z region. While the highest peak in each plot indicates the observed ligand ratio for the cage material, other ratios can also be seen. Orange columns show peaks for 5:1, green for 4:2, blue for 3:3, indigo for 2:4, and purple for 1:5.

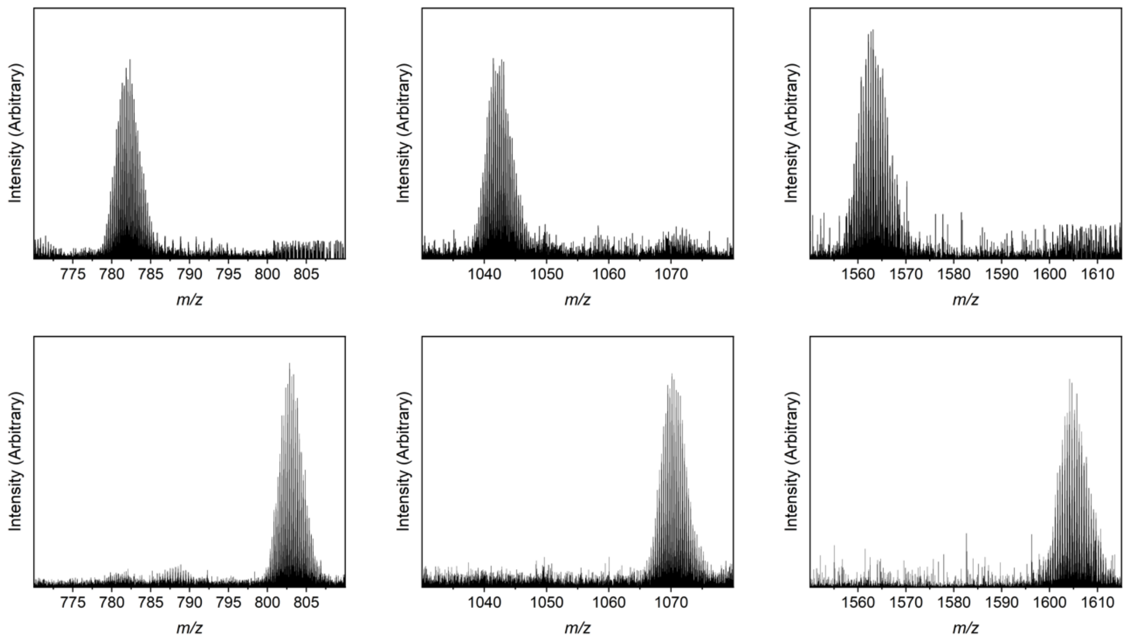


**Figure 50.** LC-MS spectra for homoleptic cages with $Zr_{12}L_6$ systems for 1,3-bdc (top) and 5-methyl (bottom).

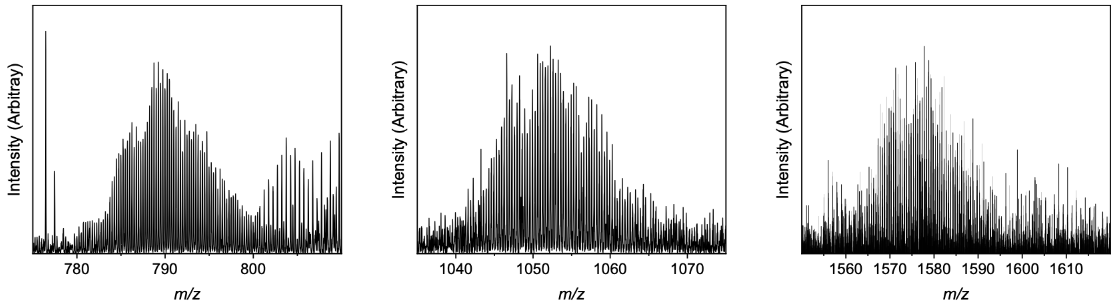


**Figure 51.** LC-MS spectra for heteroleptic cages with a $Zr_{12}L_6$ system for ($L^1$: $L^2$) 1,3-bdc: 5-OH.

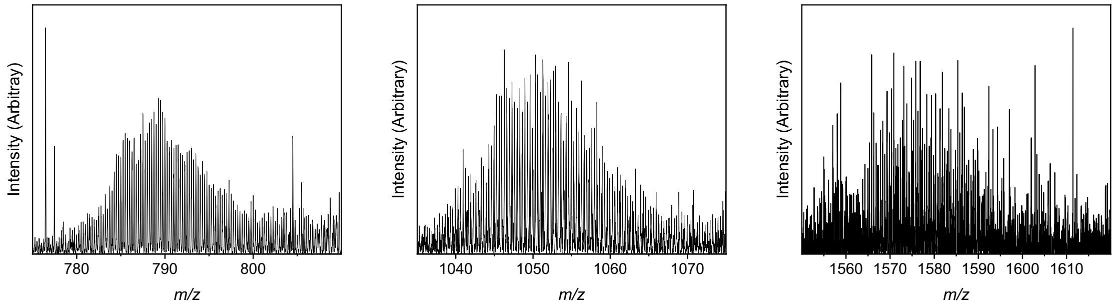


**Figure 52.** LC-MS spectra for heteroleptic cages with a $Zr_{12}L_6$ system for ($L^1$: $L^2$) 1,3-bdc: 5-$NH_2$.

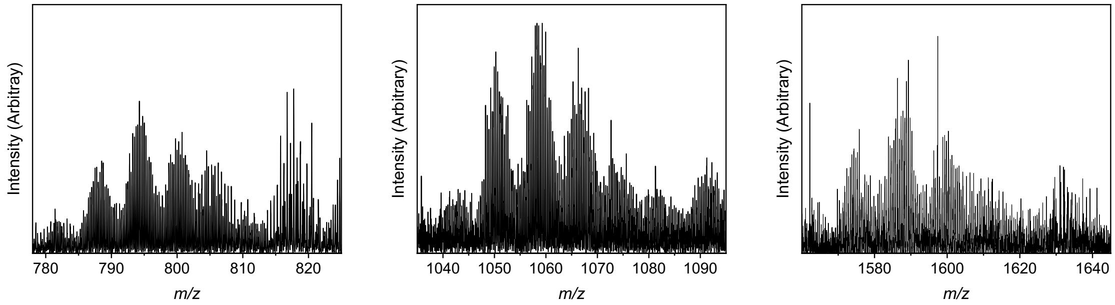


**Figure 53.** LC-MS spectra for heteroleptic cages with a $Zr_{12}L_6$ system for ($L^1$: $L^2$) 1,3-bdc: 5-CN.

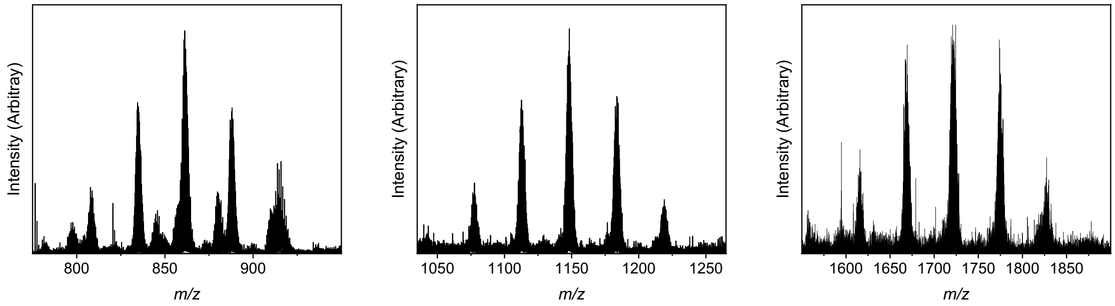


**Figure 54.** LC-MS spectra for heteroleptic cages with a $Zr_{12}L_6$ system for ($L^1$: $L^2$) 1,3-bdc: 5-benzyloxy.

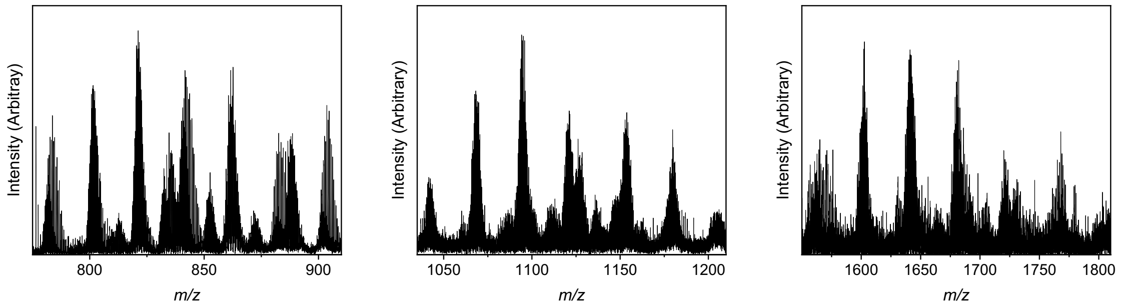


**Figure 55.** LC-MS spectra for heteroleptic cages with a $Zr_{12}L_6$ system for ($L^1$: $L^2$) 1,3-bdc: 5-Br.

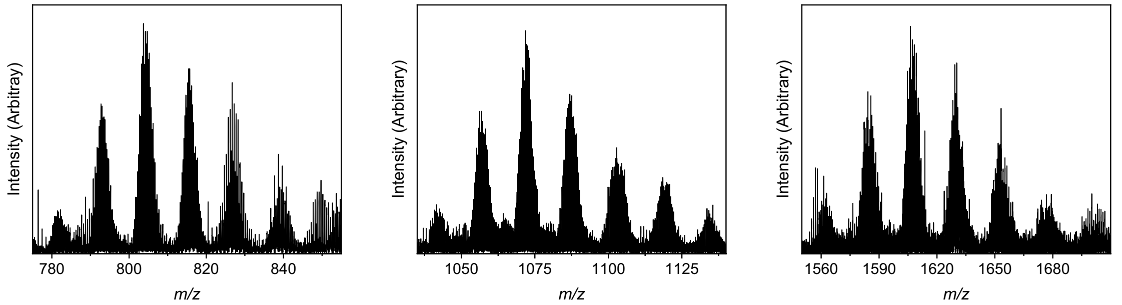


**Figure 56.** LC-MS spectra for heteroleptic cages with a $Zr_{12}L_6$ system for ($L^1$: $L^2$) 1,3-bdc: 5-$NO_2$.

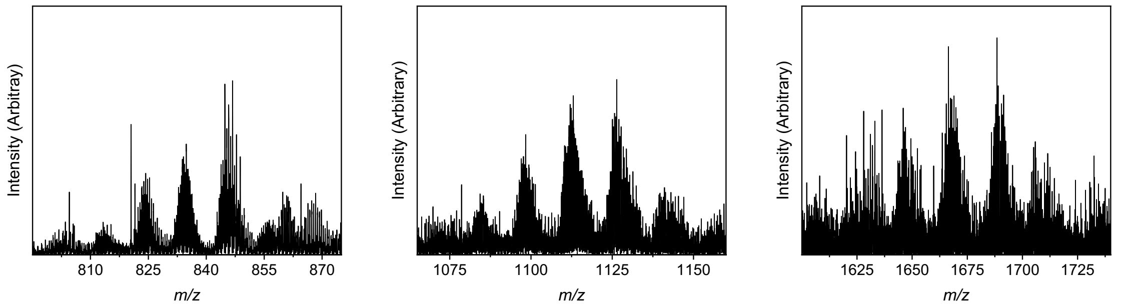


**Figure 57.** LC-MS spectra for heteroleptic cages with a $Zr_{12}L_6$ system for ($L^1$: $L^2$) 5-methyl: 5-*tert*-butyl.

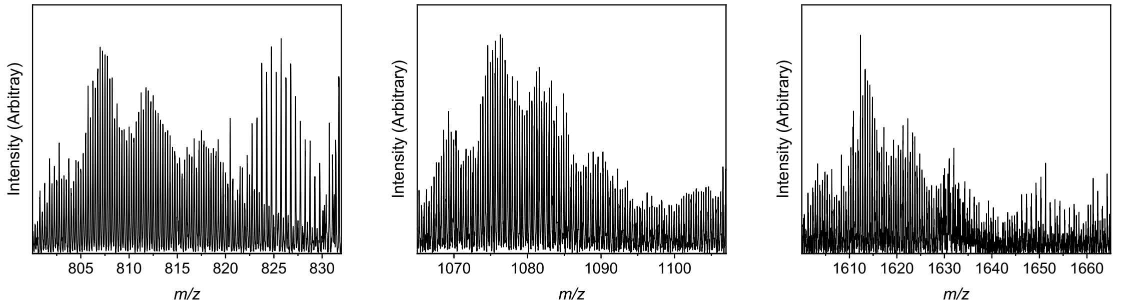


**Figure 58.** LC-MS spectra for heteroleptic cages with a $Zr_{12}L_6$ system for ($L^1$: $L^2$) 5-methyl: *m*-dobdc.

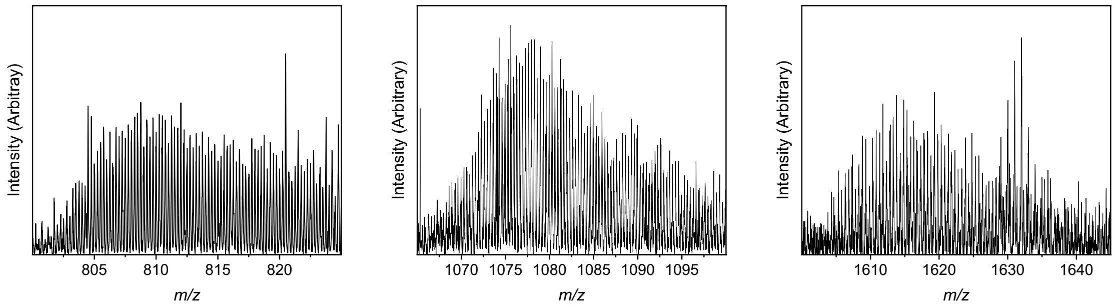


**Figure 59.** LC-MS spectra for heteroleptic cages with a $Zr_{12}L_6$ system for ($L^1$: $L^2$) 5-methyl: 5-CN.

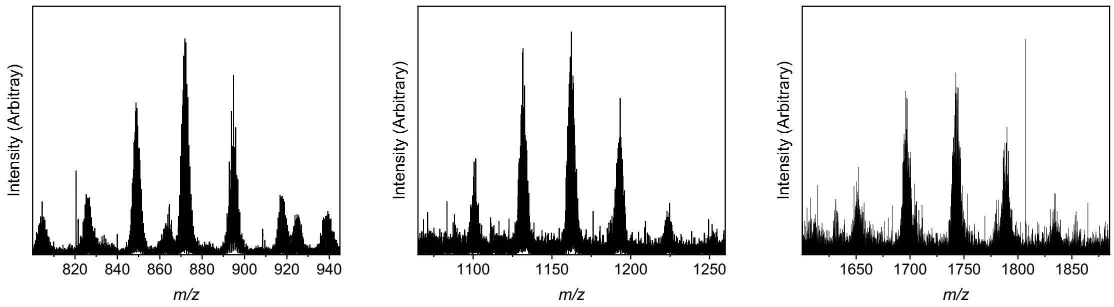


**Figure 60.** LC-MS spectra for heteroleptic cages with a $Zr_{12}L_6$ system for ($L^1$: $L^2$) 5-methyl: 5-benzyloxy.

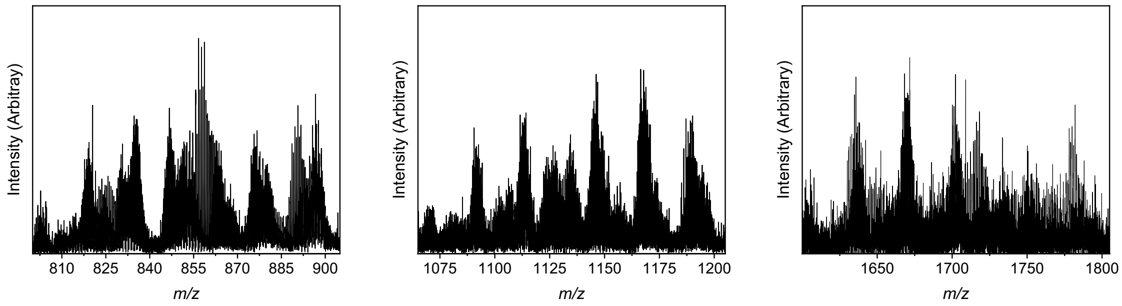


**Figure 61.** LC-MS spectra for heteroleptic cages with a $Zr_{12}L_6$ system for ($L^1$: $L^2$) 5-methyl: 5-Br.

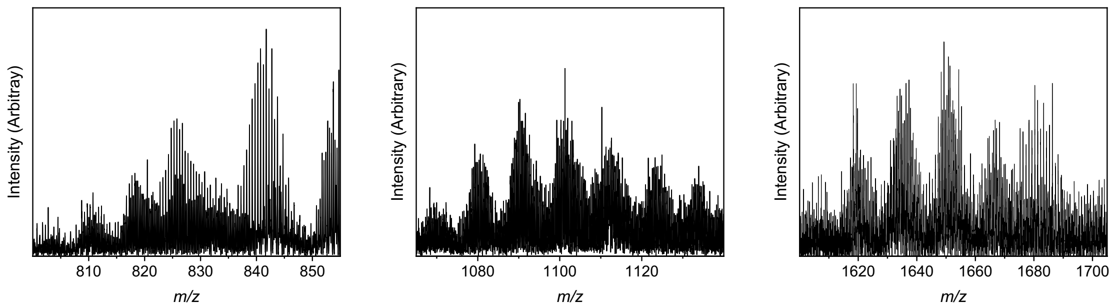


**Figure 62.** LC-MS spectra for heteroleptic cages with a $Zr_{12}L_6$ system for ($L^1$: $L^2$) 5-methyl: 5-$NO_2$.

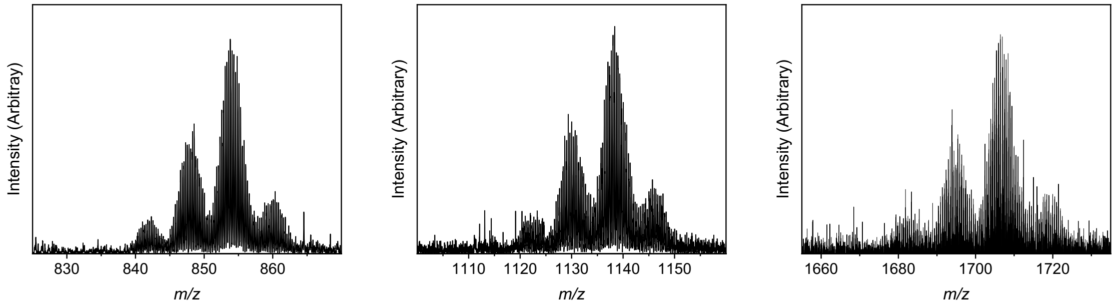


**Figure 63.** LC-MS spectra for heteroleptic cages with a $Zr_{12}L_6$ system for ($L^1$: $L^2$) 5-*tert*-butyl: *m*-dobdc.

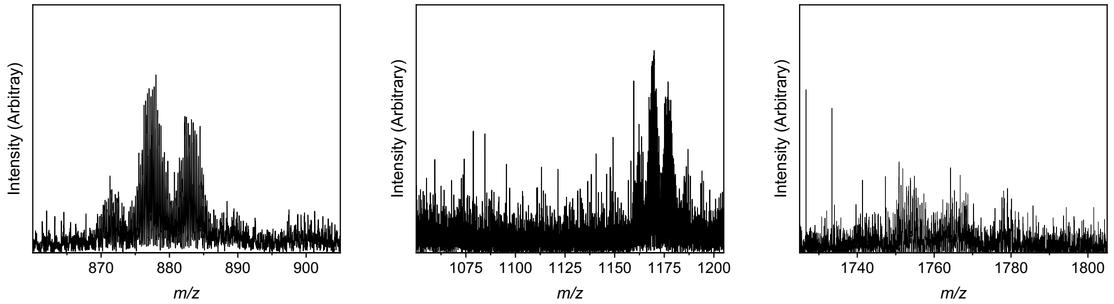


**Figure 64.** LC-MS spectra for heteroleptic cages with a $Zr_{12}L_6$ system for ($L^1$: $L^2$) 5-*tert*-butyl: 5-Br.

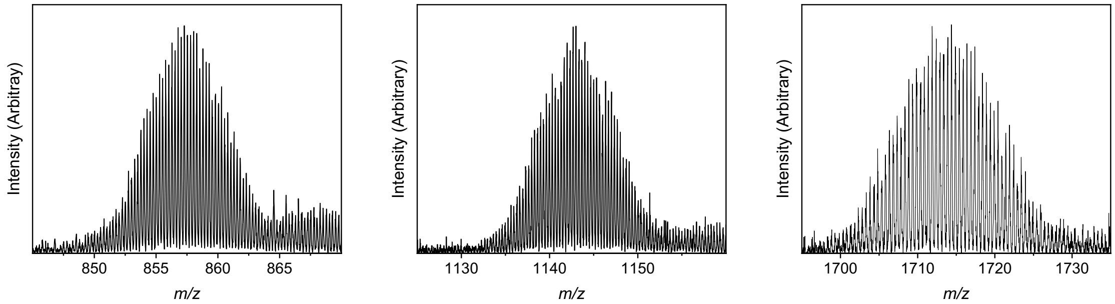


**Figure 65.** LC-MS spectra for heteroleptic cages with a $Zr_{12}L_6$ system for ($L^1$: $L^2$) 5-*tert*-butyl: 5-$NO_2$.

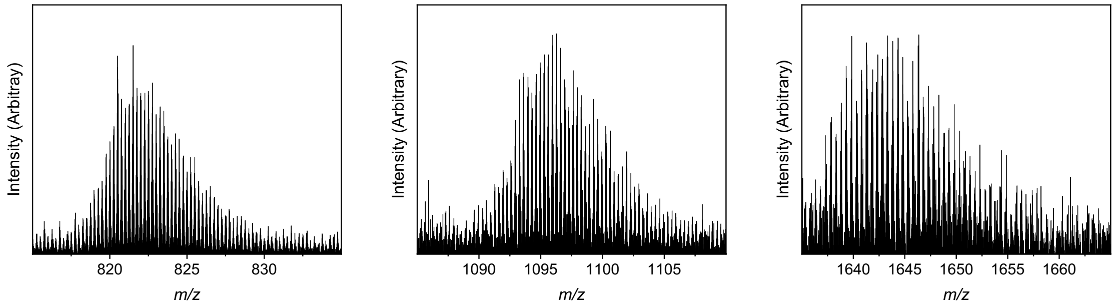


**Figure 66.** LC-MS spectra for heteroleptic cages with a $Zr_{12}L_6$ system for ($L^1$: $L^2$) *m*-dobdc: 5-CN.

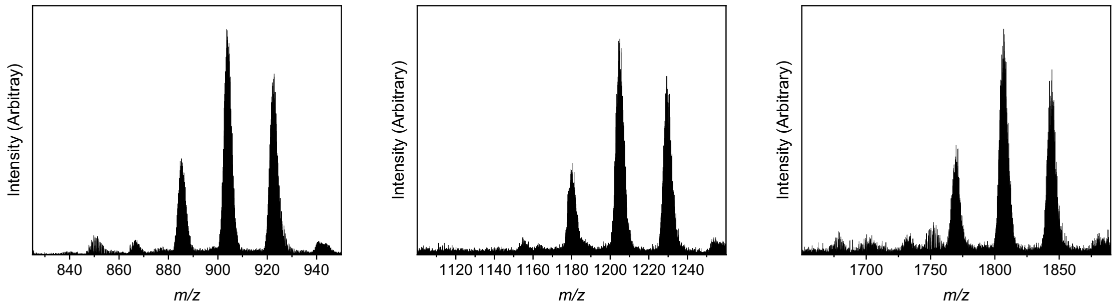


**Figure 67.** LC-MS spectra for heteroleptic cages with a $Zr_{12}L_6$ system for ($L^1$: $L^2$) *m*-dobdc: 5-benzyloxy.

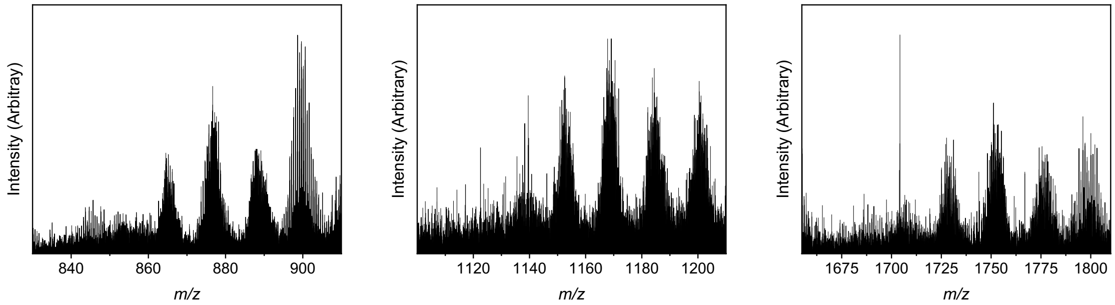


**Figure 68.** LC-MS spectra for heteroleptic cages with a $Zr_{12}L_6$ system for ($L^1$: $L^2$) *m*-dobdc: 5-Br.

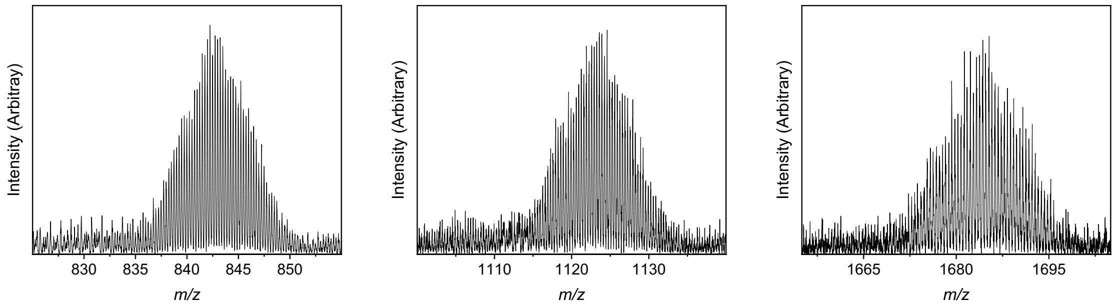


**Figure 69.** LC-MS spectra for heteroleptic cages with a $Zr_{12}L_6$ system for ($L^1$: $L^2$) *m*-dobdc: 5-$NO_2$.

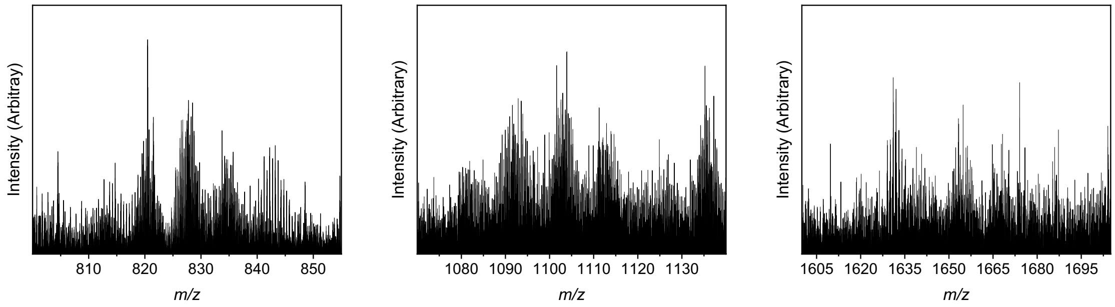


**Figure 70.** LC-MS spectra for heteroleptic cages with a $Zr_{12}L_6$ system for ($L^1$: $L^2$) 5-OH: 5-$NO_2$.

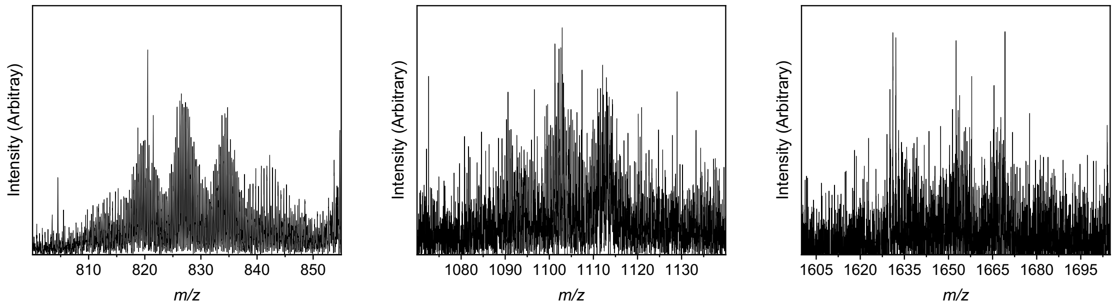


**Figure 71.** LC-MS spectra for heteroleptic cages with a $Zr_{12}L_6$ system for ($L^1$: $L^2$) 5-$NH_2$: 5-$NO_2$.

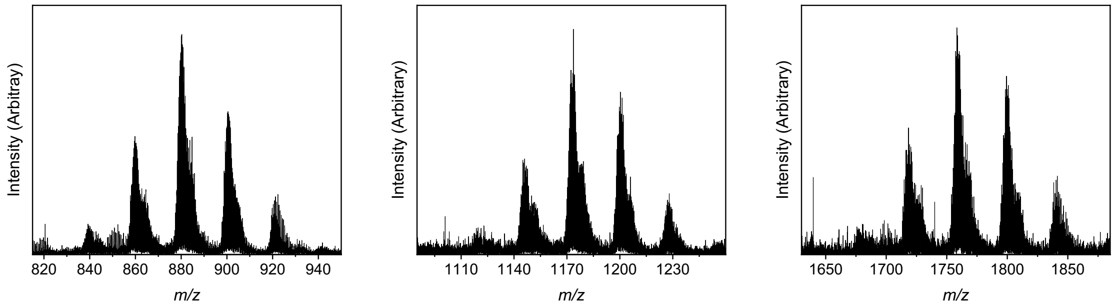


**Figure 72.** LC-MS spectra for heteroleptic cages with a $Zr_{12}L_6$ system for ($L^1$: $L^2$) 5-CN: 5-benzyloxy.

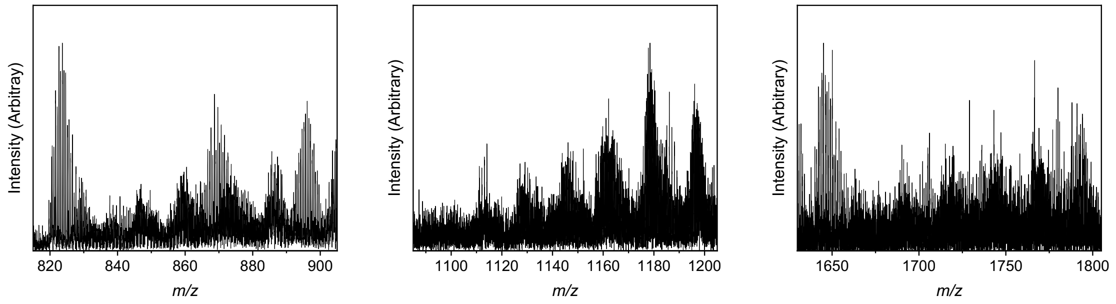


**Figure 73.** LC-MS spectra for heteroleptic cages with a $Zr_{12}L_6$ system for ($L^1$: $L^2$) 5-CN: 5-Br.

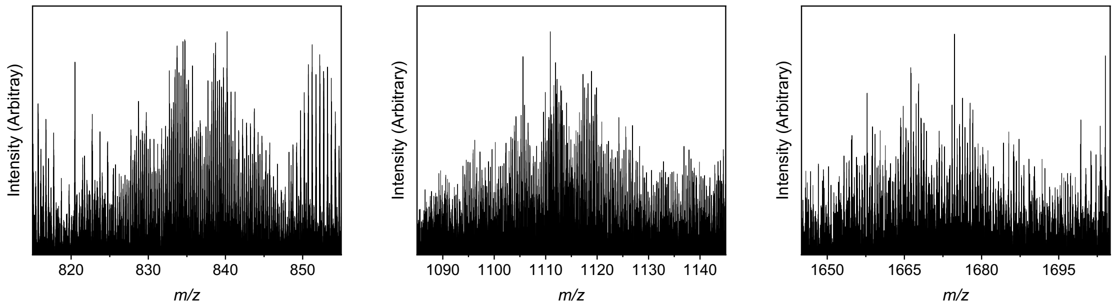


**Figure 74.** LC-MS spectra for heteroleptic cages with a $Zr_{12}L_6$ system for ($L^1$: $L^2$) 5-CN: 5-$NO_2$.

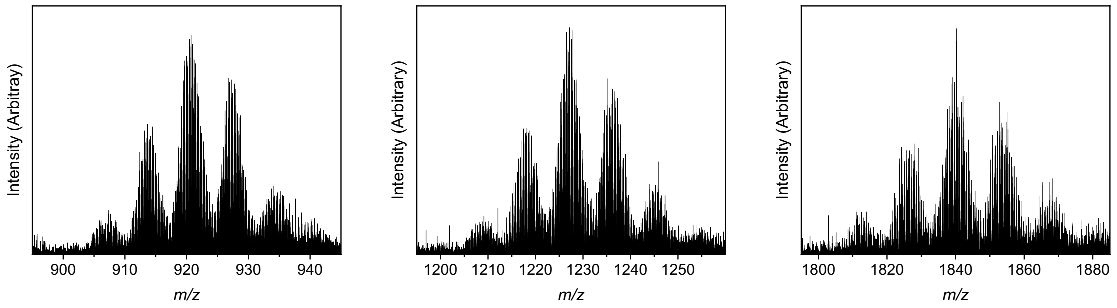


**Figure 75.** LC-MS spectra for heteroleptic cages with a $Zr_{12}L_6$ system for ($L^1$: $L^2$) 5-benzyloxy: 5-Br.

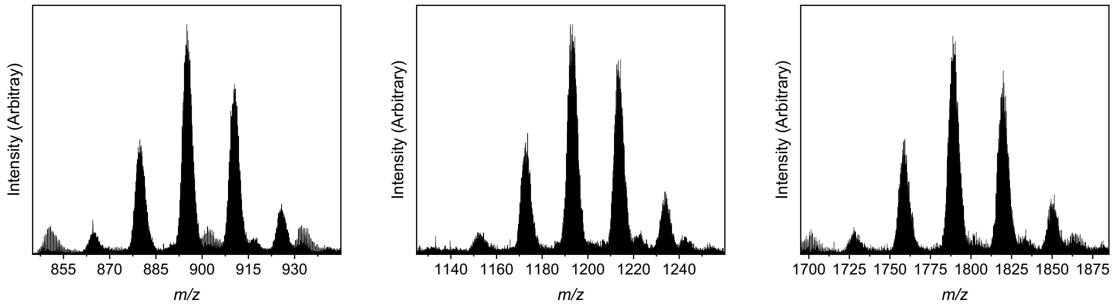


**Figure 76.** LC-MS spectra for heteroleptic cages with a $Zr_{12}L_6$ system for ($L^1$: $L^2$) 5-benzyloxy: 5-$NO_2$.

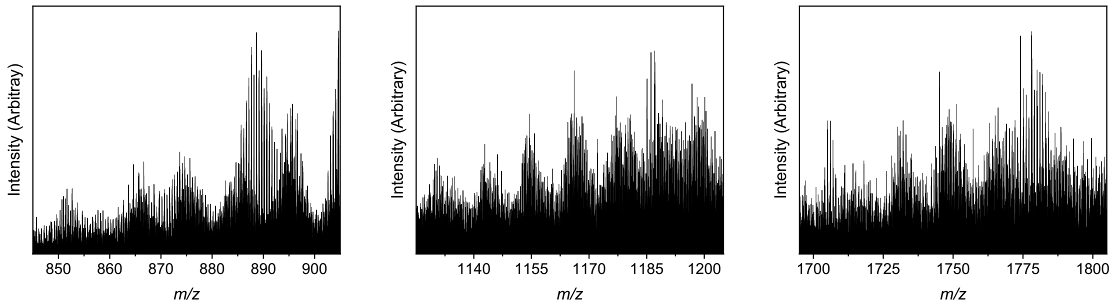


**Figure 77.** LC-MS spectra for heteroleptic cages with a $Zr_{12}L_6$ system for ($L^1$: $L^2$) 5-Br: 5-$NO_2$.

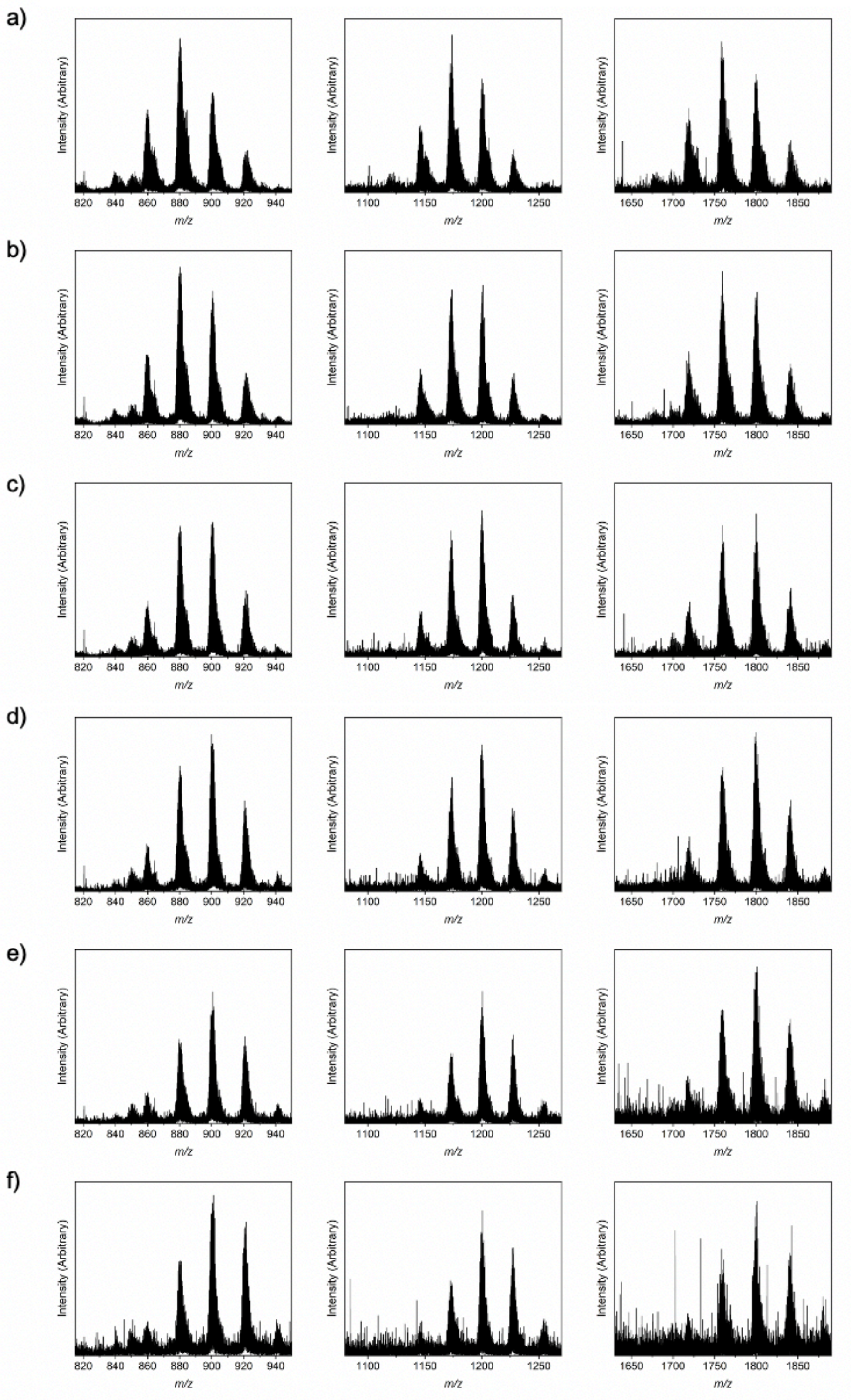


**Figure 78.** LC-MS spectra for 3:3 5-CN: 5-benzyloxy taken at different elution times from the chromatogram: 2.193 min (a), 2.222 min (b), 2.251 min (c), 2.280 min (d), 2.309 min (e), and 2.338 min (f).

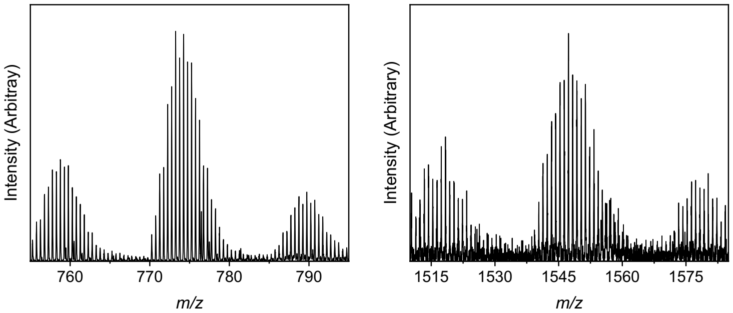


**Figure 79.** LC-MS spectra for heteroleptic cages with a $Zr_6L_2$ system for ($L^1$: $L^2$) 5-methyl: 5-$NO_2$.

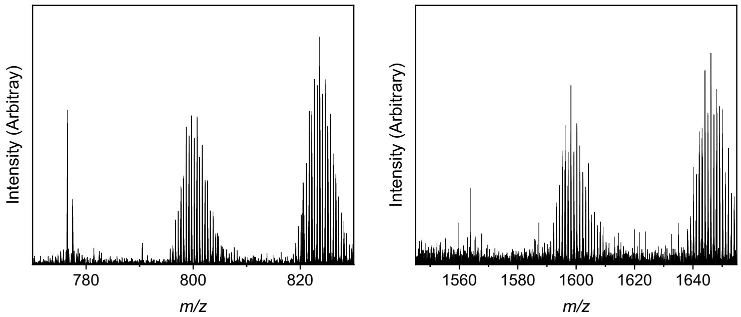


**Figure 80.** LC-MS spectra for heteroleptic cages with a $Zr_6L_2$ system for ($L^1$: $L^2$) *m*-dobdc: 5-Br.

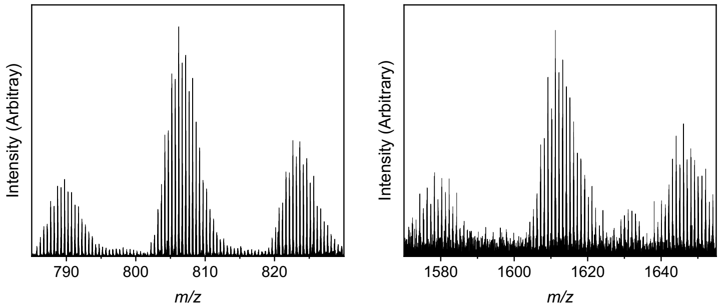


**Figure 81.** LC-MS spectra for heteroleptic cages with a $Zr_6L_2$ system for ($L^1$: $L^2$) 5-Br: 5-$NO_2$.

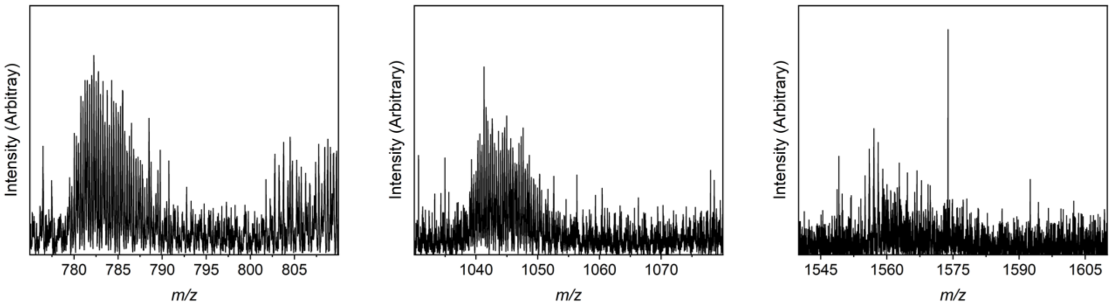


**Figure 82.** LC-MS spectra for control experiment for $Zr_{12}$1,3-bdc$_6$ + 5-methyl-1,3-bdc.

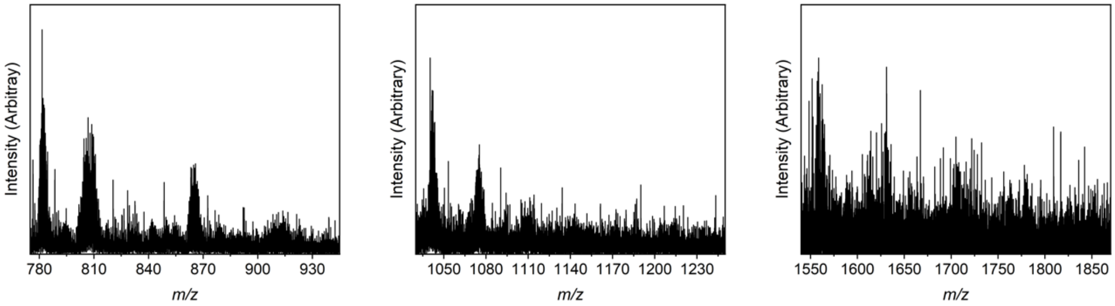


**Figure 83.** LC-MS spectra for control experiment for $Zr_{12}$1,3-$bdc_6$ + 5-MPA-1,3-bdc.

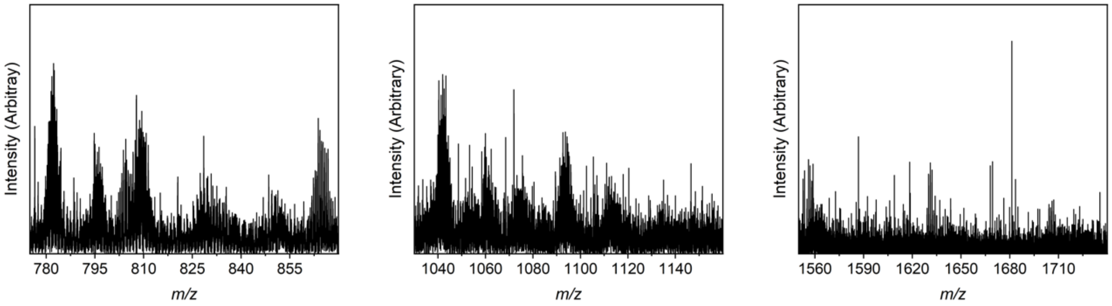


**Figure 84.** LC-MS spectra for control experiment for $Zr_{12}$1,3-$bdc_6$ + 5-*tert*-butyl-1,3-bdc.

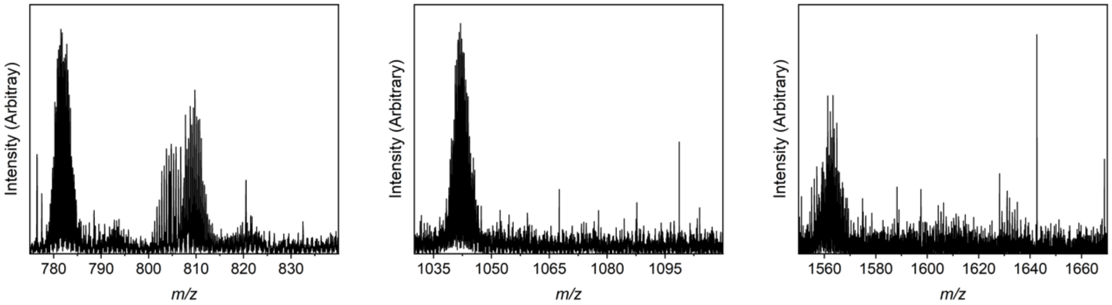


**Figure 85.** LC-MS spectra for control experiment for $Zr_{12}$1,3-$bdc_6$ + *m*-dobdc.

## Detailed Single Crystal X-Ray Diffraction Information and Crystallographic Information

X-ray structural analysis for **$[Zr_6(5\text{-MPA-1,3-bdc})_2]^{2+}$**: Crystals were mounted using viscous oil onto a plastic mesh and cooled to the data collection temperature. Data were collected on a D8 Venture Photon III diffractometer with Cu-Kα radiation (λ = 1.54178 Å) focused with Goebel mirrors. Unit cell parameters were obtained from fast scan data frames, 1º/s ω, of an Ewald hemisphere. The unit-cell dimensions, equivalent reflections and systematic absences in the diffraction data are consistent with Pna21 and Pnma for **$[Zr_6(5\text{-MPA-1,3-bdc})_2]^{2+}$**. Refinement in the centrosymmetric space group yielded chemically reasonable and computationally stable results of refinement. The data were treated with multi-scan absorption corrections.[6] Structure was solved using intrinsic phasing methods[7] and refined with full-matrix, least-squares procedures on $F^2$.[8] These compounds apparently consistently deposited as weakly diffracting crystals an the data represent the best of several trials.

For **$[Zr_6(5\text{-MPA-1,3-bdc})_2]^{2+}$** the structure is located at an inversion center. A molecule of DMF solvent was treated as a diffused contribution consistent with the electron counts from the Squeeze results.[9]

Non-hydrogen atoms were refined with anisotropic displacement parameters. All hydrogen atoms were treated as idealized contributions with geometrically calculated positions and with $U_{iso}$ equal to 1.2 $U_{eq}$ (1.5 $U_{eq}$ for methyl) of the attached atom.

Atomic scattering factors are contained in the SHELXTL program library.[7] The structures have been deposited at the Cambridge Structural Database under the following CCDC depositary numbers: CCDC 2321239.

**Table S6.** Crystal data and structure refinement details.

| | |
|---|---|
| Compound | $[Zr_6(5\text{-MPA-1,3-bdc})_2]^{4+}$ |
| Empirical Formula | $C_{70}Cl_2H_{92}N_6O_{26}Zr_6$ |
| Formula Weight, g/mol | 2051.71 |
| Temperature, K | 120.0 |
| Crystal System | orthorhombic |
| Space Group | *Pnma* |
| Cell dimensions | |
| *a*, Å | 14.0277(6) |
| *b*, Å | 31.9593(12) |
| *c*, Å | 20.8847(8) |
| α, ° | 90 |
| β, ° | 90 |
| γ, ° | 90 |

| | |
|---|---|
| Volume, Å$^3$ | 9362.9(6) |
| *Z* | 4 |
| $\rho_{calc}$ g/cm$^3$ | 1.456 |
| μ/mm$^{-1}$ | 6.390 |
| F(000) | 4144.0 |
| Reflections collected | 43756 |
| Independant reflections | 6412 |
| Data/restraints/parameters | 6412/414/473 |
| Goodness-of-fit | 1.170 |
| R [I>=2σ (I)] $R_1/wR_2$ | 0.1118/0.2385 |
| R indexes [all data] $R_1/wR_2$ | 0.1161/0.2408 |
| CCDC | 2321239 |

## Thermogravimetric Analysis (TGA)

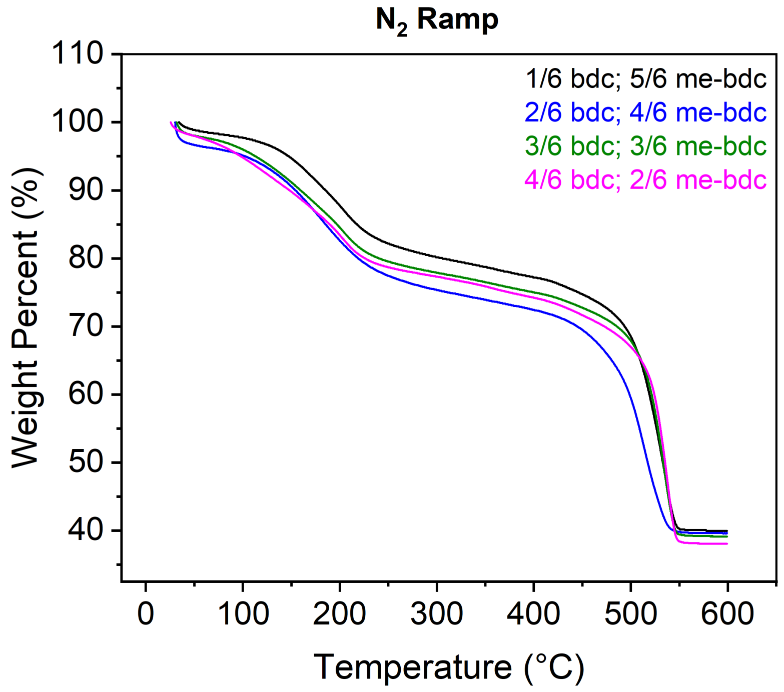


**Figure 86.** TGA results for 1,3-bdc: 5-methyl heteroleptic cages heated at 2° / minute under flowing $N_2$.

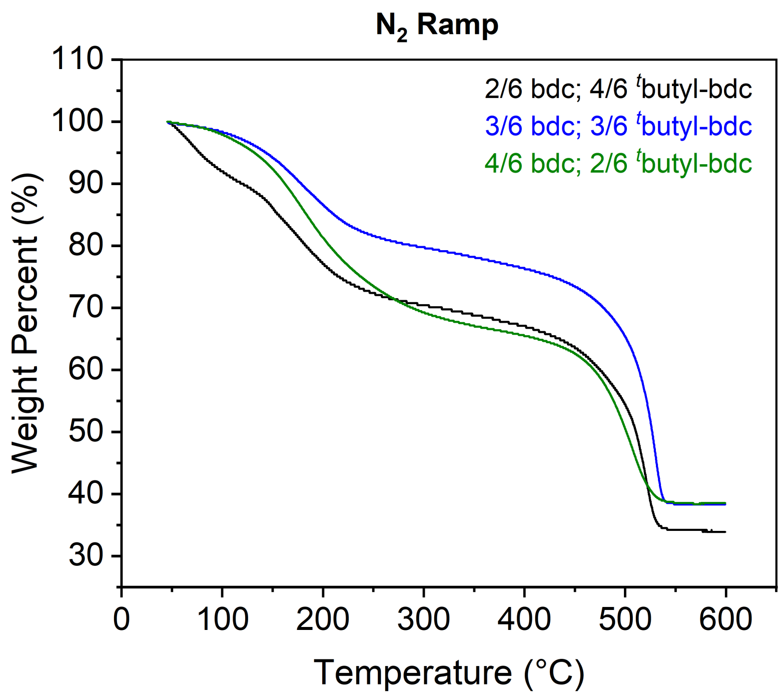


**Figure 87.** TGA results for 1,3-bdc: 5-*tert*-butyl heteroleptic cages heated at 2° / minute under flowing $N_2$.

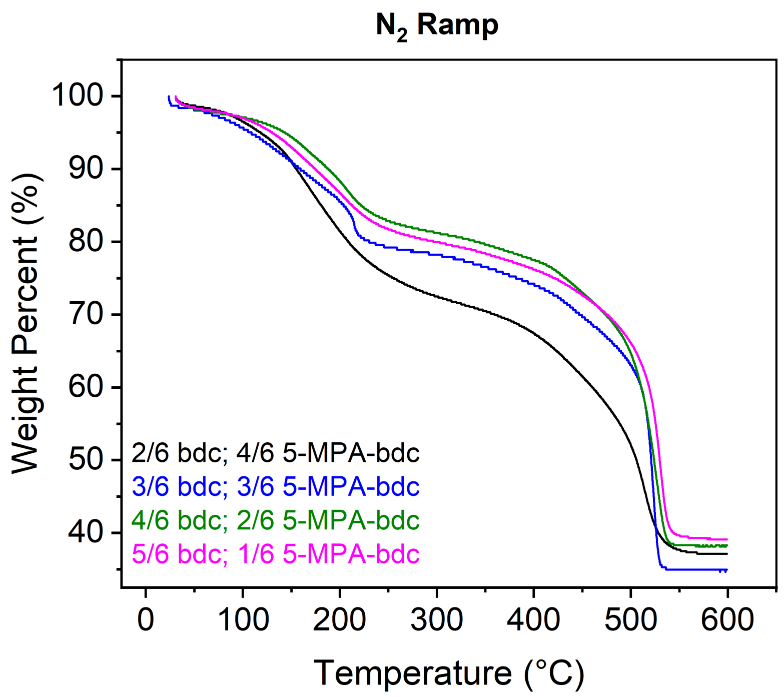


**Figure 88.** TGA results for 1,3-bdc: 5-MPA heteroleptic cages heated at 2° / minute under flowing $N_2$.

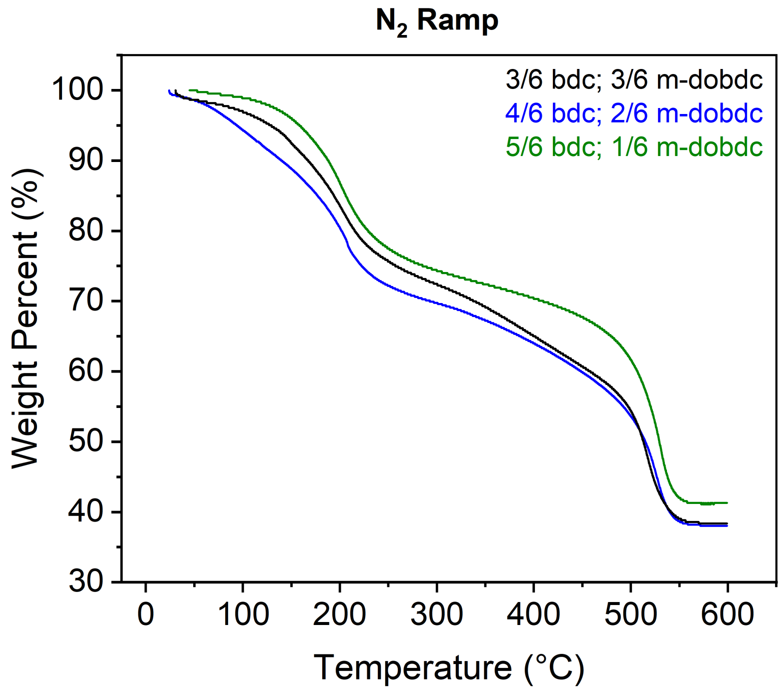


**Figure 89.** TGA results for 1,3-bdc: *m*-dobdc heteroleptic cages heated at 2° / minute under flowing $N_2$.

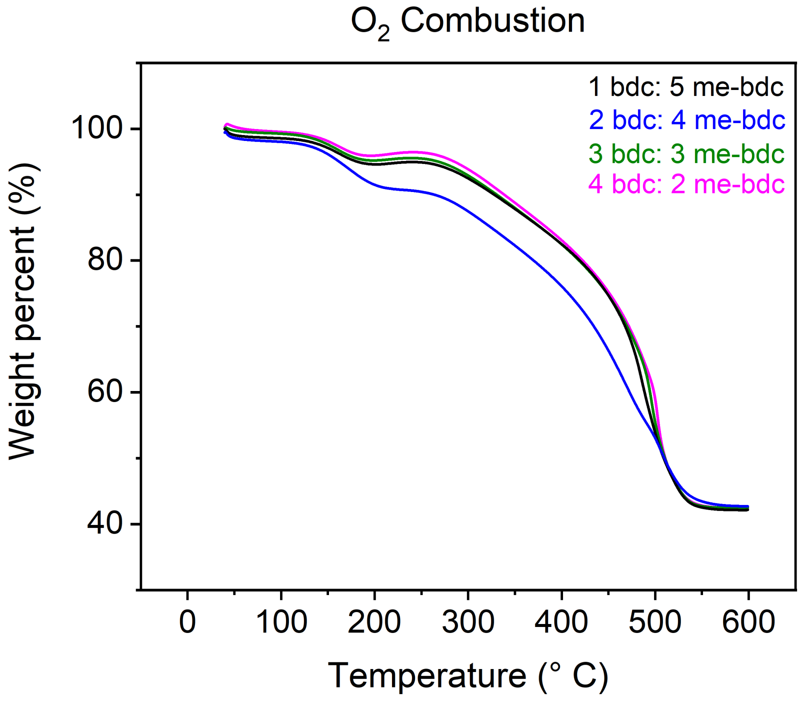


**Figure 90.** TGA results for 1,3-bdc: 5-methyl heteroleptic cages heated at 2° / minute under flowing $O_2$.

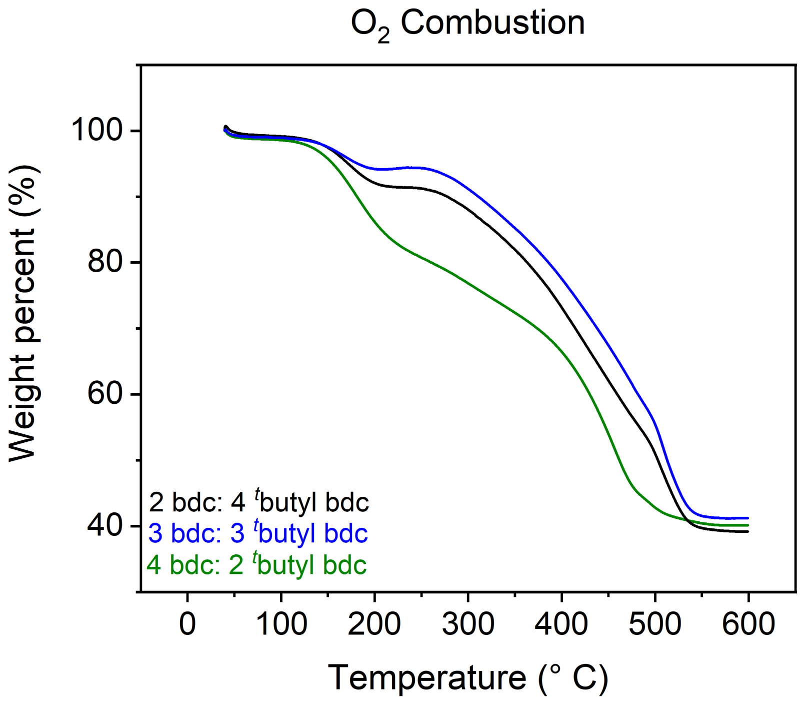


**Figure 91.** TGA results for 1,3-bdc: 5-*tert*-butyl heteroleptic cages heated at 2° / minute under flowing $O_2$.

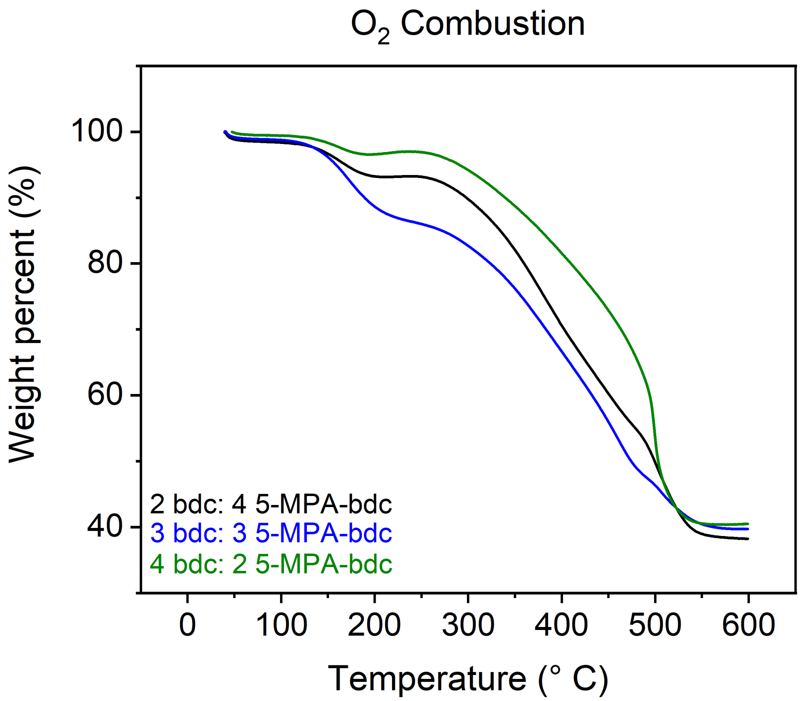


**Figure 92.** TGA results for 1,3-bdc: 5-MPA heteroleptic cages heated at 2° / minute under flowing $O_2$.

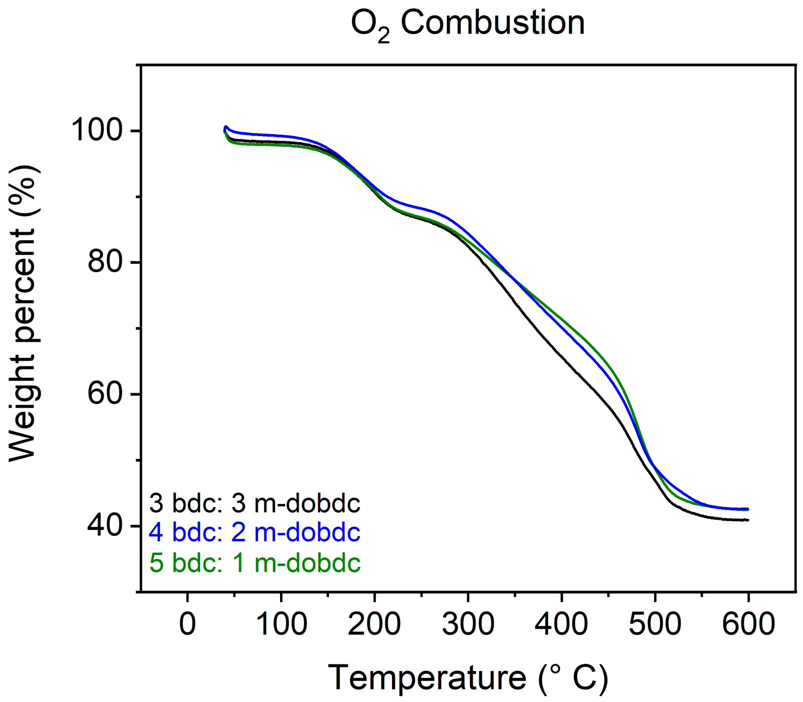


**Figure 93.** TGA results for 1,3-bdc: *m*-dobdc heteroleptic cages heated at 2° / minute under flowing $O_2$.

## Click Reaction

### Infrared Spectroscopy (IR)

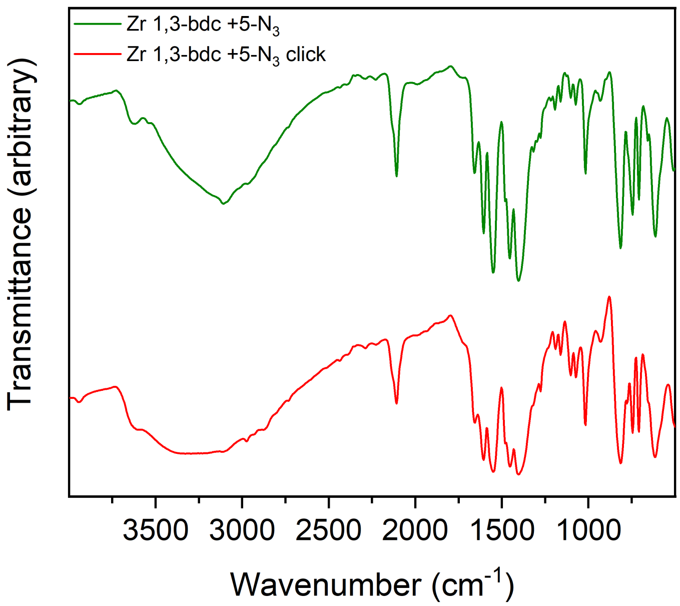


**Figure 94.** IR spectra of the 1,3-bdc :5-$N_3$ heteroleptic cage (green) and the 1,3-bdc: 5-$N_3$-PrOH clicked cage (red).

## LC-MS

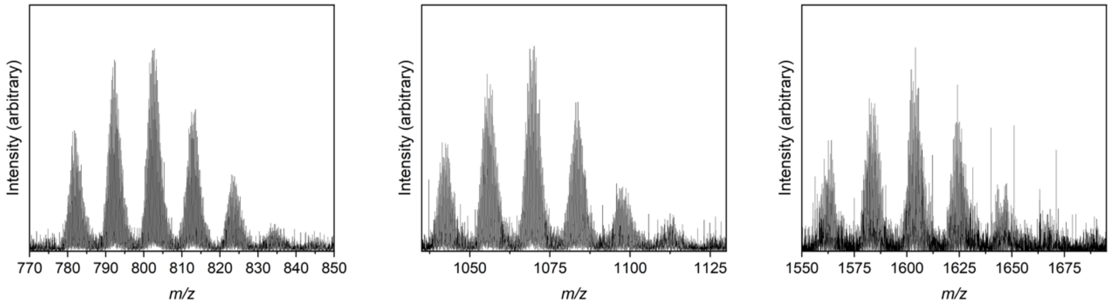


**Figure 95.** LC-MS spectra of the 1,3-bdc: 5-$N_3$ heteroleptic cage with a $Zr_{12}L_6$ system.

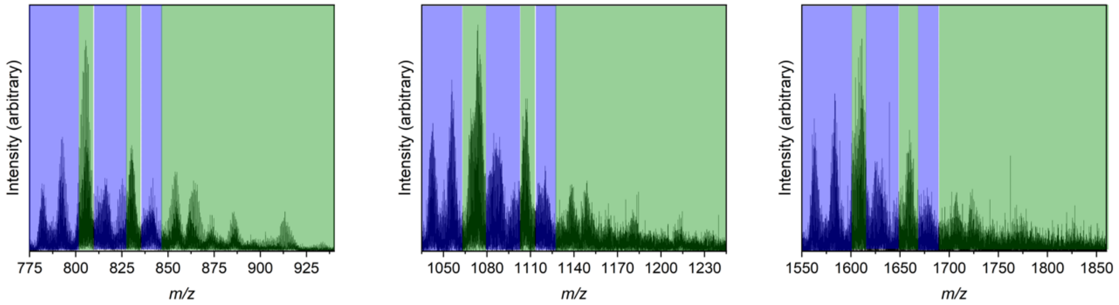


**Figure 96.** LC-MS spectra of the 1,3-bdc: 5-$N_3$-PrOH clicked product where the peaks highlighted in blue correspond to unreacted, unclicked heteroleptic cage while the peaks highlighted in green correspond to fully clicked heteroleptic cages.

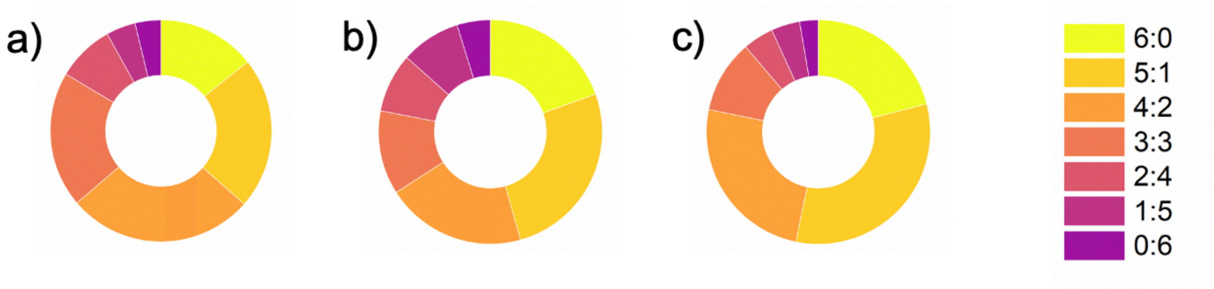


**Figure 97.** Donut plots for *M/Z* peaks ±0.5 *M/Z* away from the calculated value taken from the LC-MS spectra of the 1,3-bdc: 5-$N_3$ heteroleptic cage (a), the blue highlighted peaks of the LC-MS spectra for the 1,3-bdc: 5-$N_3$-PrOH clicked cage (b), and the green highlighted peaks of the LC-MS spectra for the 1,3-bdc: 5-$N_3$-PrOH fully clicked cage (c).

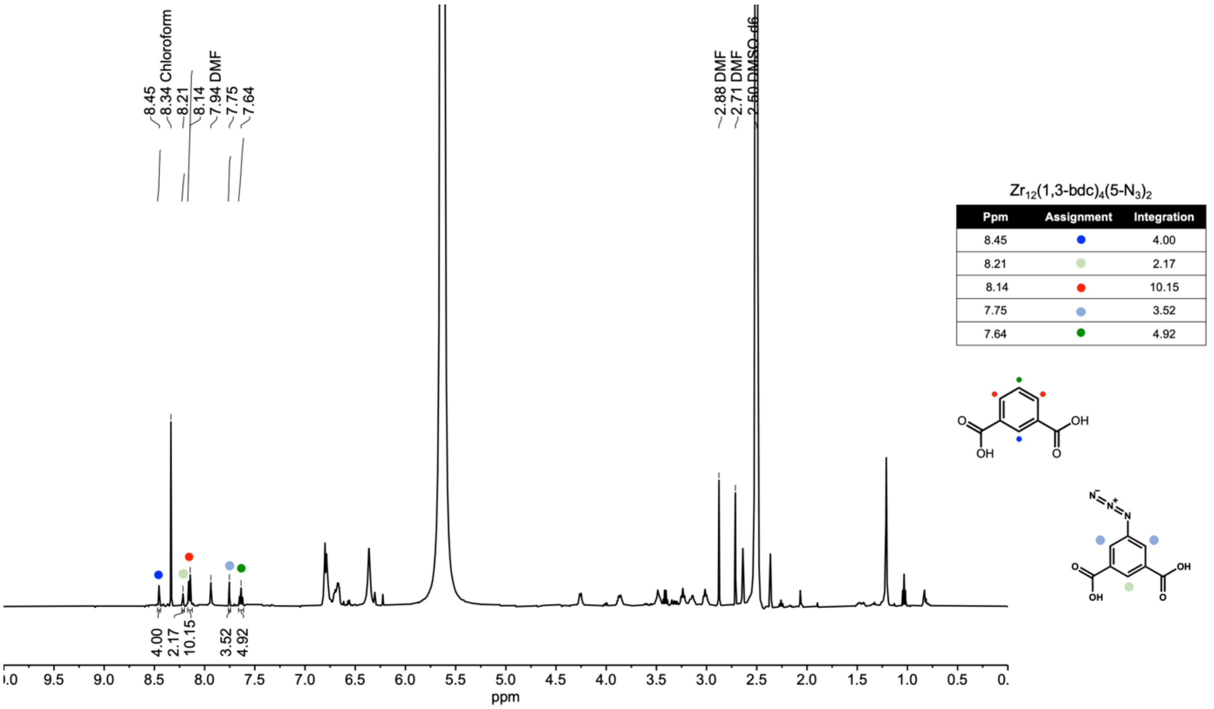


$Zr_{12}(1,3\text{-bdc})_4(5\text{-}N_3)_2$

| Ppm | Assignment | Integration |
|---|---|---|
| 8.45 | ● | 4.00 |
| 8.21 | ● | 2.17 |
| 8.14 | ● | 10.15 |
| 7.75 | ● | 3.52 |
| 7.64 | ● | 4.92 |

**Figure 98.** $^1$H NMR for the heteroleptic 1,3-bdc: 5-$N_3$ cage.

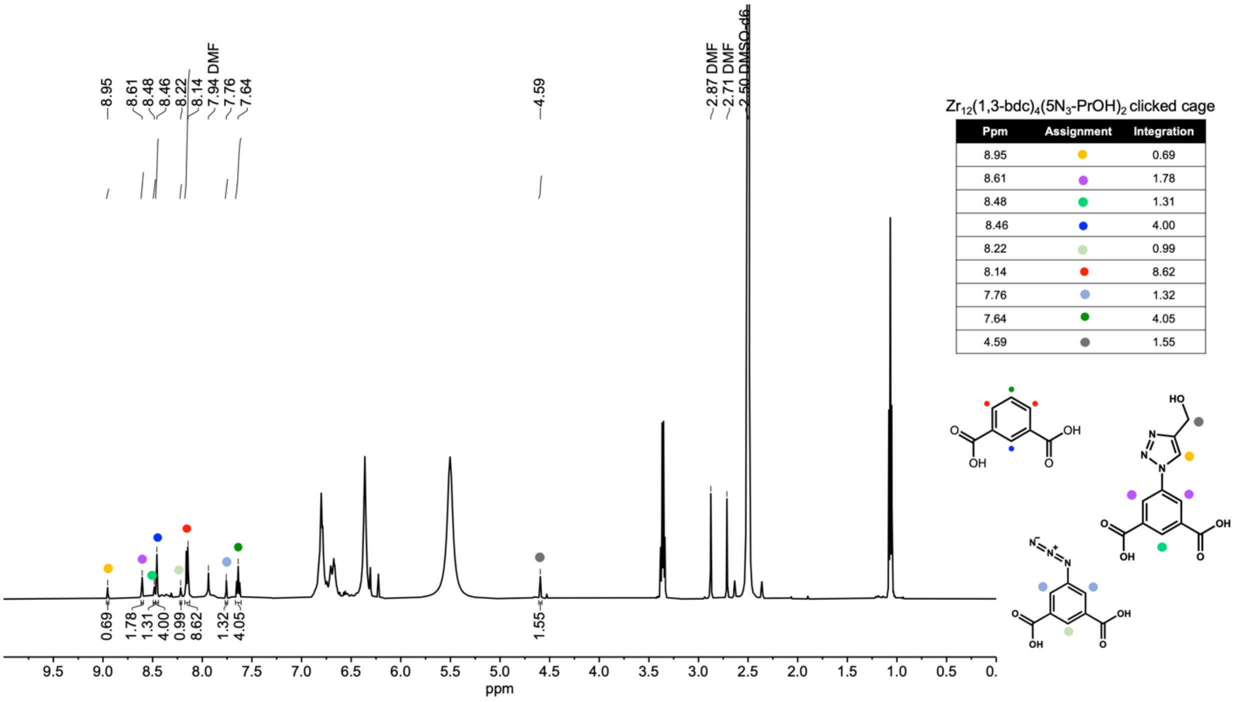


$Zr_{12}(1,3\text{-bdc})_4(5N_3\text{-PrOH})_2$ clicked cage

| Ppm | Assignment | Integration |
|---|---|---|
| 8.95 | ● | 0.69 |
| 8.61 | ● | 1.78 |
| 8.48 | ● | 1.31 |
| 8.46 | ● | 4.00 |
| 8.22 | ● | 0.99 |
| 8.14 | ● | 8.62 |
| 7.76 | ● | 1.32 |
| 7.64 | ● | 4.05 |
| 4.59 | ● | 1.55 |

**Figure 99.** $^1$H NMR for the heteroleptic 1,3-bdc: 5-$N_3$-PrOH clicked cage product.

## References

1. M. Loos, C. Gerber, F. Corona, J. Hollender and H. Singer, *Anal. Chem*, 2015, **87**, 5738–5744.
2. M. T. Kapelewski, T. Runčevski, J.D. Tarver, H. Z. H. Jiang, K. E. Hurst, P. A. Parilla, A. Ayala, T. Gennett, S. A. FitzGerald, C. M. Brown and J. R. Long, *Chem. Mater,* 2018, **30**, 8179-8189.
3. A. M. Antonio, K. J. Korman, M. M. Deegan, G. A. Taggart, G. P. A. Yap and E. D. Bloch, *Inorg. Chem*. 2022, **61**, 4609–4617.
4. M. R. Dworzak, C. M. Montone, N. I. Halaszynski, G. P. A. Yapp, C. J. Kloxin and E. D. Bloch, *Chem. Commun.*, 2023, **59**, 8977-8980.
5. S. Du, X. Yu, G.Liu, M. Zhou, E.-S. M. El-Sayed, Z. Ju, K. Su and D. Yuan *Crystal Growth & Design,* 2021, **21**, 692-697.
6. Apex4, Bruker AXS Inc., Madison, WI, USA, 2021.
7. G. M. Sheldrick, *Acta Cryst.* 2015, **A71**, 3–8. DOI: 10.1107/S2053273314026370
8. G. M. Sheldrick, *Acta Cryst.* 2015, **C71**, 3–6. DOI: 10.1107/S2053229614024218
9. A. L. Spek, *Acta Cryst.* 2015, **C71**, 9–18. DOI: 10.1107/S2053229614024929
